# YAP1 induces bladder cancer progression and promotes immune evasion through IL-6/STAT3 pathway and CXCL deregulation

**DOI:** 10.1172/JCI171164

**Published:** 2024-11-21

**Authors:** Pritam Sadhukhan, Mingxiao Feng, Emily Illingworth, Ido Sloma, Akira Ooki, Andres Matoso, David Sidransky, Burles A. Johnson, Luigi Marchionni, Fenna C.M. Sillé, Woonyoung Choi, David McConkey, Mohammad O. Hoque

**Affiliations:** 1 Department of Otolaryngology–Head and Neck Surgery and; 2 Department of Urology, Johns Hopkins University School of Medicine, Baltimore, Maryland, USA.; 3 Department of Environmental Health and Engineering, Johns Hopkins Bloomberg School of Public Health, Baltimore, Maryland, USA.; 4 Champions Oncology, R&D, Baltimore, Maryland, USA.; 5 Department of Pathology and; 6 Department of Oncology, Johns Hopkins University School of Medicine, Baltimore, Maryland, USA.; 7 Pathology and Laboratory Medicine, Weill Cornell Medical College, New York, New York, USA.

**Keywords:** Oncology, Therapeutics, Cancer immunotherapy, Oncogenes, Tumor suppressors

## Abstract

The Hippo signaling pathway plays a key role in tumorigenesis in different cancer types. We investigated the role of the Hippo effector YAP1 in the tumor immune microenvironment (TIME) of urothelial carcinoma of the bladder (UCB) and evaluated the efficacy of immunotherapy in the context of YAP1 signaling. We performed numerous in vitro and in vivo experiments to determine the role of YAP1 using genetic and pharmacological attenuation of YAP1 activity. Briefly, RNA sequencing was carried out with mouse and human cell lines to identify novel YAP1-regulated downstream targets unbiasedly. We then experimentally confirmed that YAP1 regulates the TIME through the IL-6/STAT3 signaling pathway and varied C-X-C motif chemokine regulation. We analyzed several human sample sets to explore the TIME status in the context of YAP1 expression. Our data indicate that YAP1 attenuation decreases M2 macrophages and myeloid-derived suppressor cells in the TIME compared with YAP1-expressing cells. In summary, this study provides insights into YAP1 signaling as a driver for cancer stemness and an inducer of immunosuppressive TIME. Moreover, the therapeutic efficacy of YAP1 attenuation indicates that combined blockade of YAP1 and immune checkpoints may yield clinical value for treating patients with UCB.

## Abstract 2

In Bladder cancer, YAP1 signaling is found to drive cancer stemness and induce an immunosuppressive tumor microenvironment by influencing the infiltration of MDSCs and polarization of macrophages. Therapeutically, YAP1 attenuation indicates that it enhances the efficacy of immunotherapy.

## Graphical abstract

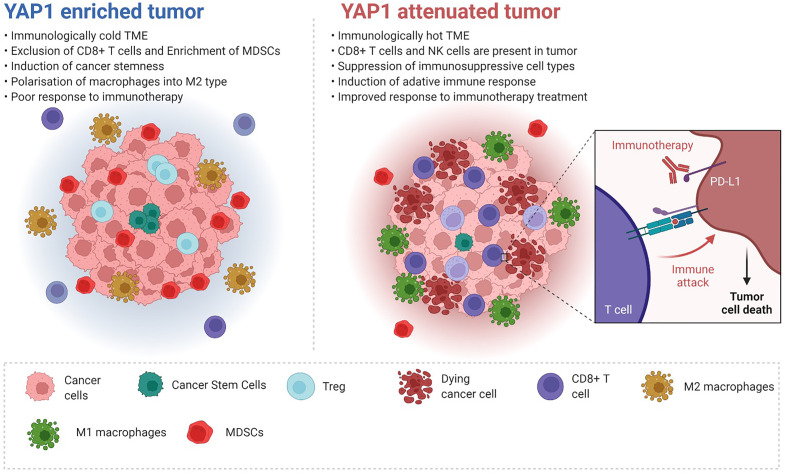

## Introduction

Urothelial carcinoma of the bladder (UCB) is a major cause of mortality among xenobiotic exposure–associated cancers worldwide (1, 2). At present, the molecular mechanisms of UCB development and progression have not been fully elucidated, and it is imperative to study these mechanisms to facilitate the development of better treatment strategies.

In tumorigenesis or immune evasion of a transformed cell, cancer stem cells (CSCs) that regulate pathways (such as the Hippo pathway) play a major role (3–5). We and others previously reported that the Hippo pathway effector YAP1 leads to the generation and expansion of CSCs (6–9). Moreover, YAP1 can potentially regulate the expression of IL-6 and STAT3, two major drivers of immune evasion and generation of CSCs in human cancers (10, 11). While IL-6 stimulates immunity by driving effector T cell expansion and B cell maturation, IL-6/STAT3 has also been identified as a crucial factor in the induction of metastasis, angiogenesis, immunosuppression, macrophage polarization, and myeloid-derived suppressor cell (MDSC) infiltration (12–14). The role of the YAP1-induced IL-6–associated signaling cascade in regulating the tumor microenvironment (TME) has been poorly understood, particularly in UCB. Here, we determined the mechanisms of YAP1-driven immunosuppression, specifically focusing on the IL-6/STAT3 pathway.

MDSCs and macrophages play a major role in the development of an immunosuppressive TME and in driving immune evasion of cancer cells (15). MDSCs are a heterogeneous population of immature myeloid cells that can suppress the activity of cytotoxic CD8^+^ T cells (16). The interplay among different cytokines and chemokines, such as CXCLs, CCLs, and ILs, can significantly regulate the migration and infiltration of MDSCs to the tumor site (17). Activation of oncogenic pathways leads to the expression of CXCLs on the cancer cells that attract the MDSCs in the TME by interacting with the CXCRs expressed on the MDSCs (18). Likewise, depending on the cytokines and chemokines released by cancer cells, infiltrated macrophages in the TME differentiate into either immune-stimulatory M1 or immune-inhibitory M2 macrophages, which have opposite effects on the TME (19). M1 macrophages exhibit proinflammatory and cytotoxic effects, whereas the M2 macrophages have pro-tumorigenic effects (20). Furthermore, macrophages influence several immune components in the TME, such as regulation of T cell differentiation and recruitment, maturation of natural killer cells, and expression of key immune checkpoints such as PD-1, PD-L1, and CTLA4 (21).

YAP1 has been shown to be a potential inducer of lipid droplet (LD) accumulation in cancer cells (22). Notably, a specific component of LDs can induce particular oncogenic cascades in the cancer cells and modify TME (23). Furthermore, recent studies have also identified the critical role of cancer cell–derived extracellular vesicles (EVs) in regulating the tumor immune microenvironment (TIME). Similarly to macrophages, these EVs also have both immune-stimulatory and -suppressive effects (24).

It has been shown that immunotherapy with anti–programmed death (anti-PD) pathway immune checkpoint inhibitors (ICIs) is noticeably less effective in patients with UCB (25) in comparison with other cancers such as melanoma (26, 27). However, the reason for this reduced efficacy is poorly understood. Activation of tumor-specific IL-6, a target of the CSC-regulating pathway molecule YAP1 (7), in the tumor cells induces the expression of PD-L1 (10), and we previously reported that YAP1 regulates CSC generation and expansion in UCB (6). Here, we have also found that YAP1 inhibition increased CD8^+^ T cell infiltration and activity in UCB TIME. Therefore, we hypothesize that a combinatorial therapy targeting CSC-regulating pathway molecules (such as YAP1) and ICI might be an effective strategy to combat immunosuppressive TME and therapeutic resistance to immunotherapy. Our study suggests that YAP1 induces an immunologically “cold” TME, and attenuation of YAP1 enhanced the efficacy of ICI. Overall, this study provides a comprehensive insight into how YAP1 signaling drives cancer stemness and induces an immunosuppressive TME by influencing the infiltration of MDSCs and polarization of macrophages.

## Results

### YAP1 is a potential candidate driver of poor overall survival of patients with UCB and cellular malignant stemness.

**Figure 1:**
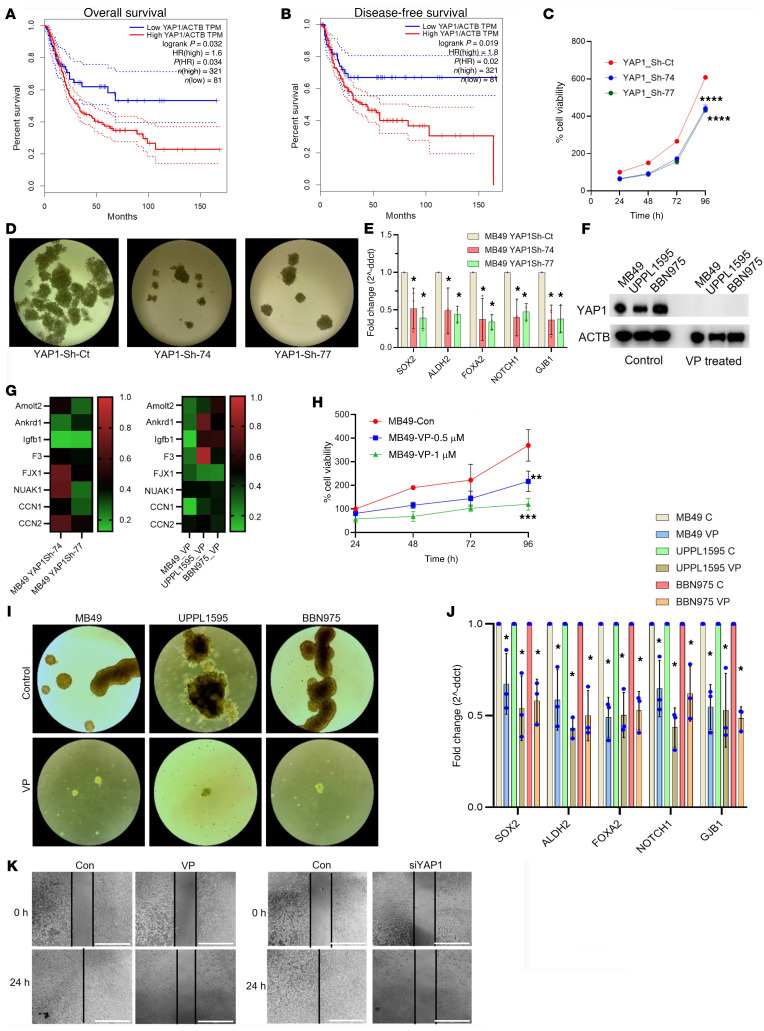
YAP1 is a potential candidate driver of UCB progression and cancer cell malignant stemness. (**A** and **B**) The overall survival (**A**) and disease-free survival (**B**) of patients with a high (top 25%) and a low level (bottom 25%) of YAP1 expression in the TCGA-BLCA database. TPM, transcripts per million. (**C**) Cell proliferation rate in different YAP1 clones. Sh-ct, Sh-control; Sh-74 and Sh-77, YAP1-KD clones. (**D**) Representative images of sphere formation assay of different YAP1-KD and Sh-control of MB49 cells. (**E**) RT-qPCR analysis of candidate cancer stem cell (CSC) markers in YAP1-KD and YAP1 Sh-control MB49 cells. (**F**) Immunoblots showing the YAP1 expression in different mouse parental bladder cancer cell lines and after treatment with 1 μM verteporfin (VP), a potent and specific YAP1 inhibitor. (**G**) RT-qPCR analysis of several YAP1 downstream targets in YAP1-Sh MB49 cells (left) and VP-treated mouse UCB cell lines (right). (**H**) Cell proliferation rate is shown in MB49 cells treated with different concentrations of VP. (**I**) Sphere formation assay of VP-treated (1 μM) mouse bladder cancer cell lines. (**J**) RT-qPCR analysis of the candidate CSC markers using the samples from VP-treated (1 μM) mouse bladder cancer cell lines. (**K**) Representative images of wound healing assay of pharmacological (left) and genetic (right) inhibition of YAP1 in UPPL1595 cells compared with controls. Data are presented as means ± SD of at least 3 independent experiments. *P < 0.05, **P < 0.01, ***P < 0.001, ****P < 0.0001 by unpaired *t* test.

Analysis of The Cancer Genome Atlas (TCGA) database showed significantly worse overall and disease-free survival of patients with UCB with high YAP1 expression (Figure 1, A and B, and Supplemental Figure 1, A and B; supplemental material available online with this article; https://doi.org/10.1172/JCI171164DS1) (28). Moreover, a positive correlation of the expression of YAP1 with known oncogenes (such as TMEM123, DCUN1D5, BRIC2, KRAS, DYCH2H1, PWP1, and RABEPK) was observed (Supplemental Figure 1C). Notably, these latter genes had varied expression levels across UCB subtypes (Supplemental Figure 1D).

To test the hypothesis that YAP1 plays a role in the development of an immunosuppressive TME in UCB, YAP1-knockdown (KD) clones were prepared using a mouse-derived UCB cell line, MB49 (Supplemental Figure 1E). Initial characterization of these KD clones indicated that YAP1 knockdown in the MB49 cells significantly attenuated the cell proliferation rate (Figure 1C). As in our previous studies in human UCB and lung adenocarcinoma (LUAD) cell lines (6, 7), MB49 YAP1-KD clones formed smaller and fewer spheres compared with control cells (Figure 1D). Furthermore, decreased expression of several CSC markers, such as SOX2, ALDH2, FOXA2, NOTCH1, and GJB1, in YAP1-KD MB49 cells suggests that downregulation of YAP1 signaling decreases malignant stemness (Figure 1E). To confirm the phenotypic changes observed due to genetic YAP1 inhibition in MB49 cells, we pharmacologically inhibited endogenously expressed YAP1 in three wild-type mouse UCB cell lines (MB49, UPPL1595, and BBN975) by treatment with a potent and specific YAP1 inhibitor, verteporfin (VP) (1 μM) (Figure 1F and Supplemental Figure 1F), and analyzed downstream targets of YAP1. In general, downstream YAP1 target gene expression was modulated in a similar direction by genetic and pharmacological inhibition of YAP1 (Figure 1, G and H, and Supplemental Figure 1, G and H). As expected, consistent with genetic YAP1 attenuation in MB49 cells, pharmacological inhibition of YAP1 by VP decreased cell proliferation and sphere formation (Figure 1, H and I). RT-qPCR analysis of CSC markers indicated that VP can inhibit the development of cancer stemness in the mouse UCB cell lines (Figure 1J). Furthermore, genetic and pharmacological inhibition of YAP1 activity showed a significant decrease in wound closure in comparison with controls in the UPPL1595 cells (Figure 1K). Overall, these findings indicate the oncogenic potential of YAP1 in selected mouse UCB cell lines, which can be largely reversed through its attenuation.

### Attenuation of YAP1 inhibits tumor growth in vivo.

**Figure 2:**
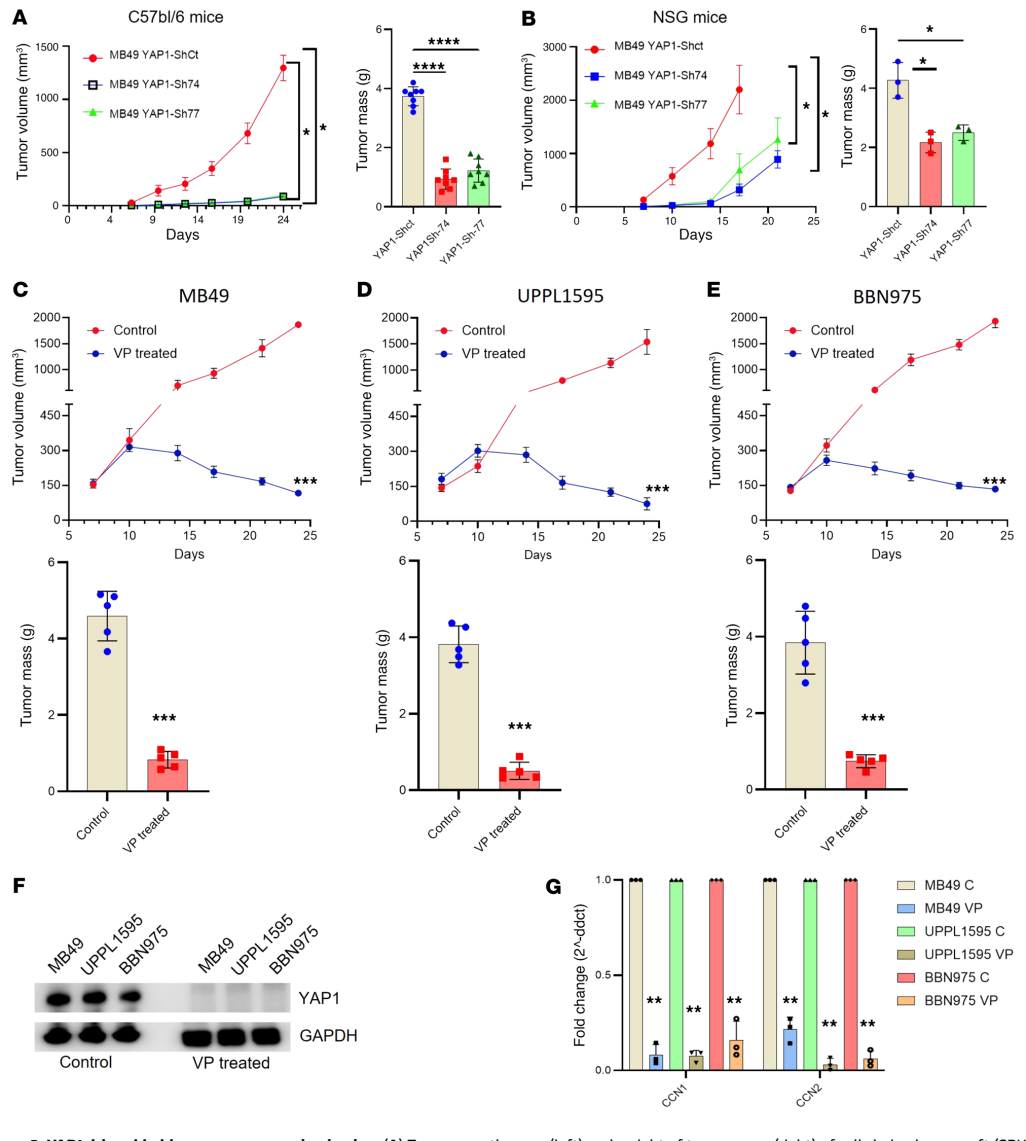
YAP1 drives bladder cancer progression in vivo. (**A**) Tumor growth curve (left) and weight of tumor mass (right) of cell-derived xenograft (CDX) of MB49 YAP1 clones in C57BL/6 mice. (**B**) Tumor growth curve (left) and weight of tumor mass (right) of CDX using MB49 YAP1 clones in immunocompromised NSG mice. (**C**–**E**) Tumor growth curve (top) and tumor mass (bottom) of CDX of MB49, UPPL1595, and BBN975 cells treated with DMSO (control) and YAP1 inhibitor (VP) using C57BL/6 mice. VP was administered 3 times a week, 50 mg/kg body weight. (**F**) Immunoblots showing YAP1 expression level in CDX tissues treated with VP for 25 days (pharmacodynamics of VP). (**G**) RT-qPCR analysis of two YAP1 downstream targets of VP-treated and DMSO-treated mouse xenografted tissues. Data are presented as means ± SD of at least 3 independent experiments. *P < 0.05, **P < 0.01, ***P < 0.001, ****P < 0.0001 by unpaired *t* test.

After confirmation of the oncogenic potential of YAP1 in vitro, cell-derived xenografts (CDXs) were developed in immune-competent mice (C57BL/6) using different YAP1-KD (clones Sh-74 and Sh-77) and Sh-control MB49 cells. Implanted YAP1-KD clones grew at a significantly slower rate compared with the Sh-control clones (Figure 2A). As expected, YAP1-KD tumor weights were significantly less than the weights of the control clones (Figure 2A). To explore whether the impact of YAP1 on tumorigenesis required an intact immune system, a CDX model was developed using the MB49 cell line in immunocompromised NSG mice. Interestingly, for the first 2 weeks after cell implantation, the tumor growth of KD clones was significantly slower in comparison with the control cells (Figure 2B). However, beyond this time point, although the overall tumor volume remained significantly less in the KD cells, the rate of growth of the YAP1-KD tumors rapidly increased when compared with tumor growth in immune-competent mice (Figure 2A). The tumor mass values also showed a significant difference between the KD and control tumors, but the magnitude of the difference was broader in immune-competent mice compared with NSG mice (Figure 2, A and B).

To test the pharmacological inhibition of YAP1 on in vivo tumor growth in immune-competent CD57BL/6 mice, CDXs were developed using 3 different murine cancer cell lines (MB49, UPPL1595, and BBN975). VP was administered intraperitoneally at a dose of 50 mg/kg body weight every other day as we published previously (6). For all 3 cell lines, a significant tumor growth inhibition was observed in response to VP treatment in CD57BL/6 mice (Figure 2, C–E). Tumor mass values also showed a similar trend (Figure 2, C–E). The pharmacodynamic analysis also showed a decrease in YAP1 expression at protein and transcript level in the tumor tissue taken from the VP-treated animals (Figure 2F and Supplemental Figure 1F). To validate the attenuation of YAP1, using the same tumor tissues, RT-qPCR analysis was carried out on expression of CCN1 and CCN2 (YAP1 downstream genes), and the expression of both genes was found to be significantly downregulated upon VP treatment (Figure 2G).

In summary, comparing tumor growth in NSG and C57BL/6 mice, our results indicate that genetic and pharmacological attenuation of YAP1 significantly inhibits the tumor development in immune-competent and NSG mice. However, durable tumor control appears to require an intact immune system.

### YAP1 may enable tumor immune evasion in UCB.

**Figure 3:**
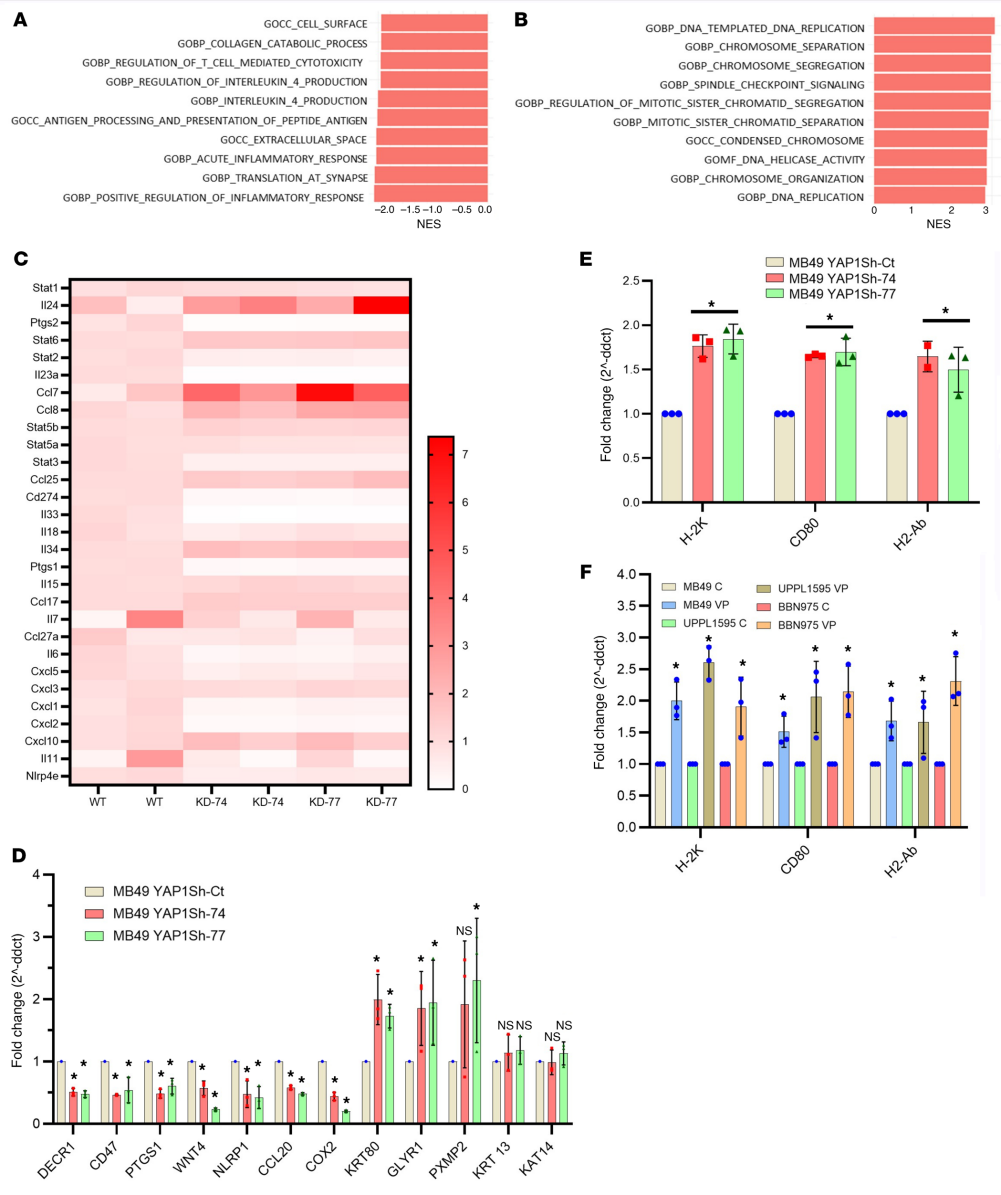
YAP1 might be critical in enabling tumor immune evasion in UCB. (**A**) Fast Gene Set Enrichment Analysis (FGSEA) showed the top 10 downregulated pathways in MB49 YAP1 Sh-Y74 (YAP1-KD) cells compared with MB49 YAP1 Sh-control (Sh-ct; YAP1-expressing) cells. (**B**) FGSEA showed the top 10 upregulated pathways in MB49 YAP1 Sh-Y74 (YAP1-KD) cells compared with MB49 YAP1 Sh-ct (YAP1-expressing) cells. (**C**) Heatmap showing the expression of different key regulatory genes from the interleukin signaling pathway significantly different in MB49 YAP1 Sh-ct cells compared with MB49 YAP1 Sh-Y74 cells. (**D**) RT-qPCR analysis of the candidate immunoregulatory genes in MB49 YAP1-KD and YAP1 Sh-ct clones. (**E**) RT-qPCR analysis of the key immunogenicity markers (H-2K, CD80, and H2-Ab) in YAP1-KD and YAP1 Sh-ct clones. (**F**) RT-qPCR analysis of the key immunogenicity markers (H-2K, CD80, and H2-Ab) in VP-treated mouse UCB cell lines. Data are presented as means ± SD of at least 3 independent experiments. *P < 0.05 by unpaired *t* test.

To investigate the downstream targets in YAP1 signaling–induced tumorigenesis, we performed RNA sequencing (RNA-Seq) of mouse UCB cell lines. Analysis of RNA-Seq data of mouse UCB cell lines with varied levels of YAP1 expression indicated a possible YAP1-driven enrichment of signaling pathways associated with tumorigenesis and tumor immune evasion. For example, Fast Gene Set Enrichment Analysis (FGSEA) of the RNA-Seq data of MB49 cells indicated that YAP1 knockdown leads to downregulation of several immune evasion–associated pathways, including the inflammatory pathway, in MB49 YAP1 Sh-74 (YAP1-KD) cells compared with MB49 YAP1 Sh-control (YAP1-expressing) cells (Figure 3A and Supplemental Figure 2A). FGSEA also indicated that the downregulation of the inflammatory signaling pathway in the YAP1-expressing cell line correlated with upregulation of cell cycle–regulatory genes in the MB49 YAP1 Sh-74 clones (Figure 3B and Supplemental Figure 2B). Analysis of the RNA-Seq data from MB49 cells indicated a similar trend of downregulated WNT/β-catenin and EMT signaling that also indicates the oncogenic potential of YAP1 (Supplemental Figure 2, C and D). A similar trend in downregulation of oncogenic pathways was observed in a human UCB cell line (UC3) upon YAP1 downregulation (Gene Expression Omnibus [GEO] GSE186043) (Supplemental Figure 2, E and F) (29). In our RNA-Seq data of MB49 cells, we found that several genes (such as IL6, STAT3, and CXCLs) of the interleukin pathway were significantly altered in the MB49 cells upon YAP1 downregulation (Figure 3C). For validation, we performed RT-qPCR of several candidate genes known to regulate the TME and found that genes that promote tumorigenesis and immune evasion were significantly downregulated in YAP1-Sh clones (Figure 3D). Furthermore, by RT-qPCR analysis we found that genetic and pharmacological attenuation of YAP1 upregulated the expression of MHC markers such as H-2K, H2-Ab, and costimulatory molecules such as CD80 (Figure 3, D–F). In summary, the analysis of RNA-Seq data generated from the mouse YAP1-Sh MB49 cell line and publicly available RNA-Seq data from a human UCB cell line (UC3, GSE186043) led us to hypothesize that YAP1 may play a significant role in tumor immune evasion.

### YAP1 facilitates an immunosuppressive TME.

**Figure 4:**
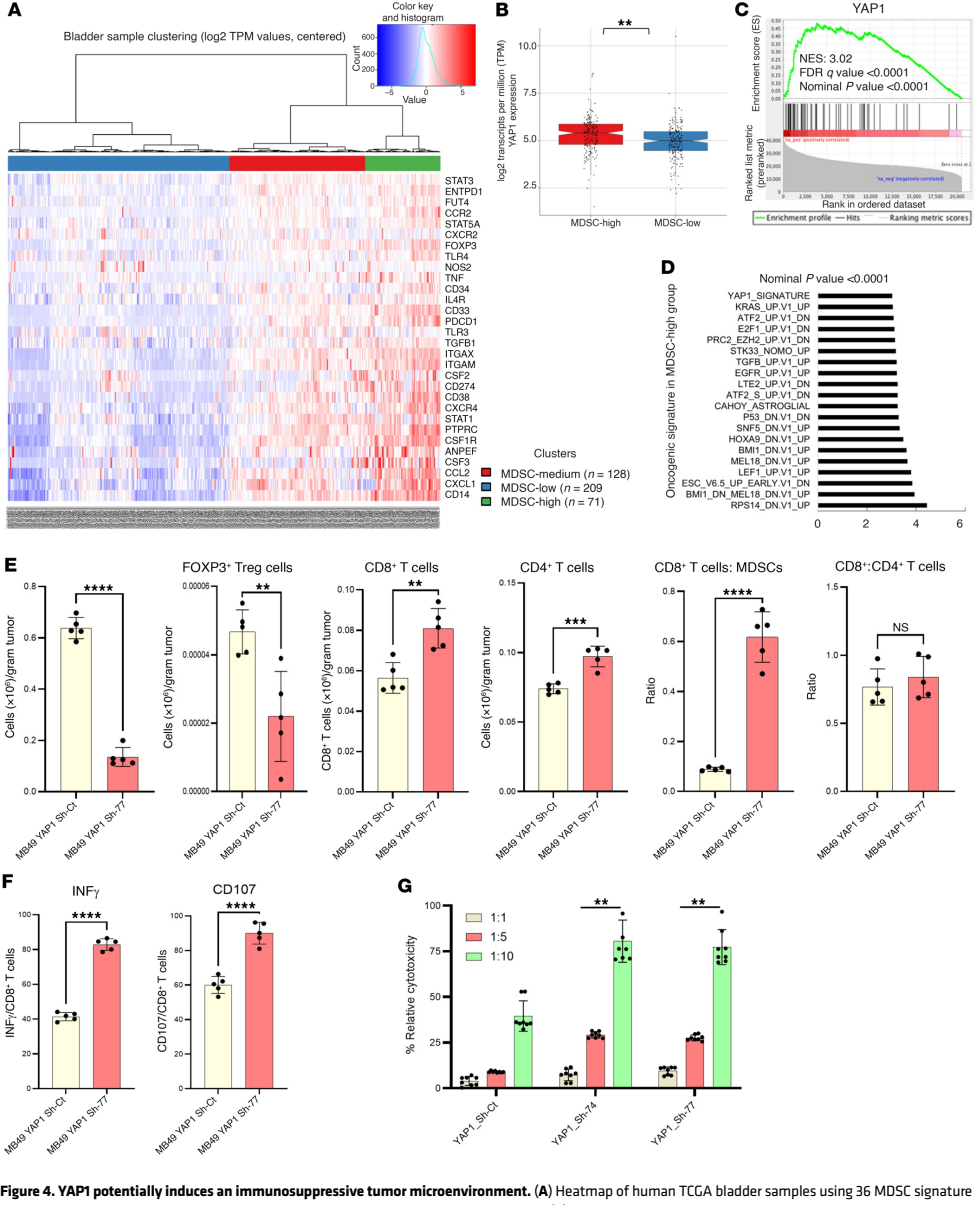
YAP1 potentially induces an immunosuppressive tumor microenvironment. (**A**) Heatmap of human TCGA bladder samples using 36 MDSC signature genes. Samples were clustered into 3 groups: MDSC-high, MDSC-low, and MDSC-medium. (**B**) Expression of YAP1 in MDSC-high and MDSC-low groups of tumors analyzed from TCGA bladder samples. (**C** and **D**) Gene set enrichment analysis (GSEA) showed enrichment of YAP1 signature genes in MDSC-high bladder TCGA samples. (**E**) Flow cytometric analysis showing the infiltration of MDSCs, FOXP3^+^ Tregs, CD8^+^ T cells, and CD4^+^ T cells in xenografted tumors. (**F**) Flow cytometric analysis showing the expression of CD107 and IFN-γ in CDX tissues. *n* = 5 in each group. (**G**) Coculture cytotoxicity assay of CD8^+^ T cells and cancer cells measured by quantification of the released lactate dehydrogenase in the culture media. Data are presented as means ± SD of at least 3 independent experiments. **P < 0.01, ***P < 0.001, ****P < 0.0001 by unpaired *t* test.

Analysis of the TCGA database indicates that high YAP1 expression is associated with an enriched signature of MDSCs in UCB (Figure 4A). YAP1 expression itself was also found to be higher in high MDSC-infiltrated UCB samples (Figure 4B). Furthermore, the gene set enrichment analysis (GSEA) revealed that high presence of MDSCs in the tumor tissue resulted in upregulation of different oncogenic pathways compared with low-MDSC tumor tissue (Figure 4C). Moreover, YAP1 was one of the most upregulated gene signatures in high-MDSC UCB samples (Figure 4D). These analyses of our RNA-Seq data (Figure 3A and Supplemental Figure 2, A and B) and 2 publicly available UCB databases (TCGA-BLCA and GSE186043) (Figure 4, A–D, and Supplemental Figure 2, C–F) indicate that YAP1 is closely associated with several immune-regulating pathways. Therefore, we speculated that YAP1 might have a major role in the regulation of the TIME. To further understand the role of YAP1 in immune regulation of the TME and for experimental validation, we developed CDX models in C57BL/6 mice using MB49 YAP1 Sh-control and MB49 YAP1-KD clone. The numbers of MDSCs and FOXP3^+^ T cells were found to be significantly reduced in YAP1-KD tumors compared with Sh-control tumors (Figure 4E). Additionally, increased infiltration of CD8^+^ T and CD4^+^ T cells was observed in the YAP1-Sh tumors (Figure 4E). The ratio of CD8^+^ T cells to MDSCs was higher in YAP1-Sh tumors (Figure 4E). The ratio of CD8^+^ T cells to CD4^+^ T cells was higher in YAP1-Sh tumors (Figure 4E). Moreover, expression analysis of the T cell activation markers CD107 and IFN-γ indicated greater T cell activation in the YAP1-attenuated tumor tissues compared with controls (Figure 4F). For further validation, we performed immunohistochemical (IHC) staining of Gr-1 and CD8 using the tumor tissue derived from YAP1-expressing and YAP1-downregulated MB49 cells, and our findings were consistent with flow cytometric analysis (Supplemental Figure 2G): decreased numbers of MDSCs (Gr-1) and increased numbers of CD8^+^ T cells in YAP1-Sh tumors. Treatment of mice with YAP1 inhibitor in YAP1-expressing MB49 xenografted tumors also showed a similar pattern of MDSC and CD8 cell infiltration (Supplemental Figure 2H). These findings clearly indicate that YAP1 attenuation increases the infiltration of CD8^+^ cells and decreases the infiltration of MDSCs in the TME. Simultaneously, YAP1 attenuation was associated with increased CD8^+^ T cell cytotoxicity (Figure 4G). To explore whether MDSCs are one of the critical immune factors driven by YAP1 in UCB tumorigenesis, a separate CDX model was developed with wild-type (WT) MB49 cells. An anti-Ly6G antibody (anti-MDSC) was administered into animals every day for 3 weeks. Tumor progression rate and tumor mass indicate that the anti-Ly6G antibody significantly decreased tumor growth in comparison with the controls (animals given IgG) (Supplemental Figure 2I). Overall, our findings indicate that YAP1 is one of the critical factors in inducing an immunosuppressive TME by facilitating the infiltration of MDSCs while reducing the infiltration and cytotoxic activity of T cells.

### YAP1 influences MDSC migration and macrophage migration and polarization.

**Figure 5:**
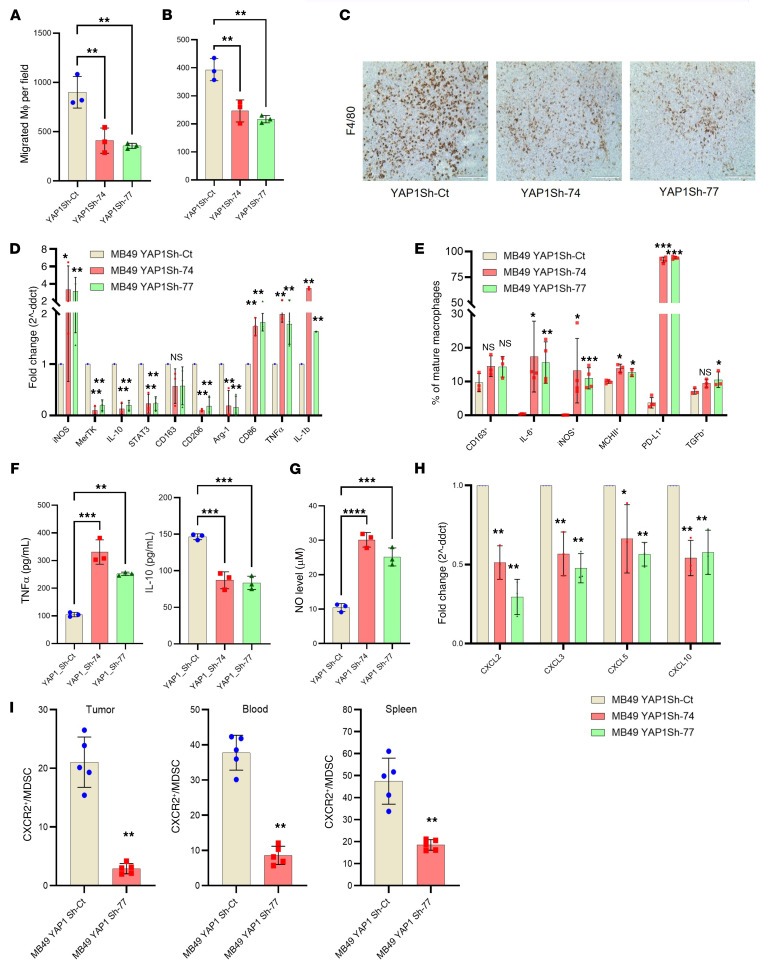
YAP1 potentially modulates the activity of MDSCs and macrophages in the xenograft tumor. (**A**) Migration assay using primary macrophages from the peritoneum of WT C57BL/6 mice and conditioned medium (CM) from in vitro–cultured MB49 YAP1-Sh and YAP1-Sh clones. (**B**) Migration assay using MDSCs from WT MB49 xenografts and CM from in vitro–cultured MB49 YAP1-Sh and YAP1-Sh clones. (**C**) Representative IHC images showing the presence of macrophages (F4/80^+^) in xenografts developed from MB49 YAP1 Sh-control (Sh-ct) and YAP1-Sh clones (*n* = 3). Scale bar: 200 μm. (**D**) RT-qPCR analysis of macrophage polarization markers in RAW 264.7 cell line cultured with CM from YAP1 Sh-ct and YAP1-Sh clones. (**E**) Flow cytometric analysis showing the expression of candidate macrophage polarization markers in RAW 264.7 cell line cultured with CM from MB49 YAP1-Sh and Sh-ct clones. (**F**) ELISA showing the level of 2 cytokines (IL-10 and TNF-α) released from macrophages incubated with the CM of YAP1-Sh clones. (**G**) Griess assay showing the level of nitric oxide (NO) in the culture medium of macrophages incubated with the CM of YAP1-Sh and Sh-ct clones. (**H**) RT-qPCR assay showing the expression level of CXCR2-associated ligands in YAP1-KD MB49 cells. (**I**) FACS analysis showing CXCR2 expression level in the tumor, blood, and spleen of MB49 YAP1-Sh and Sh-ct clones bearing xenografts. Data are presented as means ± SD of at least 3 independent experiments. *P < 0.05, **P < 0.01, ***P < 0.001, ****P < 0.0001 by unpaired *t* test.

The TME drives numerous phenotypic changes associated with tumor initiation and progression, including metastasis, angiogenesis, cancer stemness, and immune evasion (30, 31). To get further insight into the role of YAP1 in the TME and to understand the influence of YAP1 expression on the infiltration of MDSCs and macrophages, MDSCs were isolated from tumor mass developed from MB49 YAP1-Sh and Sh-control clones and primary macrophages were isolated from the intraperitoneal cavity of WT mice. We then performed migration assays using these isolated MDSCs and macrophages, and our results indicate that conditioned medium (CM) from YAP1 Sh-control cells attracted more macrophages (Figure 5A) and MDSCs (Figure 5B) compared with CM from YAP1-Sh clones. IHC micrographs also indicated less infiltration of macrophages (F4/80^+^) in the TME of YAP1-attenuated tumors compared with Sh-controls (Figure 5C).

To determine the role of YAP1 on macrophage polarization, we performed RT-qPCR and FACS analysis of selected macrophage polarization markers in macrophages cultured with YAP1 control or YAP1-Sh CM. We found decreased expression of CD206, MerTK, IL-10, Arg-1, and STAT3 (M2 phenotype markers) and increased expression of iNOS, IL-6, and MHCII (M1 phenotype markers) in macrophages cultured with CM of YAP1-Sh clones compared with CM of Sh-control MB49 clones (Figure 5, D and E). To solidify RT-qPCR findings, we performed ELISA for 2 key M1/M2 factors (TNF-α and IL-10, respectively) using CM of YAP1-Sh and Sh-control clones, and the findings are consistent with our RT-qPCR data (Figure 5F). Furthermore, nitric oxide production (a characteristic of M1 macrophages) was increased in macrophages cultured with CM from YAP1-Sh MB49 cells as determined by the Griess assay (Figure 5G). External validation was performed for M2 macrophage markers by analysis of the publicly available IMvigor210 clinical cohort (32). Our analysis of this cohort indicated that M2 markers have lower expression in the immunotherapy-responsive group (Supplemental Figure 3, A and B). In summary, our findings indicate that YAP1 drives an immunosuppressive TME with infiltration of M2 macrophages and MDSCs, while YAP1 attenuation may polarize macrophages in a shift to the M1 phenotype. These M1 macrophages can be beneficial in inhibiting tumor progression and inducing an immunologically “hot” TME.

### Genetic knockdown of YAP1 leads to deregulation of immune-associated cytokines/chemokines.

Analyses of the TCGA-BLCA database revealed that CXCL10 is one of the top 25 upregulated genes in UCB (Supplemental Figure 4A). To further understand the mechanism of YAP1-associated TIME, we analyzed a panel of 32 cancer-associated cytokines/chemokines on a qPCR array in YAP1-Sh and YAP1 Sh-control MB49 cells and CDXs. The array data revealed that YAP1-expressing MB49 cells (Supplemental Figure 4B) and xenografts (Supplemental Figure 4C) expressed increased CXCR2-associated ligands, such as CXCL2, CXCL3, and CXCL5 (Supplemental Figure 4, B and C). By RT-qPCR, we revalidated the expression of CXCLs (CXCL2, CXCL3, CXCL5, CXCL10) in MB49 YAP1-Sh clones and tumor tissues from VP-treated mice bearing UCB cell–derived xenografts (Figure 5H and Supplemental Figure 4D). We also analyzed the expression of CXCR2 in xenografts as well as in the blood and spleen of the tumor-bearing animals. Our findings revealed that CXCR2 expression decreased in MDSCs from tumors, blood, and spleen collected from YAP1-Sh xenografts (Figure 5I).

To determine YAP1-associated regulation of chemokines noted above in human UCB, we first performed RT-qPCR analysis of CXCR2-associated ligands (CXCL2, CXCL3, and CXCL6) in human cancer cell lines (T24, BFTC905, BFTC909, and UMUC3) with YAP1 modulation. Consistent with our mouse data, these 3 chemokines were downregulated in YAP1-Sh human cells (BFTC905 and T24) while upregulation was observed in YAP1-overexpressed human cells (BFTC909 and UMUC3) (Supplemental Figure 4, E–H). We further analyzed the expressions of CXCL2, CXCL3, CXCL6, and YAP1 in a human primary UCB cohort (*n* = 30) using RT-qPCR and found a significant correlation of YAP1 expression with these cytokines (Supplemental Figure 4I). External validation using the TCGA-BLCA cohort generated similar findings (Supplemental Figure 4J). Collectively, our results indicate that YAP1 induction led to the expression of various cancer-promoting chemokines/cytokines and these in turn led to induction of an immunosuppressive TME.

### YAP1 activates the IL-6/STAT3 pathway during UCB progression.

**Figure 6:**
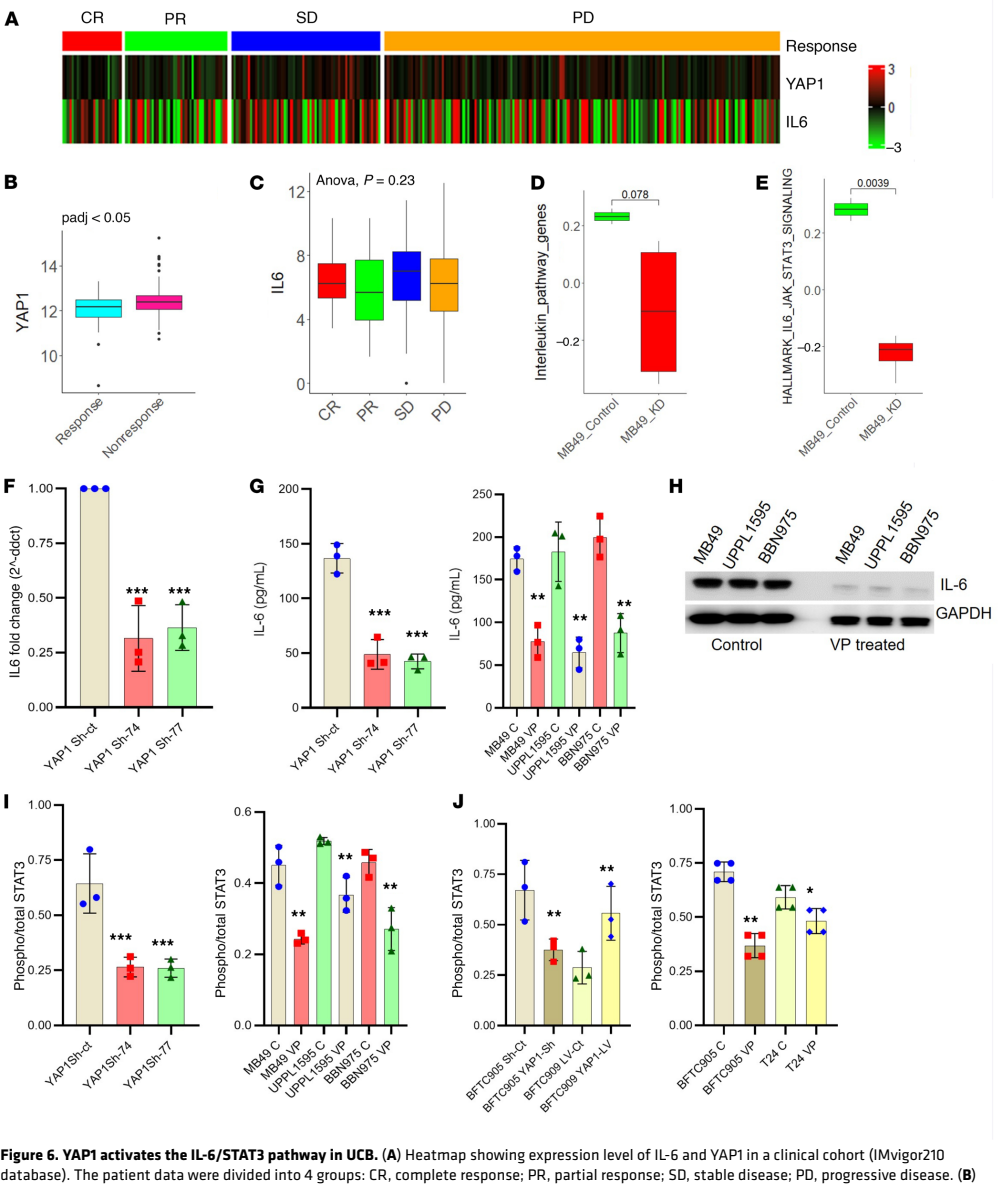
YAP1 activates the IL-6/STAT3 pathway in UCB. (**A**) Heatmap showing expression level of IL-6 and YAP1 in a clinical cohort (IMvigor210 database). The patient data were divided into 4 groups: CR, complete response; PR, partial response; SD, stable disease; PD, progressive disease. (**B**) YAP1 expression was significantly high in immunotherapy-nonresponsive group (SD and PD) compared with responsive group (CR and PR) in IMvigor210 clinical cohort (*P* < 0.05 by *t* test). (**C**) Expression level of IL-6 in different subgroups (CR, PR, SD, and PD) of immunotherapy-treated IMvigor210 clinical cohort (ANOVA, *P* = 0.23). (**D** and **E**) RNA-Seq data showing downregulation of the key regulatory genes of interleukin signaling and IL-6/STAT3 signaling in MB49 YAP1-Sh cells. (**F**) RT-qPCR analysis of IL-6 expression in MB49 YAP1-Sh and Sh-control (Sh-ct) clones. (**G**) ELISA for IL-6 expression in MB49 YAP1-Sh and Sh-ct clones (left) and VP-treated mouse UCB cell lines (right). (**H**) Immunoblot showing the expression of IL-6 in VP-treated mice bearing CDX from WT MB49, UPPL1595, and BBN975 cells. (**I**) ELISA showing the phospho-STAT3/total STAT3 expression ratio in MB49 YAP1-KD and Sh-ct clones (left) and in VP-treated WT mouse UCB cell lines (right). (**J**) ELISA showing phospho-STAT3/total STAT3 in YAP1-Sh (BFTC905) and YAP1-overexpressed (lentiviral vector [LV]) (BFTC909) human UCB cell lines (left), and ELISA showing phospho-STAT3/total STAT3 in VP-treated human UCB cells (right). Data are presented as means ± SD of at least 3 independent experiments. *P < 0.05, **P < 0.01, ***P < 0.001 by unpaired *t* test.

Using LUAD cell lines, we previously showed that YAP1 binds to the promoter region of IL-6 and induces its transcription to result in upregulation of the phosphorylation of STAT3 (active form) (7). This observation led us to investigate IL-6 expression in UCB patient samples and cell lines in context with YAP1 expression. Analysis of the publicly available IMvigor210 database (32) revealed that YAP1 expression is noticeably upregulated in immunotherapy-nonresponsive patients (Figure 6, A and B). Interestingly, upregulation of IL-6 was also observed among the partial response (PR) and stable disease (SD) groups (Figure 6C), indicating that IL-6 expression may also contribute to the developing resistance to ICIs. Furthermore, our analysis of the IMvigor210 database showed a decreased trend of overall survival of patients who expressed YAP1 and IL-6, IL-6, and STAT3 (Supplemental Figure 5, A–C). Additionally, analysis of the TCGA-BLCA database showed that both IL-6 and STAT3 expressions are positively correlated with the expression of YAP1 (Supplemental Figure 5, D and E). The RNA-Seq data from the MB49 YAP1-Sh clones also showed a downregulation of key interleukin pathway genes and IL-6/STAT3 signaling (Figure 6, D and E).

The above in silico analysis prompted us to explore the correlation of YAP1 with IL-6/STAT3 signaling in UCB. Our results indicate that genetic and pharmacological attenuation of YAP1 led to the downregulation of IL-6 at RNA and protein levels (Figure 6, F–H, and Supplemental Figure 5F). As STAT3 is a target of IL-6 (11, 33), we validated the phosphorylation status of STAT3 in UCB cell lines. An ELISA with intracellular protein showed that STAT3 phosphorylation was positively correlated with the expression of YAP1 (Figure 6I). Similar results were also observed after pharmacological and genetic inhibition of YAP1 in human UCB cell lines (Figure 6J).

### YAP1 induces immunosuppression partially through IL-6/STAT3 signaling.

**Figure 7:**
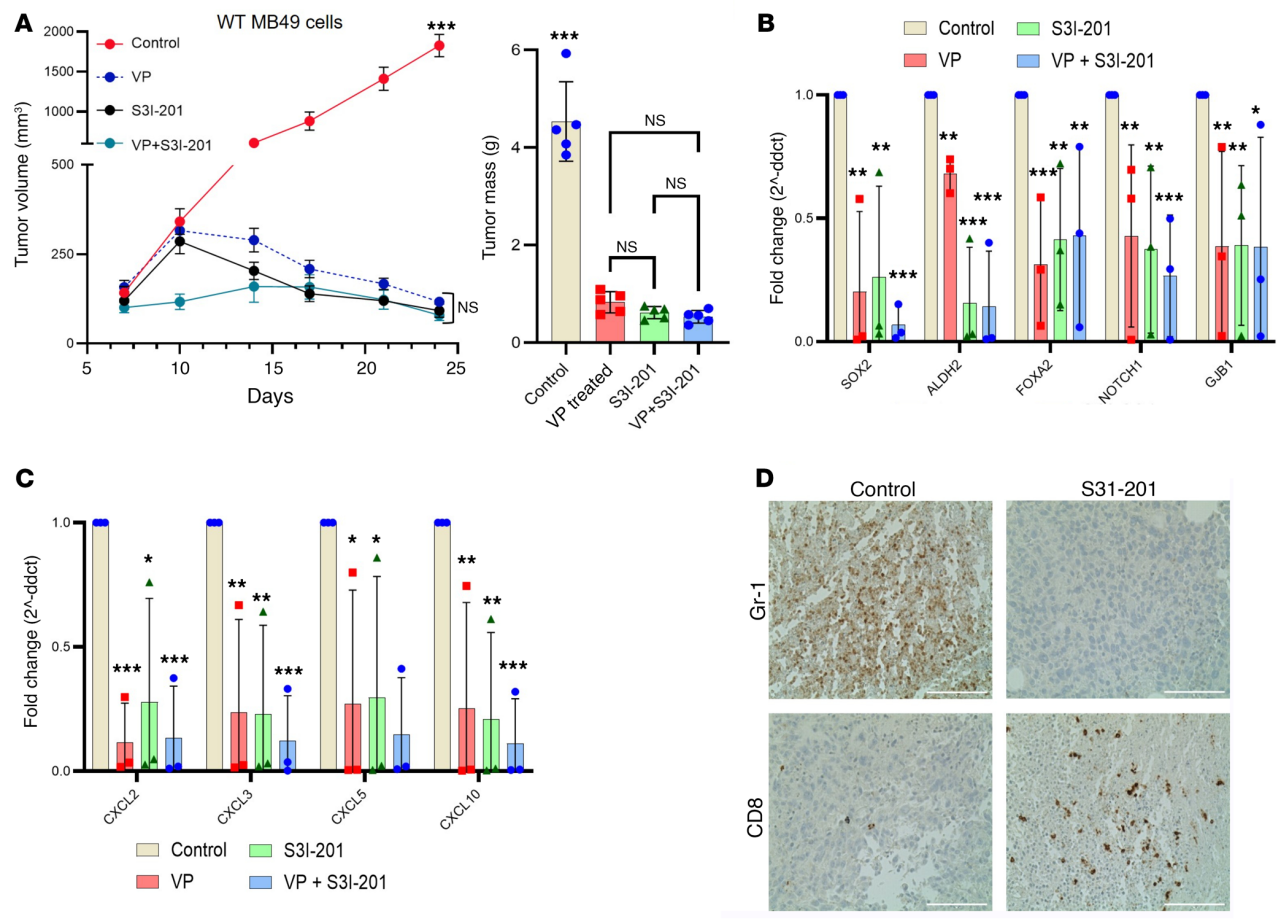
STAT3 inhibition mimics the antitumor activity of YAP1 attenuation. (**A**) MB49 WT cell–derived tumors in C57BL/6 mice treated with STAT3 inhibitor (S3I-201), YAP1 inhibitor (VP), and combination of S31-201 and VP. Left: Growth curve. Right: Tumor mass. (**B**) RT-qPCR analysis showing the expression of different CSC markers in MB49 WT xenografts collected from drug- and vehicle-treated mice. (**C**) RT-qPCR analysis showing the expression of different CXCR1/CXCR2-associated ligands in the MB49 WT xenografts collected from drug- and vehicle-treated mice. (**D**) IHC showing the infiltration of MDSCs (Gr-1) and CD8^+^ T cells in the tumor site of S3I-201–treated and control mouse tumor. Scale bar: 100 μm. Data are presented as means ± SD of at least 3 independent experiments. *P < 0.05, **P < 0.01, ***P < 0.001 by 1-way ANOVA.

We recently reported that YAP1 positively regulates IL-6/STAT3 signaling in LUAD (7). Different studies suggest that CXCLs are critical players in inducing an immunosuppressive TME through the infiltration of MDSCs in tumor sites and cytotoxic T cell exhaustion (31, 34, 35). Here, in our study we found that YAP1 influenced CXCL expression (Figure 5H) and induction of phosphorylated STAT3 (Figure 6I). To further explore the YAP1/IL-6/STAT3/CXCL signaling axis in UCB, we treated YAP1-expressing MB49-derived xenograft–bearing C57BL/6 mice with S3I-201 (STAT3 inhibitor). Our results indicated significant tumor growth inhibition with S31-201 treatment (Figure 7A). We found no significant difference among the animals treated with only VP, only S3I-201, and combination of VP and S3I-201. The RT-qPCR analysis showed significantly decreased expression of CSC-associated markers and CXCL genes in tumor tissues of the STAT3 inhibitor–treated group (Figure 7, B and C), which appeared similar to YAP1 attenuation in these cell lines (Figures 1 and 5). Overall, there was no therapeutic advantage of using combination of YAP1 inhibitor and STAT3 inhibitor. Notably, the IHC analysis revealed that STAT3 inhibition led to decreased infiltration of MDSCs (Gr-1) and noticeably more CD8^+^ T cells in the TME (Figure 7D), which is also in agreement with YAP1 inhibition (Figure 4E and Supplemental Figure 2, G and H). These data support the notion that YAP1 plays a role in inducing immunosuppression through IL-6/STAT3 signaling.

### YAP1 influences the accumulation of lipid droplets in cancer cells.

**Figure 8:**
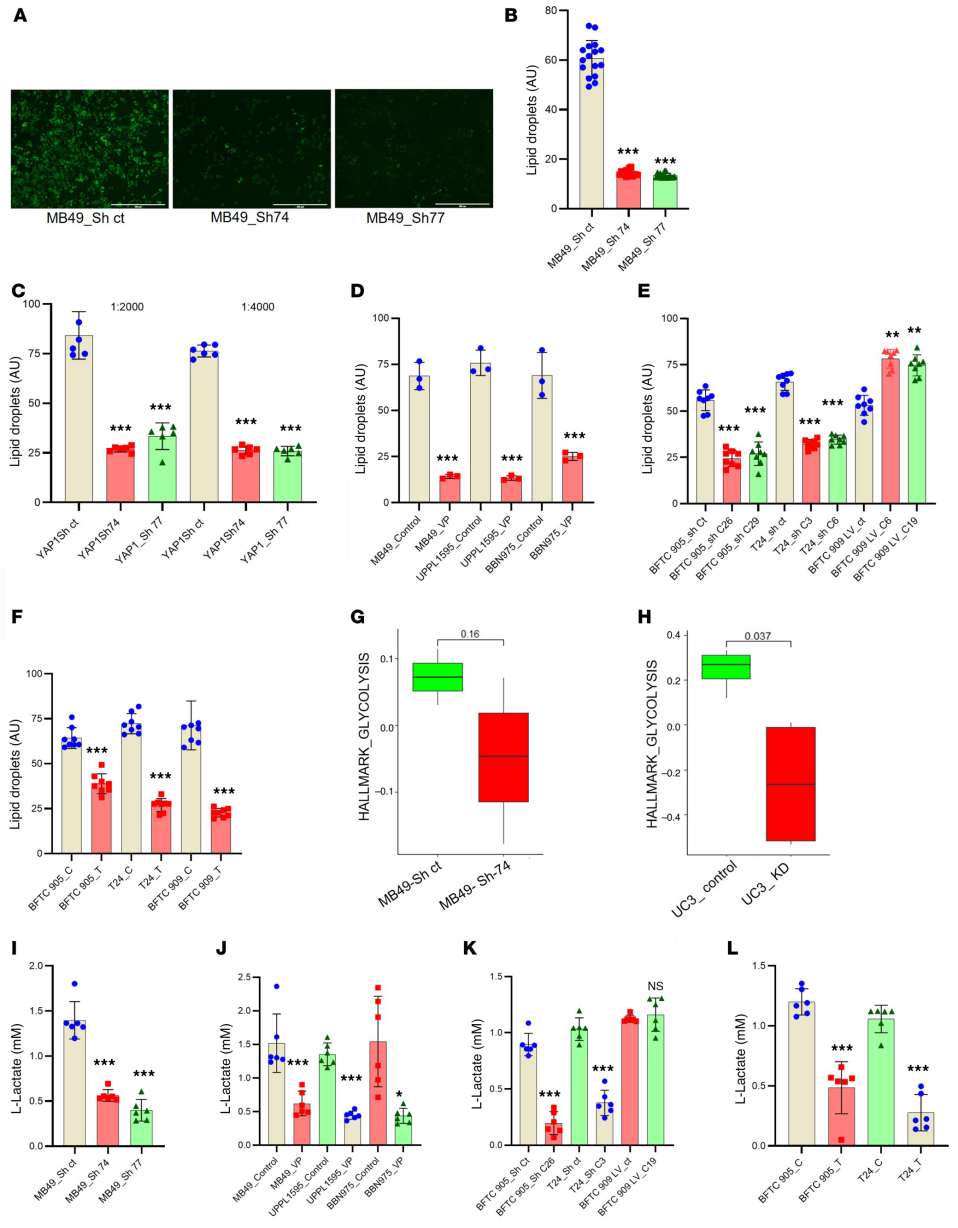
YAP1 influences the accumulation of lipid droplets in cancer cells. (**A**) Micrographs (original magnification, ×10) showing the lipid droplets (LDs) in MB49 YAP1-Sh and Sh-control (Sh-ct) clones. Scale bar: 400 μm. (**B**) Quantification of LD accumulation in MB49 YAP1-Sh clones by fluorescent spectroscopy. (**C**) Quantification of LD accumulation in MB49 YAP1-Sh clones exposed to exogenous oleic acid. (**D**) Quantification of LD accumulation in VP-treated WT mouse UCB cell lines. (**E**) Quantification of LD accumulation in YAP1-Sh (BFTC905 and T24) and YAP1-overexpressed (LV) (BFTC909) human UCB cell lines. (**F**) Quantification of LD accumulation in WT human UCB cell lines. C, control; T, treated. (**G** and **H**) RNA-Seq data from MB49 (**G**) and UC3 (**H**) YAP1-KD cells showing downregulation of the glycolytic pathway–regulatory genes. (**I**) Quantification of l-lactate in MB49 YAP1-Sh clones (Sh-74 and Sh-77) and Sh-ct. (**J**) Quantification of l-lactate in VP-treated WT mouse UCB cell lines. (**K**) Quantification of l-lactate in YAP1-Sh (BFTC905 and T24) and YAP1-overexpressed (LV) (BFTC909) human UCB cell lines. (**L**) Quantification of l-lactate in WT human UCB cell lines. C, control; T, treated. AU, arbitrary units. Data are presented as means ± SD of at least 3 independent experiments. *P < 0.05, **P < 0.01, ***P < 0.001 by unpaired *t* test.

Our RNA-Seq data indicate that fatty acid–binding protein 4 (FABP4) is one of the top upregulated targets in YAP1-expressed cells. As reported (36), FABP4 is required for accumulation of lipid droplets (LDs) in the intracellular compartments, and LD was reported to ease immune evasion, cancer malignant stemness, and poor prognosis of cancer patients (37, 38). Therefore, we validated FABP4 by RT-qPCR from different MB49 YAP1 clones and found that FABP4 expression directly correlated with YAP1 expression (Supplemental Figure 6A). Pharmacologically, the expression level of FABP4 was significantly downregulated after treatment of MB49, UPPL1595, and BBN975 cell lines with the YAP1 inhibitor (Supplemental Figure 6B). Analysis of the publicly available database IMvigor210 also indicated that genes responsible for lipid storage have an inverse correlation with the immunotherapy response (Supplemental Figure 6C). RNA-Seq data from human UC3 cells (GSE186043) also suggest significant downregulation of key regulatory genes of fatty acid metabolism by YAP1 knockdown (Supplemental Figure 6D). Our data also revealed that YAP1 downregulation in MB49 cells decreased the accumulation of LDs (Figure 8, A and B). We also observed that when the culture medium was supplemented with oleic acid (commonly used as an inducer for LD formation), there was an increased accumulation of LDs in the YAP1-expressing cells compared with the YAP1-Sh MB49 cells (Figure 8C). Similar results were observed in the VP-treated mouse xenografts of UCB cell lines (MB49, UPPL1595, BBN975) (Figure 8D). We also found that the attenuation of YAP1 in human UCB cell lines decreased the accumulation of intracellular LDs (Figure 8, E and F). Consistent with decreased LDs in YAP1-Sh cells, RNA-Seq data from mouse MB49 YAP1-Sh and human UC3 YAP1-KD cells (GSE186043) showed YAP1 downregulation led to downregulation of glycolysis-regulatory genes (Figure 8, G and H). Furthermore, YAP1 attenuation in mouse (Figure 8, I and J) and human UCB cell lines (Figure 8, K and L) showed decreased levels of l-lactate.

### YAP1 deregulation modulates host adaptive immunity by influencing the secretion of regulatory cytokines and chemokines in the TME.

**Figure 9:**
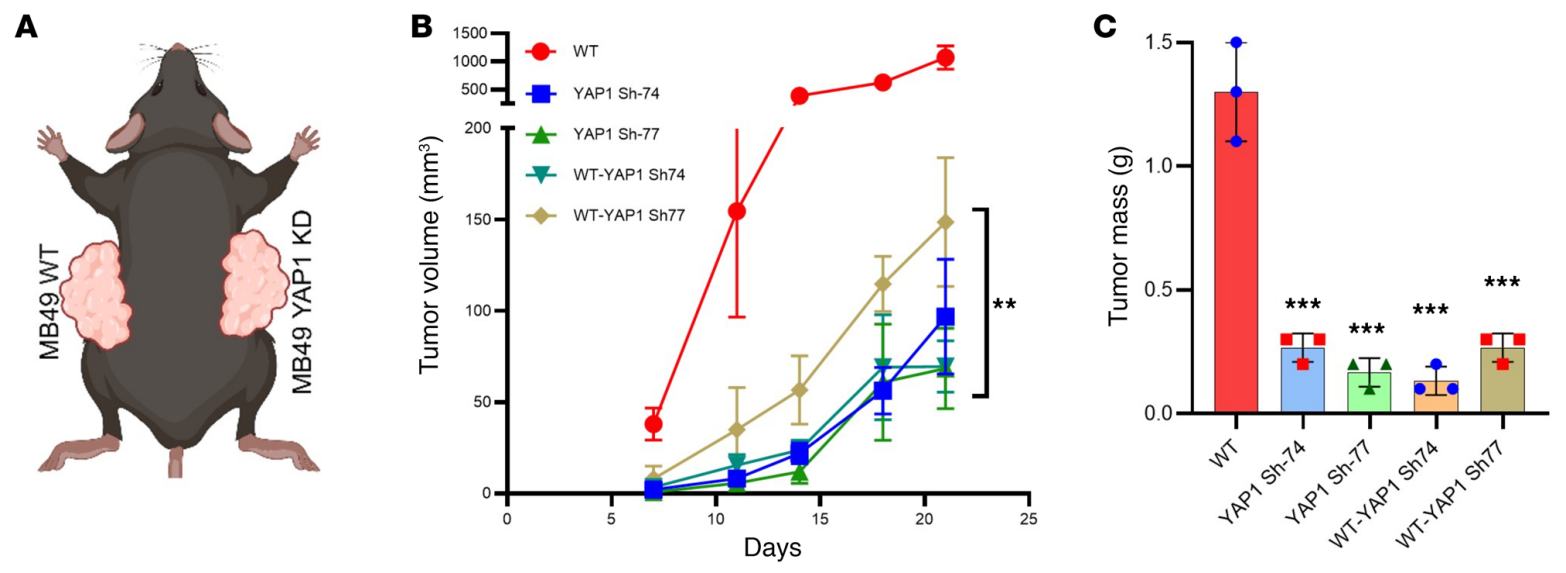
YAP1 silencing in tumors stimulates host adaptive immunity. (**A**) A schematic showing the cell injection scheme in the mice. YAP1-expressing MB49 cells were injected in one flank and MB49 YAP1-Sh clones were injected in the opposite flank. (**B**) Tumor growth curve of WT MB49 or MB49 YAP1-Sh clones. WT: mice were injected with WT cells in both flanks; YAP1 Sh-74: mice were injected with MB49 YAP1 Sh-Y74 cells in both flanks; YAP1 Sh-77: mice were injected with MB49 YAP1 Sh-Y77 cells in both flanks; WT-YAP1 Sh-74: mice were injected with WT cells in the left flank and MB49 YAP1 Sh-Y74 cells in the right flank; WT-YAP1 Sh-77: mice were injected with WT cells in the left flank and MB49 YAP1 Sh-Y77 cells in the right flank. (**C**) Tumor mass. Data are presented as means ± SD of at least 3 independent experiments. **P < 0.01, ***P < 0.001 by 1-way ANOVA.

Our results described above indicate that YAP1 expression induces an immunosuppressive TME. Therefore, to test the hypothesis that inhibiting YAP1 will promote the host adaptive immune response, an animal model was developed by simultaneous injection of YAP1-Sh MB49 clones and YAP1-expressing WT MB49 cells into the opposite flank of the same C57BL/6 mouse (Figure 9A). Interestingly, YAP1-expressing WT MB49 tumors had reduced size in comparison with the YAP1-Sh clone in dually implanted mice when compared with tumors isolated from mice injected with YAP1-expressing WT MB49 cells alone (Figure 9B and Supplemental Figure 7A). The tumor growth curve, tumor mass, and gross images of isolated tumors clearly indicate that when WT MB49 cells were injected in the opposite flank of the YAP1-Sh clone (Sh-74 and Sh-77) site, the growth rate and tumor development were significantly attenuated (Figure 9, B and C). IHC analyses revealed a decreased infiltration of MDSCs in tumors from YAP1-Sh and WT MB49 grown in the same mice compared with WT MB49 tumors grown in a separate mouse (Supplemental Figure 7B). Interestingly, CD8^+^ T cells were noticeably increased in WT MB49 tumors grown in the same mice with YAP1-Sh tumors compared with WT MB49 tumors grown in separate mice (Supplemental Figure 7B). Notably, IL-6, a critical factor in tumorigenesis and immune evasion, showed reduced expression in the WT MB49 tumors grown in the opposite flank with YAP1-Sh tumors compared with WT MB49 tumors grown in a separate mouse (Supplemental Figure 7C). These results indicate that YAP1 expression may regulate critical factors (regulatory cytokines and chemokines) that induce an immunosuppressive TME in vivo. Recently, numerous studies have suggested that extracellular vesicles (EVs) are one of the major sources of these secretory molecules (24). Our findings suggested that the number of EVs from the YAP1-Sh cells was higher in comparison with control cells (Supplemental Figure 7D). Protein quantification analysis also revealed that EVs isolated from the YAP1-Sh clones had significantly higher protein content in comparison with the control MB49 YAP1 Sh-control cells (Supplemental Figure 7E). To explore the functional role of YAP1-associated EVs, we treated naive macrophages with EVs isolated from YAP1-Sh and YAP1 WT MB49 cells. Our findings revealed that EVs from YAP1-Sh clones induced expression of M1 phenotype markers (CD86, IL-1b, TNF) but reduced expression of M2 phenotype markers (CD206, CD163, Arg-1) in the naive macrophages (Supplemental Figure 7F). Although further studies are needed, these findings indicate that the YAP1-regulated secreted EVs might play a role in polarizing macrophages and influence the development of adaptive antitumor immunity.

### YAP1 inhibition in combination with anti–PD-L1 shows synergistic antitumor efficacy.

**Figure 10:**
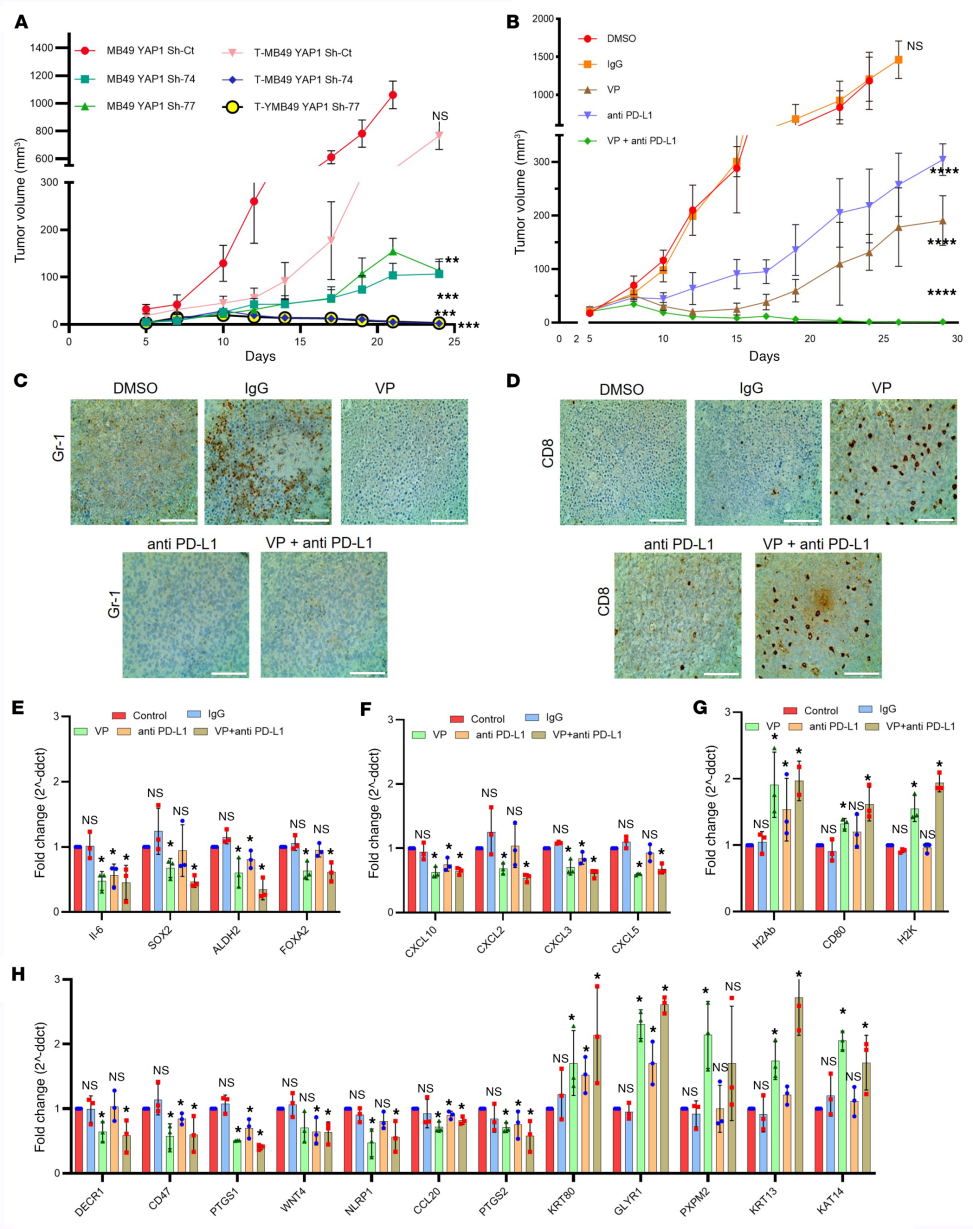
YAP1 inhibition shows synergistic antitumor efficacy in combination with anti–PD-L1. (**A**) Tumor growth curve of genetically YAP1-attenuated MB49 cells (YAP1 Sh) treated with anti–PD-L1. T, treated group. (**B**) WT MB49 cells were subcutaneously injected into C57BL/6 mice and treated with VP, anti–PD-L1, and combination of VP and anti–PD-L1. Tumor growth was monitored at the indicated times. (**C**) IHC showing the expression of MDSCs (Gr-1) in xenograft tissues obtained from VP-, anti–PD-L1–, or VP + anti–PD-L1–treated MB49 WT tumors after the completion of treatment (25 days). Scale bar: 100 μm. (**D**) IHC showing CD8^+^ cells in xenograft tissues obtained from VP-, anti–PD-L1–, or VP + anti–PD-L1–treated MB49 tumors after the completion of treatment (25 days). Scale bar: 100 μm. (**E**) RT-qPCR analysis of selected CSC markers using RNA from xenograft tumors treated with indicated drugs after the completion of treatment (25 days). (**F**) RT-qPCR analysis of different tumor-promoting CXCLs using the same RNA as in **E**. (**G**) RT-qPCR analysis of selected immunogenicity markers after the completion of treatment (25 days). (**H**) RT-qPCR analysis of various key immune-regulatory molecules after the completion of treatment. Data are presented as means ± SD of at least 3 independent experiments. *P < 0.05, **P < 0.01, ***P < 0.001, ****P < 0.0001 by 1-way ANOVA.

It was reported in different solid tumors that M2 macrophages and infiltration of MDSCs in the TME facilitate cancer cell resistance to ICIs or immunotherapy (15, 33, 39). Our data indicate that YAP1 expression in the cancer cells causes increased infiltration of MDSCs in the TME and induces the polarization of macrophages into M2 phenotype (Figure 4E and Figure 5, D–F), and RT-qPCR analysis revealed that genetic or pharmacological YAP1 attenuation downregulates the expression of PD-L1 in in vitro conditions (Supplemental Figure 8, A and B). We therefore hypothesized that ablation of YAP1 signaling might increase ICIs’ efficacy. Accordingly, we observed that anti–PD-L1 therapy was more effective in mice bearing YAP1-Sh MB49 tumors compared with mice bearing the YAP1 Sh-control MB49 tumors (Figure 10A). We then explored the combinatorial therapeutic efficacy of an anti–PD-L1 antibody (ICIs) and VP (YAP1 inhibitor) in YAP1-expressing WT MB49 cell–derived subcutaneous tumors in C57BL/6 mice. As expected, pharmacological inhibition of YAP1 in combination with anti–PD-L1 antibody showed significant tumor growth inhibition compared with any single therapy (Figure 10B and Supplemental Figure 8, C–H). To validate the target specificity of VP, immunoblot and RT-qPCR analysis confirmed the downregulation of YAP1 and YAP1 downstream targets (Supplemental Figure 9, A and B). Investigation of blood and other systemic toxicity parameters indicated that VP treatment did not pose any additional toxicity in the experimental mice (Supplemental Figure 9, C–J). At the end of the 30-day treatment regime, we found no tumors in 2 of 5 animals in the combinatorial drug-treated group. We further maintained these animals until 62 days after the treatment, and no tumor was found to appear in these mice. IHC analysis of the tumor tissue collected after the treatment protocol (5 weeks) from each cohort showed decreased numbers of MDSCs (Gr-1 positive) and an increased number of CD8^+^ T cells in the group treated with the combination of VP and anti–PD-L1 treated compared with either of the groups treated with a single drug (Figure 10, C and D). RT-qPCR analysis showed decreased expression of several CSC markers, IL-6, and CXCLs in the group treated with the combination of VP and anti–PD-L1 compared with each of the other groups (Figure 10, E and F). In addition, the combinatorial treatment regime also increased the level of cellular immunogenicity markers and candidate immune regulators (selected from the RNA-Seq data) in the tumor tissue (Figure 10, G and H). To investigate the antitumor memory in animals that were previously treated with both VP and anti–PD-L1, we challenged subcutaneous tumor growth in control mice (no tumor was grown in these mice previously) and selected 2 mice from the combination treatment group that showed no tumor at the end of treatment protocol. Interestingly, we found that previously drug-treated mice showed significant tumor growth inhibition compared with the control animals (Supplemental Figure 9K). We also checked the antitumor efficacy of VP in combination with anti–PD-L1 in another mouse CDX model (developed from subcutaneous implantation of UPPL1595 cells) and found data similar to those obtained with MB49 cells (Supplemental Figure 9K). Taken together, our data led us to conclude that YAP1 may have a plausible role in immune therapy resistance and attenuation of YAP1 signaling might be a promising way to improve the efficacy of immunotherapy in UCB. Furthermore, YAP1 attenuation may have potential to develop residual antitumor memory.

## Discussion

We previously reported that YAP1 inhibition in UCB and LUAD decreases CSC-promoting activity and increases the therapeutic efficacy of combination chemotherapy (6, 7). Our findings in this study suggest that YAP1 induces immune suppression in UCB, and comprehensive investigation of the YAP1-regulated TIME led us to test the hypothesis that YAP1 inhibition in combination with ICI might provide a novel therapeutic strategy to treat selective patients with UCB. Here we found that YAP1 expression facilitates immune evasion by the recruitment of MDSCs, polarization of macrophages to M2 phenotype, and exhaustion of CD8^+^ T cells. MDSCs are regarded as one of the major drivers of immune evasion and development of resistance against ICI therapy (33, 40). We found that YAP1 expression induces the expression of IL-6 and phosphorylated STAT3 in UCB cells, which is consistent with our recent report in LUAD (7). YAP1 drives cancer stemness in LUAD and UCB (6, 7), and different reports support that generation of CSCs is the primary step toward immune evasion (10, 11). Our hypothesis is further solidified by our immunotherapy clinical cohort analysis indicating that YAP1 expression was increased in the immunotherapy-nonresponsive group compared with the responsive group (Figure 6, A–C). In a recent study, analysis of several clinical cohorts (mostly with non-muscle-invasive bladder cancer) treated with BCG or ICIs demonstrated that drug response and prognosis were poor in the high-YAP1-expressing group (29).

In preclinical studies using cell lines and mice, we found that YAP1 is a critical determinant of immune evasion. Bioinformatic data from the TCGA-BLCA database and molecular analysis of cell-derived xenografts showed that high YAP1 expression is correlated with high MDSC signatures in the TME. YAP1 induces the infiltration of MDSCs and decreases the CD8^+^ T cells in the TME. Further WT MB49 CDX analysis from anti-MDSC antibody–treated animals showed an increase of CD8^+^ T cells in the TME along with tumor regression. Therefore, increased infiltration of CD8^+^ T cells was expected in the YAP1-Sh MB49 CDX TME due to decreased infiltration of MDSCs and due to YAP1 downregulation favoring M1 macrophage polarization. Previously, researchers have identified an immunoregulatory role of different oncogenes like KRAS, cMYC, etc. and have shown that blocking these genes effectively enhances the efficacy of immunotherapy (41–43). A recent study indicated the potential role of YAP1 in regulating the infiltration of MDSCs and CD8^+^ T cells in prostate cancer (44). However, to our knowledge this is the most comprehensive assessment of the important role of YAP1 in the TIME in UCB.

A recent study reported that the IL-6/STAT3 signaling cascade is the driving factor in the induction of a “cold” TME through the regulation of MDSCs and exhaustion of CD8^+^ T cells (45). In preclinical studies, we found that IL-6/STAT3 expression was negatively associated with CD8^+^ T cell infiltration and was positively associated with MDSC infiltration. However, TCGA-BLCA database analysis showed that there is a very poor correlation between CD8^+^ T cell infiltration and IL-6 expression in the TME (data not shown). IL-6/STAT3 signaling is found to be activated in many solid cancers and is associated with poor prognosis (11). IL-6 was also shown to regulate MDSCs and CD8^+^ T cell activity in the TME (46). The discrepancy between our preclinical data and TCGA data and the association of IL-6/STAT3 and CD8^+^ T cell infiltration may be due to multiple cellular components in TCGA data compared with our CDX model. A pure genetic model of UCB may allow us to appropriately study the signaling dynamics. Different carcinogen-induced and engraftment models are highly accepted in studying UCB, but compared with other cancer types UCB is underrepresented by genetically engineered mouse models (47).

Accumulated evidence suggests that the coordinated action of multiple signaling intermediates and their interaction with different immune cell types facilitate immune evasion in UCB and other solid tumors (48, 49). We found that YAP1 attenuation results in downregulation of several CXCR2-associated ligands in cancer cells and in xenograft tumors. It was reported that overexpression of some of CXCR2-associated ligands are linked with non-responsiveness to immune therapy in pancreatic ductal adenocarcinoma (50). Therefore, although further study is needed, it is likely that YAP1 attenuation may enhance the therapeutic efficacy of ICI by decreasing CXCR2 and its associated ligands in UCB. To validate the association of YAP1 and the IL-6/STAT3/CXCR2 signaling pathway for inducing immunosuppressive TME, we treated WT MB49-derived CDX–bearing mice with a STAT3 inhibitor. Our findings indicate that inhibition of STAT3 downregulates different CXCR2-associated ligands, and this observation led us to conclude that YAP1 regulates the expression of CXCLs through STAT3-mediated signaling in UCB. These findings open a new direction to explore whether YAP1 inhibition induces adaptive immunity and improves the efficacy of ICIs.

The mechanisms of YAP1-regulated immune modulation remain incompletely understood. Strikingly, simultaneous injection of YAP1-Sh cells and WT cancer cells (MB49) in the same mice showed decreased growth of the WT CDX compared with stand-alone WT xenograft grown in separate mice. Tumor histological and molecular analysis demonstrated decreased expression of IL-6 and CXCLs and a decreased ratio of MDSCs to CD8^+^ T cells in the WT CDX simultaneously injected with the YAP1-Sh clones compared with the WT cell-derived tumors in separate mice. Although we did not exclusively explore the target(s) for the noticeably slower growth of WT tumors in dually implanted tumors in the same mice, our preliminary experiments revealed that YAP1-Sh cells release significantly more EVs in the cell culture medium compared with the WT cells and that these EVs are loaded with more proteins. Further molecular discernment of these EVs will help to determine the YAP1-regulated immunomodulatory target(s) that facilitate immunosuppressive TME and will open a fertile avenue to investigate the mechanism of induction of adaptive immunity in YAP1-Sh UCB cells. In the TME, lipid droplets (LDs) have been shown to have the potential to induce cancer stemness and immunosuppression by regulating the macrophage phenotype (51, 52). Although identification of the precise targets for enhanced accumulation of LD is beyond the scope of the present study, our data showed that YAP1 expression is correlated with the intracellular accumulation of LDs (Figure 8, A and B).

Different mechanisms of immune evasion have been identified in different cancer types for the non-responsiveness to ICI, such as T cell exhaustion, macrophage polarization, deregulation of IFNs and ILs signaling, and recruitment of immunosuppressive cells in the TME (53). Few studies have been conducted in UCB to decipher the underlying mechanism of non-responsiveness to ICI (54, 55). Although further studies are needed, we observed that YAP1 induces an immunosuppressive TIME by modulating the expression of key signaling molecules that correlate with high MDSC recruitment and T cell exhaustion (Figure 3A and Figure 4A) (56, 57). In addition, overexpression of YAP1 induces the polarization of macrophages into the M2 phenotypes (tumor-associated macrophages) by regulating different genes such as iNOS, MerTK, IL-10, STAT3, CD163, CD206, Arg-1, CD86, and TNF-α in the naive macrophages. Altogether, our findings suggest that immune evasion by YAP1 is a complex process and the accumulated effects regulated by YAP1 result in resistance to ICIs. Effective therapeutic strategies combining MDSC inhibitors and ICI have been tested in many clinical trials (such as NCT003302247, ClinicalTrials.gov), and preclinical studies have also been carried out combining CXCR2 inhibitors and ICI (42, 58). Currently, a phase I clinical trial has also been started in melanoma combining SX-682 (CXCR1/2 inhibitor) with ICI (NCT03161431). We speculate that inhibition of YAP1 signaling in combination with ICI may be more effective, as YAP1 has effects on MDSC infiltration, CXCR2 expression, and macrophage polarization.

Our study revealed that downregulation of YAP1 in mouse UCB cell lines can induce immunologically “hot” TME in response to genetic and pharmacological inhibition of YAP1, and analysis of publicly available clinical cohort data supports this phenomenon in human UCB samples. This observation allows us to hypothesize that YAP1 inhibition might be a possible way to enhance the efficacy of ICIs in UCB and other cancer types. Anti–PD-L1 and anti–PD-1 have been reported to be used in treating UCB (25, 59, 60). However, owing to a lack of knowledge about the regulation of these targets in the tumor cells as well as in the immune cells, it is still difficult for clinicians to choose the right option for their patients. In this study, combinatorial administration of verteporfin and anti–PD-L1 showed a synergistic effect in tumor growth inhibition. Interestingly, slower growth of tumors was observed after challenging of verteporfin- and anti–PD-L1–treated mice with fresh WT MB49 cells in comparison with tumors grown in non-treated mice injected with the same cells. Although they need to be confirmed in a larger set of animals, our findings indicate that mice with YAP1 suppression and anti–PD-L1 treatment may develop immune memory.

**Figure 11:**
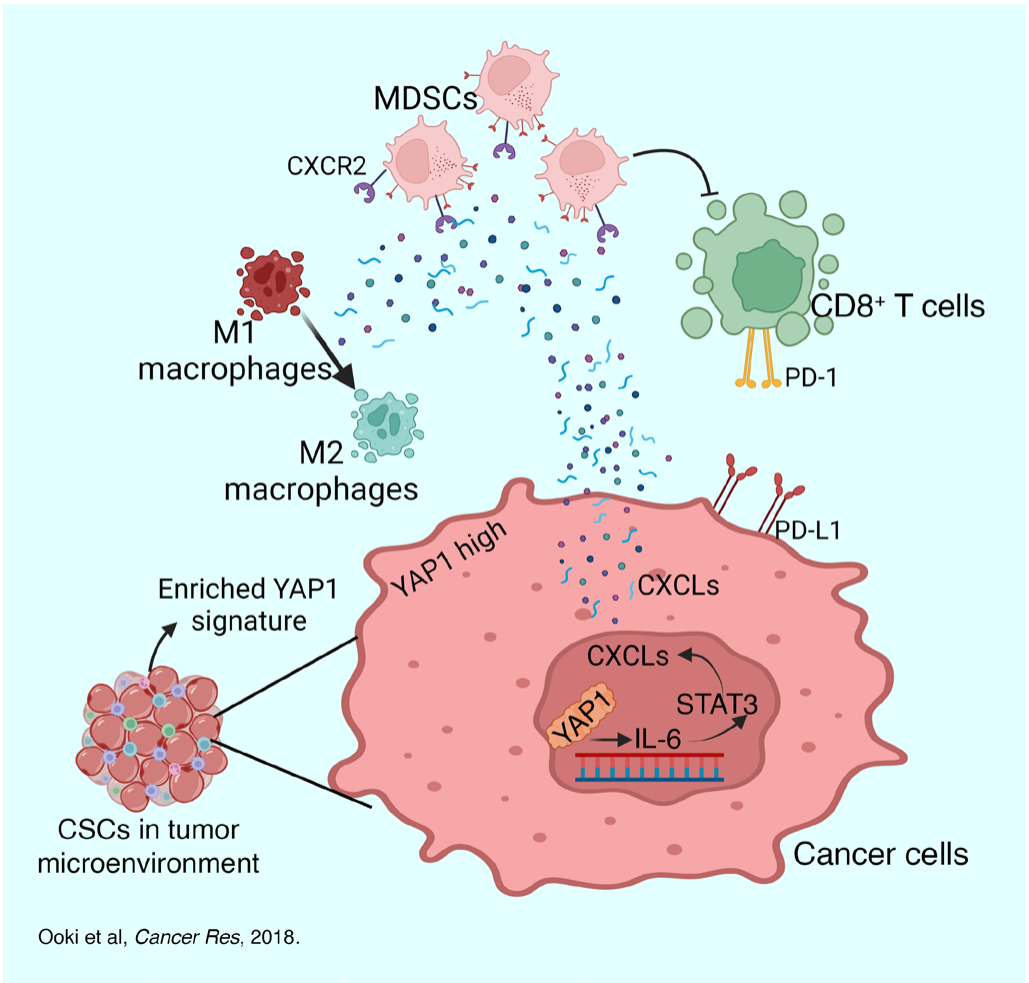
A schematic representation showing a possible mechanism of YAP1-driven induction of immunosuppression in UCB mediated by the IL-6/STAT3 pathway.

Our in vitro findings suggest that YAP1 inhibition induces a pathway associated with immune stimulation (Figure 11). RNA array–based findings were further supported by molecular analysis of the tumor tissues derived from mice across treatment regimens. For example, combinatorial treatment with verteporfin and anti–PD-L1 increases the CD8^+^/MDSC ratio in the tumor tissue compared with anti–PD-L1 alone. Thus, a combination of a YAP1 inhibitor and anti–PD-L1 would be a plausible therapeutic approach for patients with YAP1-expressing tumors. Further studies are needed to conclude whether YAP1 can be a marker for determining this combinatorial therapy. YAP1 inhibition with ICI should also be done on other YAP1-expressing cancers with poor immunogenic response. A shortcoming of our study is that the immune system and response in mice greatly vary from those in humans, and it remains to be elucidated whether this therapeutic approach will be effective in humans. In our previous studies, we found that YAP1 inhibition in combination with chemotherapy is effective against patient-derived xenograft (PDX) of UCB and LUAD (6, 7), and future studies will include PDX models in humanized mice to get comparatively more human-relevant data. Nonetheless, further studies exploring the therapeutic potential of modulating YAP1 and downstream molecules may yield important translational data with clinical relevance.

## Methods

### Sex as a biological variable.

Our study examined male and female animals, and similar findings are reported for both sexes.

### Cell lines, constructs, and mice.

Several mouse (MB49, UPPL1595, and BBN975) and human cell lines (BFTC905, BFTC909, T24, and UMUC3) were used in this study. MB49 cells were maintained in DMEM (Mediatech) with 10% fetal bovine serum (FBS; Hyclone). The BBN975 cells were maintained in RPMI 1640 medium (Mediatech) with 10% FBS. The UPPL1595 cells were maintained in MEM (Mediatech) with 10% FBS, vitamin solution, sodium pyruvate, non-essential amino acids, and HEPES. All human cell lines (BFTC905, BFTC909, T24, and UMUC3) were maintained in DMEM (Mediatech) with 10% FBS (Hyclone). All the cells were cultured under a 5% CO_2_ atmosphere at 95% relative humidity. Further details are available in Supplemental Methods.

YAP1 shRNA pGFP-C-shLenti Vector (YAP1-Sh) was used for the knockdown (KD) of the gene expression (Origene). Non-effective 29-mer scrambled shRNA pGFP-C-shLenti Vector (Origene) was used as a control (YAP1-Ctrl). For the siRNA-mediated knockdown of YAP1, YAP1 Silencer Select siRNA (Thermo Fisher Scientific) was used.

Details of mouse experiments are available in Supplemental Methods.

### Cell viability assay.

Cell proliferation and viability were evaluated using alamarBlue Cell Viability Reagent (Thermo Fisher Scientific). Briefly, cells (5 × 10^3^ per well) were seeded into 96-well plates with culture medium containing 10% FBS, and the optical density of each well was measured following the manufacturer’s protocol. The absorbance was measured at desired time intervals by a SpectraMax 250 Plate Reader (Molecular Devices). Cell viabilities were calculated as percentage over control.

### Sphere formation assay.

Sphere formation was induced by culturing of cells (2 × 10^4^ per well) in DMEM/Ham’s F12 50/50 Mix (Mediatech) supplemented with B-27 (Life Technologies), 20 ng/mL FGF-basic (PeproTech), and 20 ng/mL EGF (PeproTech). Cell culture was performed in ultra-low-attachment 6-well plates (Corning) for 10 days. The medium was replaced every other day. Sphere formation was evaluated using the inverted phase-contrast microscope.

### Transcriptomic and gene expression analysis.

Total RNA from the cultured cells was extracted using Monarch Total RNA Miniprep Kit (NEB T2010S, New England Biolabs). RNA-Seq libraries were prepared using the NE Next Ultra II RNA Library Prep Kit for Illumina (NEB E7775, New England Biolabs) from total RNA, following the manufacturer’s protocol. Libraries were then sequenced on a NovaSeq 6000 using 2 × 50 bp paired-end reads to an average depth of about 52 million reads per sample. Transcriptomic data collected by RNA sequencing (RNA-Seq) were analyzed to determine the genes present in each sample and condition, their expression levels, and the differences between expression levels among experiment conditions, as follows. Sequencing reads were mapped to the mouse genome version GRCm39 with the alignment tool STAR v2.7.6a (61), which allows for large “gaps” in the alignment, representing introns. The aligned reads were assembled with PsiCLASS v1.0.2 (62) to create gene and transcript models. Transcripts were then assigned to known genes from the GENCODE vM.28 reference gene set. Lastly, DESeq2 (63) was used to quantify the expression levels and determine differentially expressed genes. Additional visualizations, including volcano plots and plots of principal coordinate analysis components, were generated with custom R scripts. Differentially expressed genes between YAP1-Sh and YAP1 Sh-control cells were defined as log_2_(fold change) greater than 1 (up) or log_2_(fold change) less than −1 (down) and FDR less than 0.05. Data were deposited in the NCBI’s Gene Expression Omnibus database (GEO23174).

### Bioinformatics analysis.

IMvigor210 RNA-Seq data and clinical outcomes were downloaded from R package IMvigor210CoreBiologies (32). To evaluate the association between immunotherapy responses and expressions of YAP1 and IL-6, gene set variation analysis (GSVA) was performed. Samples were equally separated into “GSVA_high” and “GSVA_low” groups based on their GSVA enrichment score. To generate heatmaps and box plots based on responses, samples of “NE” (not estimable) in Best Confirmed Overall Response were excluded. Samples with “CR” (complete response) and “PR” (partial response) were considered as “Response” group, and samples with “SD” (stable disease) and “PD” (progressive disease) as “Nonresponse” group. GSE186043 cohort data were downloaded from GEO to evaluate the effect of YAP1 knockdown in the UC3 cell line. Details of bioinformatics analysis are available in Supplemental Methods.

### Macrophage isolation and migration assay.

Eight-week-old C57BL/6 mice were intraperitoneally injected with 3% Brewer thioglycollate medium, and intraperitoneal macrophages were harvested 3 days after treatment. Cell migration assay was performed using a Transwell coculture system in 24-well plates (Corning) as described previously (44, 64). Details are available in Supplemental Methods.

### MDSC isolation and migration assay.

MDSCs were isolated from the tumor mass developed from MB49 YAP1-Sh and Sh-control clones using a Mouse MDSC Isolation Kit (Miltenyi Biotec, catalog 130-094-538) and plated in RPMI 1640 supplemented with 10% FBS and antibiotics. MDSCs (1 × 10^5^ cells per well) were seeded in the top chamber of the Transwell (Corning) plate. Conditioned medium (CM) from cultured MB49 clones (YAP1 Sh-control, Sh-74, Sh-77) was collected and added to the bottom layer of the Transwell. After 4 hours of incubation, cells that had completely migrated to the bottom chamber were counted (44).

### Enzyme-linked immunosorbent assay.

Cells (2 × 10^6^ per 100 mm dish) were cultured for 24 hours. Media were removed and replaced with 10 mL serum-free DMEM. Supernatants were collected 24 hours later with any floating cells removed by 0.45 mm filtration. Enzyme-linked immunosorbent assay (ELISA) was performed for IL-10, TNF-α, IL-6, and STAT3 according to the manufacturer’s instructions (R&D Systems).

### In vivo xenograft assay and treatment.

For in vivo xenograft, cells (MB49/UPPL1595/BBN975) were suspended in 100 μL of a 1:1 mixture of serum-free DMEM and Cultrex Stem Cell Qualified Reduced Growth Factor Basement Membrane Extract (Trevigen), and then injected subcutaneously into the flank of C57BL/6 mice. Details are available in Supplemental Methods.

### Flow cytometric analysis.

The dissociated tumor cells were passed through a 70 μm cell strainer (BD), and cells were stained with Zombie Aqua Viability dye (BioLegend, catalog 423102) according to the manufacturer’s instructions. After the Zombie dye buffer was removed, the Fc receptors were blocked with antibodies (BioLegend, catalog 101320). Cells were then incubated for 30 minutes at 4°C for surface marker targets listed in Supplemental Table 1. After being washed several times with FACS buffer, cells were exposed to 1× BD FACS lysing buffer (BD Biosciences, catalog 349202). Intracellular staining was then performed for proteins of interest following the BD Cytofix/Cytoperm (BD Biosciences, catalog BD554714) protocol. Gating schemes are available in Supplemental Methods. Data were acquired on a BD FACSCalibur flow cytometer (BD Biosciences) using BD CellQuest Pro software (BD Biosciences) and analyzed with FlowJo software v10.1 (BD Biosciences).

RAW 264.7 macrophages were cultured with CM obtained from confluent MB49 YAP1-Sh cells (constructs Sh-control, Sh-74, and Sh-77) for 24 hours in 24-well format. The macrophages were treated with 0.05% trypsin-EDTA (Thermo Fisher Scientific, catalog 253000054) and resuspended in RAW 264.7 medium in a U-bottom 96-well plate for subsequent flow cytometric staining. The macrophages were first stained with Zombie Aqua Viability dye (BioLegend, catalog 423102) according to the manufacturer’s instructions. Upon removal of the Zombie dye buffer, the Fc receptors were blocked with antibodies (BioLegend, catalog 101320). Cells were then stained for 30 minutes at 4°C for surface marker targets (Supplemental Table 2). After several washes, cells were preserved in 1× BD FACS lysing buffer (BD Biosciences, catalog 349202). Intracellular staining was then performed for cytokines of interest following the BD Cytofix/Cytoperm (BD Biosciences, catalog BD554714) protocol. Details are available in Supplemental Methods.

### Estimation of intracellular lipid droplets and glycolytic activity.

Intracellular lipid droplet accumulation was measured in cultured cells following the manufacturer’s protocol (Cayman Chemical, item 500001). Glycolytic activity was measured in the cell culture medium of cultured cells following the manufacturer’s protocol (Cayman Chemical, item 600450). The absorbance was measured at 490 nm with Spectramax by Molecular Devices.

### Statistics.

In each set of data analyses, the estimate variation is indicated in each figure as an SD. The 2 groups were compared with 1-tailed Student’s *t* test. A comparison between the groups was performed using ANOVA for non-parametrically continuous variables. Categorical variables were analyzed using Fisher’s exact test. The level of statistical significance was set at *P* less than 0.05. All statistical analyses were conducted using the GraphPad Prism software package.

### Study approval.

All experiments using mice were approved by the Johns Hopkins University Animal Care and Use Committee, and the mice were maintained in accordance with the American Association of Laboratory Animal Care guidelines.

### Data availability.

Data were deposited in the NCBI’s Gene Expression Omnibus database (GEO23174). Values for all data points in graphs are reported in the Supporting Data Values file.

## Author contributions

PS, AO, and MOH conceived and designed the study. PS, MF, EJI, AO, WC, and MOH developed methodology. PS, AO, MF, WC, DS, DJM, BAJ, AM, and MOH performed acquisition of data (provided animals, acquired and managed patients, provided facilities, etc.). PS, MF, WC, AO, IS, LM, and MOH performed analysis and interpretation of data (e.g., statistical analysis, biostatistics, computational analysis). FCMS and MOH supervised the study.

## Supplementary Material

Supplemental data

Unedited blot and gel images

Supporting data values

## Version 1 12/04/2024

In-Press Preview

## Version 2 01/16/2025

Electronic publication

## Version 3 04/18/2025

Mohammad O. Hoque's name corrected in author list
