# Supplementary material for: YAP1 induces bladder cancer progression and promotes immune evasion through IL-6/STAT3 pathway and CXCL deregulation
Source: J Clin Invest. 2024 Nov 21;135(2):e171164. doi: 10.1172/JCI171164 (PMC11735109; doi:10.1172/JCI171164)
Supplement: Supplemental data [file jci-135-171164-s012.pdf]

Supplemental Information

Supplementary figure legends

Figure S1. Key findings from the TCGA-BLCA database analysis, a cornerstone of our study, demonstrating the oncogenic relevance of YAP1 in bladder cancer

Figure S2. The immunoregulatory potential of YAP1 by RNA-seq data analysis

Figure S3. Correlation between M2 macrophages and non-responsiveness to immunotherapy

Figure S4. Correlation between CXCR2-associated ligands and YAP1 in *in vitro* and in our primary UCB cohort and TCGA cohort

Figure S5. YAP1 potentially correlates with IL6/STAT3 mediated signaling in affecting immunotherapy efficacy

Figure S6. YAP1 induces lipid droplet storage and enhances immune suppression

Figure S7. YAP1 regulates EV secretion from the MB49 UCB cells

Figure S8. YAP1 attenuation affects the efficacy of anti-PD-L1

Figure S9. VP attenuates YAP1 without any toxic effect on animal

Figure S1

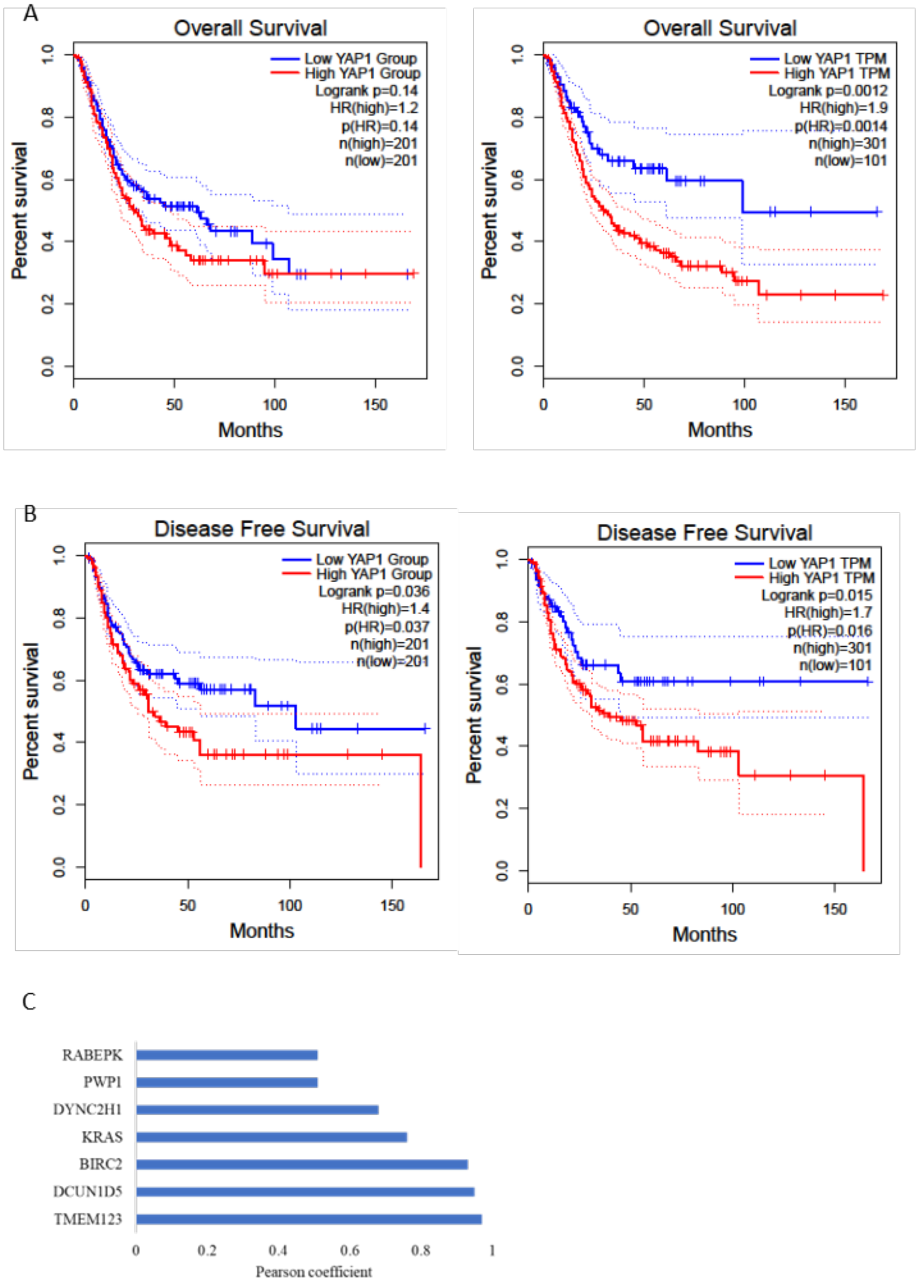
A
Overall Survival
Low YAP1 Group
High YAP1 Group
Logrank p=0.14
HR(high)=1.2
p(HR)=0.14
n(high)=201
n(low)=201
Percent survival
Months
Overall Survival
Low YAP1 TPM
High YAP1 TPM
Logrank p=0.0012
HR(high)=1.9
p(HR)=0.0014
n(high)=301
n(low)=101
Percent survival
Months
B
Disease Free Survival
Low YAP1 Group
High YAP1 Group
Logrank p=0.036
HR(high)=1.4
p(HR)=0.037
n(high)=201
n(low)=201
Percent survival
Months
Disease Free Survival
Low YAP1 TPM
High YAP1 TPM
Logrank p=0.015
HR(high)=1.7
p(HR)=0.016
n(high)=301
n(low)=101
Percent survival
Months
C
RABEPK
PWP1
DYNC2H1
KRAS
BIRC2
DCUN1D5
TMEM123
0
0.2
0.4
0.6
0.8
1
Pearson coefficient

D

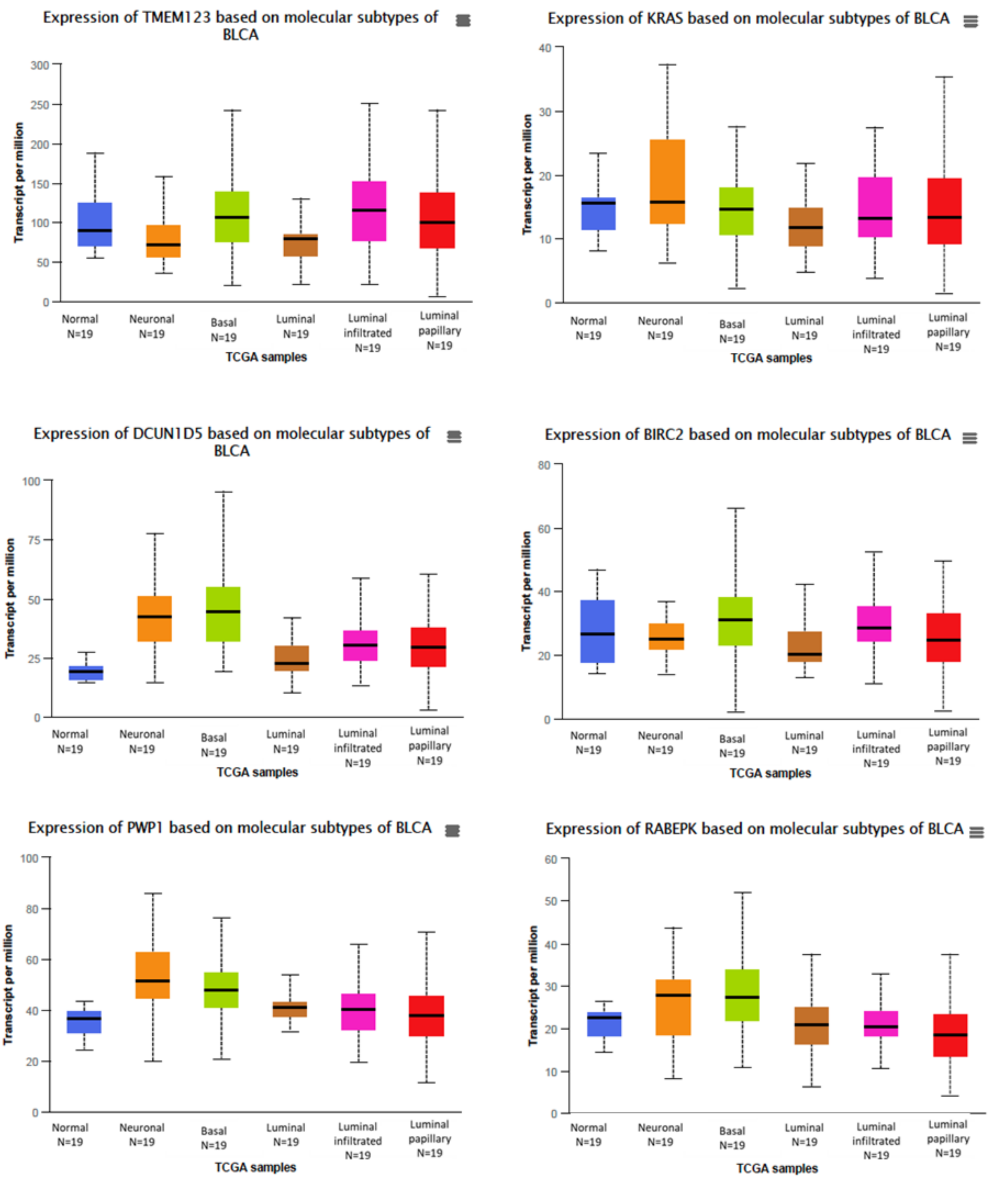
Expression of TMEM123 based on molecular subtypes of BLCA
Expression of KRAS based on molecular subtypes of BLCA
Expression of DCUN1D5 based on molecular subtypes of BLCA
Expression of BIRC2 based on molecular subtypes of BLCA
Expression of PWP1 based on molecular subtypes of BLCA
Expression of RABEPK based on molecular subtypes of BLCA
Transcript per million
Normal N=19
Neuronal N=19
Basal N=19
Luminal N=19
Luminal infiltrated N=19
Luminal papillary N=19
TCGA samples

E

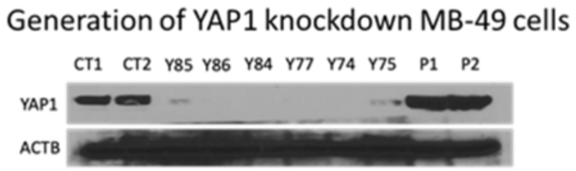
Generation of YAP1 knockdown MB-49 cells
CT1
CT2
Y85
Y86
Y84
Y77
Y74
Y75
P1
P2
YAP1
ACTB

F

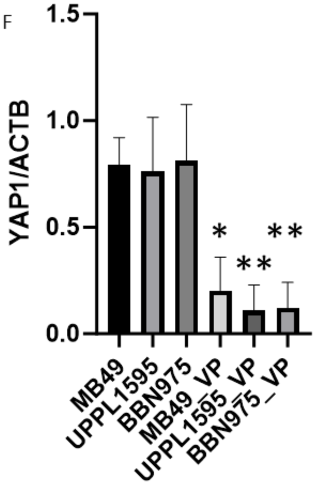
YAP1/ACTB
1.5
1.0
0.5
0.0
*
**
**
MB49
UPPL1595
BBN975
MB49_VP
UPPL1595_VP
BBN975_VP

G

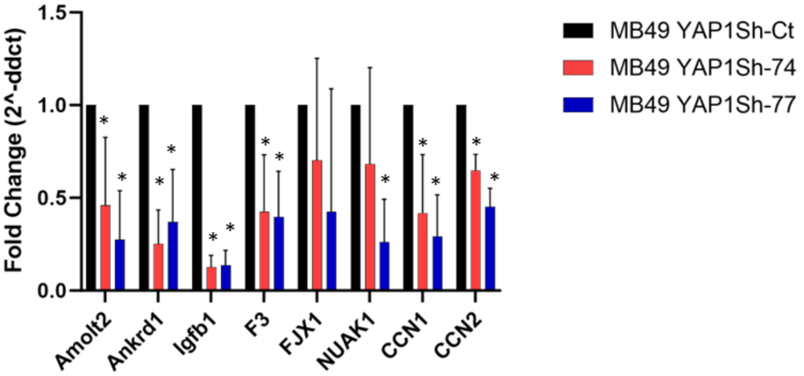
Fold Change (2^-ddct)
1.5
1.0
0.5
0.0
Amolt2
Ankrd1
Igfb1
F3
FJX1
NUAK1
CCN1
CCN2
MB49 YAP1Sh-Ct
MB49 YAP1Sh-74
MB49 YAP1Sh-77

H

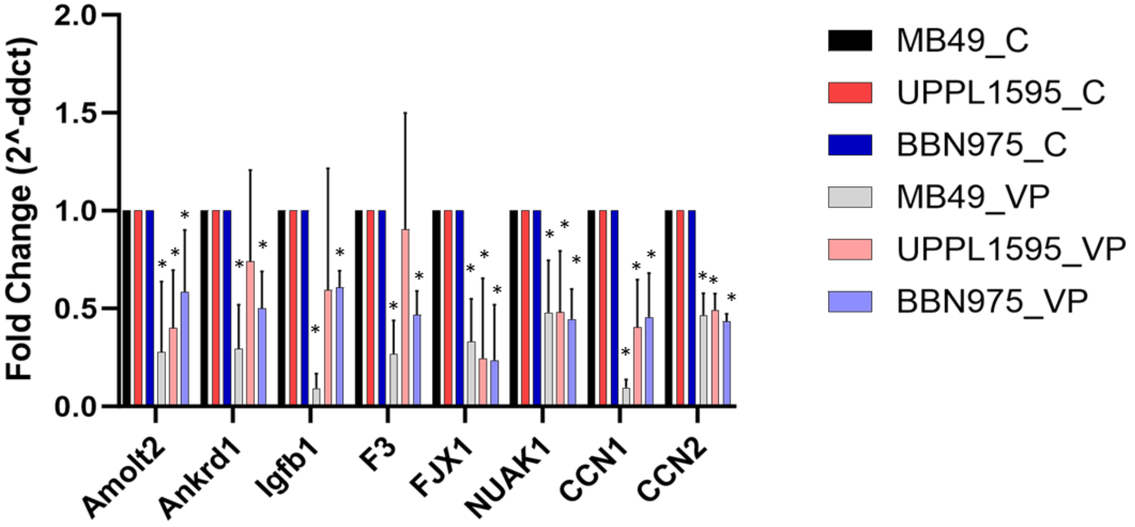
Fold Change (2^-ddct)
2.0
1.5
1.0
0.5
0.0
Amolt2
Ankrd1
Igfb1
F3
FJX1
NUAK1
CCN1
CCN2
MB49_C
UPPL1595_C
BBN975_C
MB49_VP
UPPL1595_VP
BBN975_VP

I

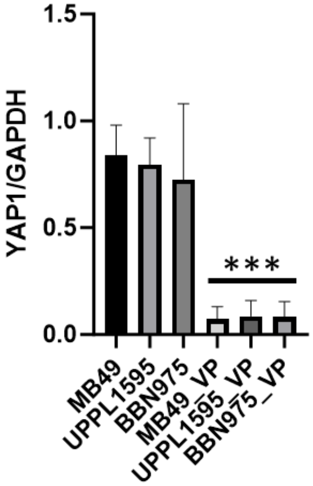
YAP1/GAPDH
1.5
1.0
0.5
0.0
***
MB49
UPPL1595
BBN975
MB49_VP
UPPL1595_VP
BBN975_VP

Figure S2

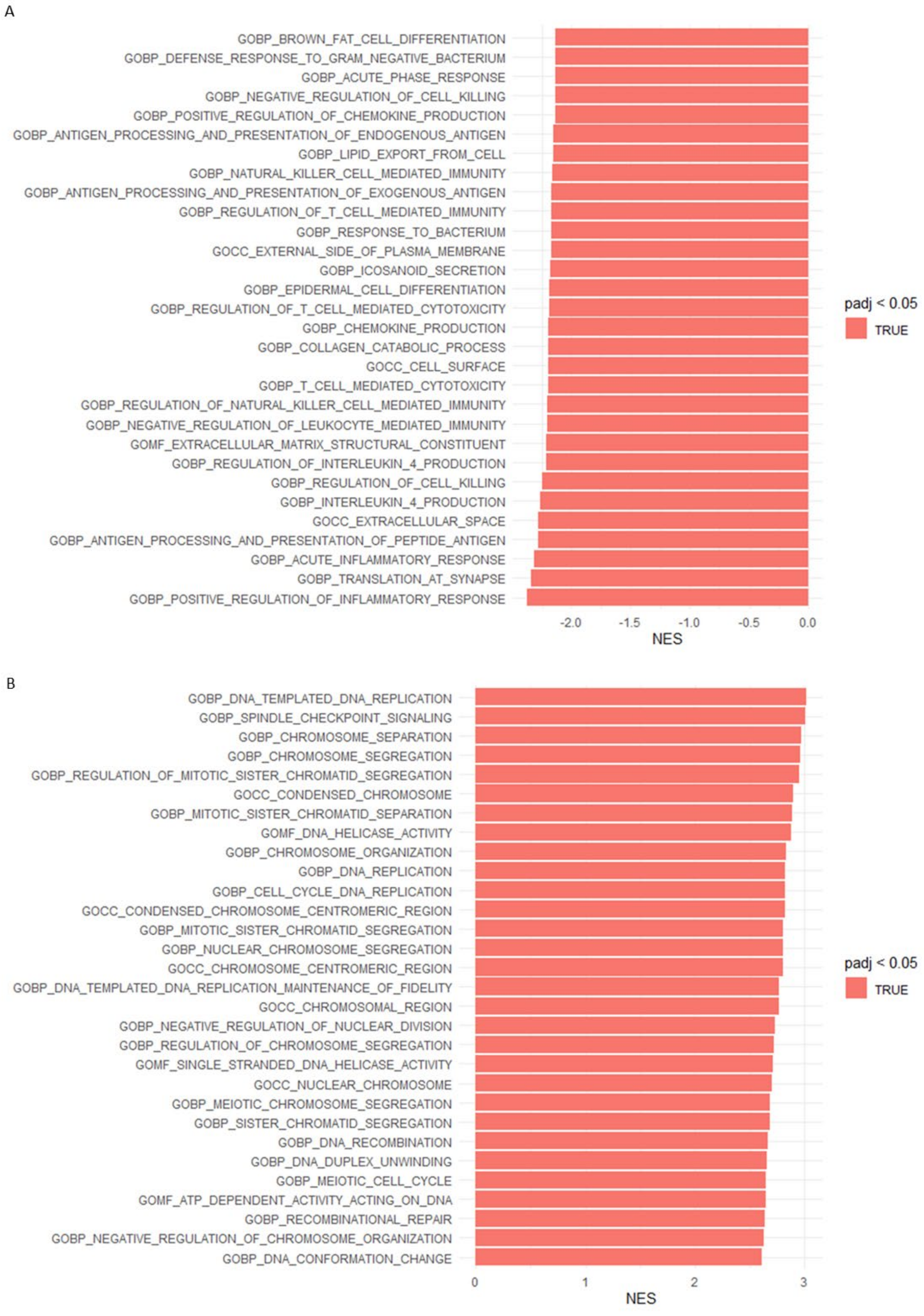

A
GOBP_BROWN_FAT_CELL_DIFFERENTIATION
GOBP_DEFENSE_RESPONSE_TO_GRAM_NEGATIVE_BACTERIUM
GOBP_ACUTE_PHASE_RESPONSE
GOBP_NEGATIVE_REGULATION_OF_CELL_KILLING
GOBP_POSITIVE_REGULATION_OF_CHEMOKINE_PRODUCTION
GOBP_ANTIGEN_PROCESSING_AND_PRESENTATION_OF_ENDOGENOUS_ANTIGEN
GOBP_LIPID_EXPORT_FROM_CELL
GOBP_NATURAL_KILLER_CELL_MEDIATED_IMMUNITY
GOBP_ANTIGEN_PROCESSING_AND_PRESENTATION_OF_EXOGENOUS_ANTIGEN
GOBP_REGULATION_OF_T_CELL_MEDIATED_IMMUNITY
GOBP_RESPONSE_TO_BACTERIUM
GOCC_EXTERNAL_SIDE_OF_PLASMA_MEMBRANE
GOBP_ICOSANOID_SECRETION
GOBP_EPIDERMAL_CELL_DIFFERENTIATION
GOBP_REGULATION_OF_T_CELL_MEDIATED_CYTOTOXICITY
GOBP_CHEMOKINE_PRODUCTION
GOBP_COLLAGEN_CATABOLIC_PROCESS
GOCC_CELL_SURFACE
GOBP_T_CELL_MEDIATED_CYTOTOXICITY
GOBP_REGULATION_OF_NATURAL_KILLER_CELL_MEDIATED_IMMUNITY
GOBP_NEGATIVE_REGULATION_OF_LEUKOCYTE_MEDIATED_IMMUNITY
GOMF_EXTRACELLULAR_MATRIX_STRUCTURAL_CONSTITUENT
GOBP_REGULATION_OF_INTERLEUKIN_4_PRODUCTION
GOBP_REGULATION_OF_CELL_KILLING
GOBP_INTERLEUKIN_4_PRODUCTION
GOCC_EXTRACELLULAR_SPACE
GOBP_ANTIGEN_PROCESSING_AND_PRESENTATION_OF_PEPTIDE_ANTIGEN
GOBP_ACUTE_INFLAMMATORY_RESPONSE
GOBP_TRANSLATION_AT_SYNAPSE
GOBP_POSITIVE_REGULATION_OF_INFLAMMATORY_RESPONSE
-2.0
-1.5
-1.0
-0.5
0.0
NES
padj < 0.05
TRUE
B
GOBP_DNA_TEMPLATED_DNA_REPLICATION
GOBP_SPINDLE_CHECKPOINT_SIGNALING
GOBP_CHROMOSOME_SEPARATION
GOBP_CHROMOSOME_SEGREGATION
GOBP_REGULATION_OF_MITOTIC_SISTER_CHROMATID_SEGREGATION
GOCC_CONDENSED_CHROMOSOME
GOBP_MITOTIC_SISTER_CHROMATID_SEPARATION
GOMF_DNA_HELICASE_ACTIVITY
GOBP_CHROMOSOME_ORGANIZATION
GOBP_DNA_REPLICATION
GOBP_CELL_CYCLE_DNA_REPLICATION
GOCC_CONDENSED_CHROMOSOME_CENTROMERIC_REGION
GOBP_MITOTIC_SISTER_CHROMATID_SEGREGATION
GOBP_NUCLEAR_CHROMOSOME_SEGREGATION
GOCC_CHROMOSOME_CENTROMERIC_REGION
GOBP_DNA_TEMPLATED_DNA_REPLICATION_MAINTENANCE_OF_FIDELITY
GOCC_CHROMOSOMAL_REGION
GOBP_NEGATIVE_REGULATION_OF_NUCLEAR_DIVISION
GOBP_REGULATION_OF_CHROMOSOME_SEGREGATION
GOMF_SINGLE_STRANDED_DNA_HELICASE_ACTIVITY
GOCC_NUCLEAR_CHROMOSOME
GOBP_MEIOTIC_CHROMOSOME_SEGREGATION
GOBP_SISTER_CHROMATID_SEGREGATION
GOBP_DNA_RECOMBINATION
GOBP_DNA_DUPLEX_UNWINDING
GOBP_MEIOTIC_CELL_CYCLE
GOMF_ATP_DEPENDENT_ACTIVITY_ACTING_ON_DNA
GOBP_RECOMBINATIONAL_REPAIR
GOBP_NEGATIVE_REGULATION_OF_CHROMOSOME_ORGANIZATION
GOBP_DNA_CONFORMATION_CHANGE
0
1
2
3
NES
padj < 0.05
TRUE

C

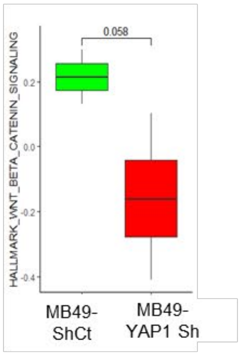
0.058
HALLMARK_WNT_BETA_CATENIN_SIGNALING
0.2
0.0
-0.2
-0.4
MB49-
ShCt
MB49-
YAP1 Sh

D

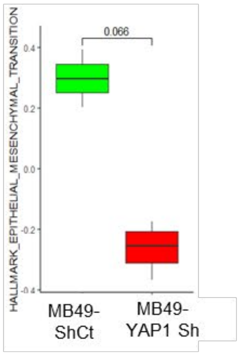
0.066
HALLMARK_EPITHELIAL_MESENCHYMAL_TRANSITION
0.4
0.2
0.0
-0.2
-0.4
MB49-
ShCt
MB49-
YAP1 Sh

E

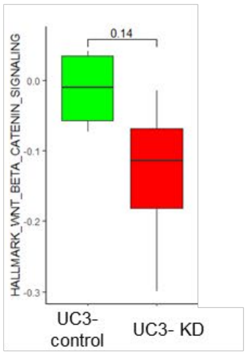
0.14
HALLMARK_WNT_BETA_CATENIN_SIGNALING
0.0
-0.1
-0.2
-0.3
UC3-
control
UC3- KD

F

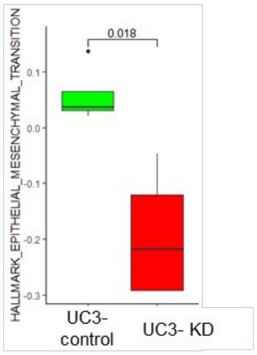
0.018
HALLMARK_EPITHELIAL_MESENCHYMAL_TRANSITION
0.1
0.0
-0.1
-0.2
-0.3
UC3-
control
UC3- KD

G

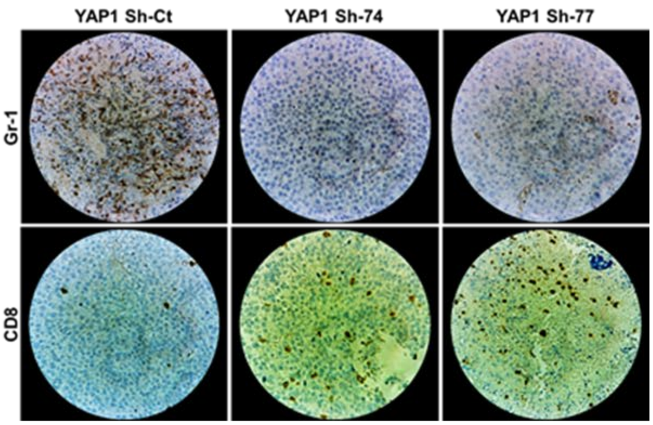
YAP1 Sh-Ct
YAP1 Sh-74
YAP1 Sh-77
Gr-1
CD8

H

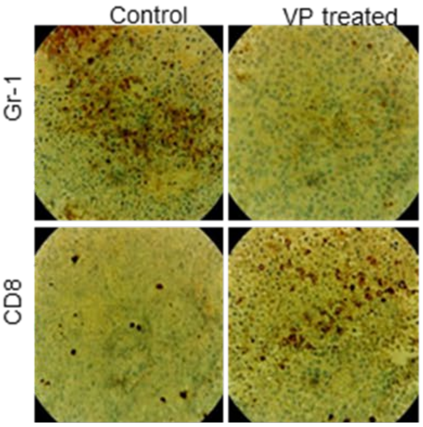
Control
VP treated
Gr-1
CD8

I

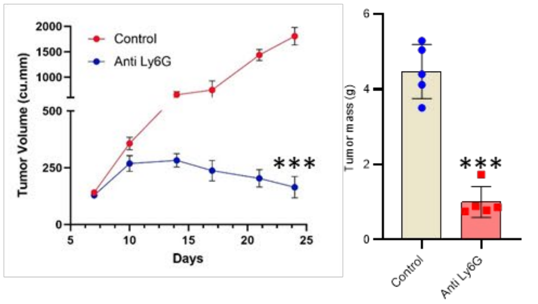
Control
Anti Ly6G
Tumor Volume (cu.mm)
2000
1500
1000
500
250
0
5
10
15
20
25
Days
***
Tumor mass (g)
6
4
2
0
***
Control
Anti Ly6G

Figure S3

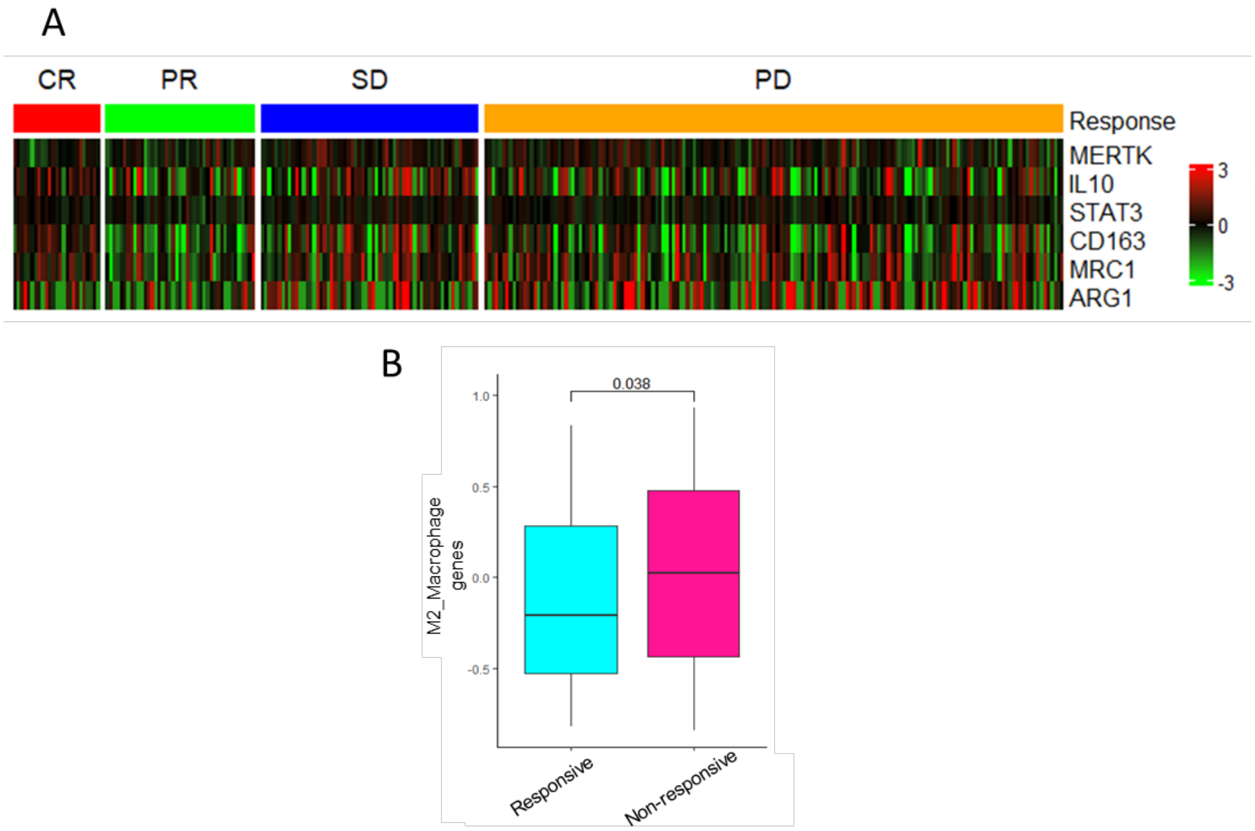
A
CR
PR
SD
PD
Response
MERTK
IL10
STAT3
CD163
MRC1
ARG1
3
0
-3
B
0.038
1.0
0.5
0.0
-0.5
M2_Macrophage genes
Responsive
Non-responsive

Figure S4

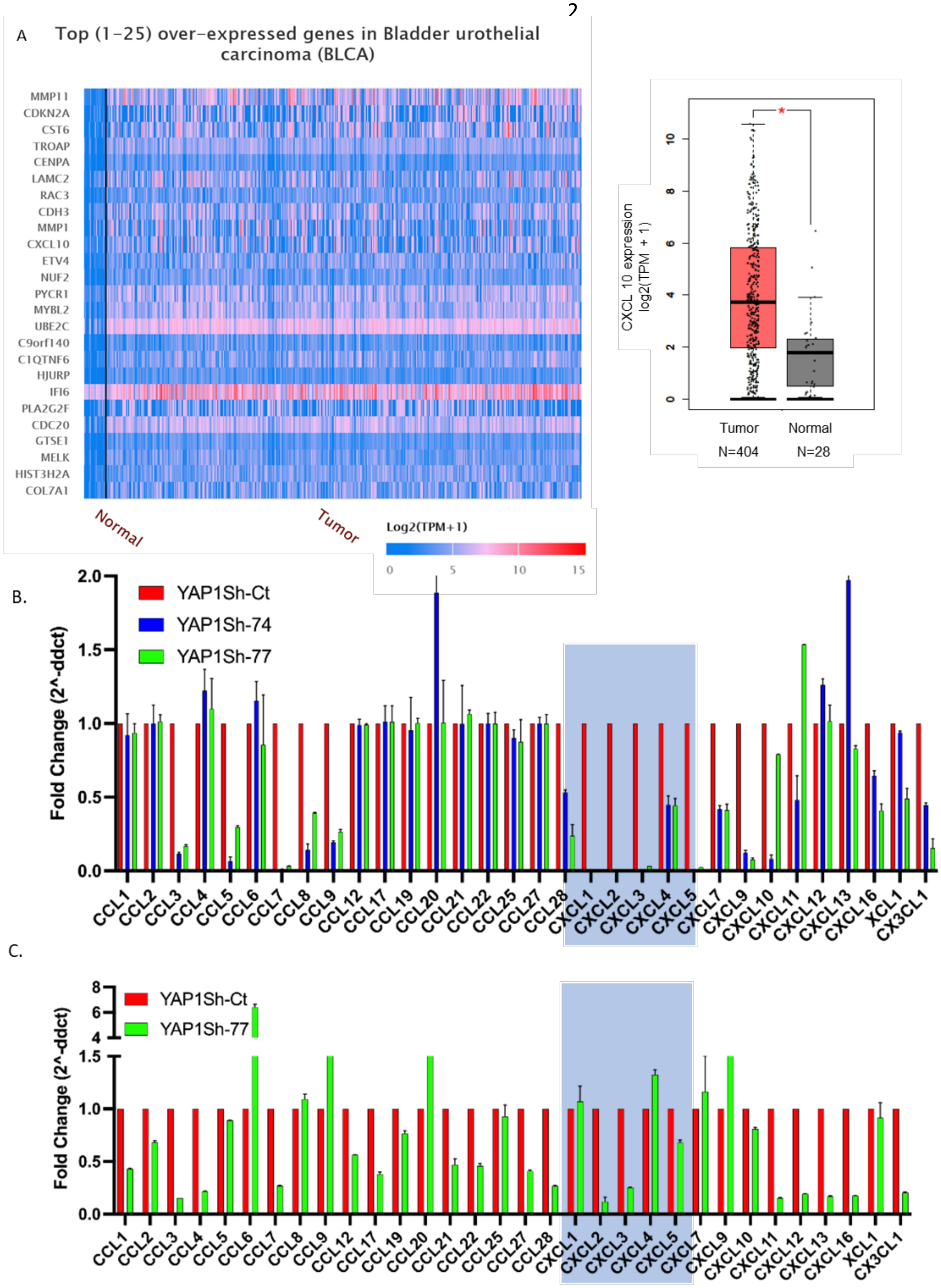
A
Top (1-25) over-expressed genes in Bladder urothelial carcinoma (BLCA)
MMP11
CDKN2A
CST6
TROAP
CENPA
LAMC2
RAC3
CDH3
MMP1
CXCL10
ETV4
NUF2
PYCR1
MYBL2
UBE2C
C9orf140
C1QTNF6
HJURP
IFI6
PLA2G2F
CDC20
GTSE1
MELK
HIST3H2A
COL7A1
Normal
Tumor
Log2(TPM+1)
0
5
10
15
CXCL 10 expression log2(TPM + 1)
Tumor N=404
Normal N=28
B.
Fold Change (2^-ddct)
YAP1Sh-Ct
YAP1Sh-74
YAP1Sh-77
CCL1
CCL2
CCL3
CCL4
CCL5
CCL6
CCL7
CCL8
CCL9
CCL12
CCL17
CCL19
CCL20
CCL21
CCL22
CCL25
CCL27
CCL28
CXCL1
CXCL2
CXCL3
CXCL4
CXCL5
CXCL7
CXCL9
CXCL10
CXCL11
CXCL12
CXCL13
CXCL16
XCL1
CX3CL1
C.
Fold Change (2^-ddct)
YAP1Sh-Ct
YAP1Sh-77

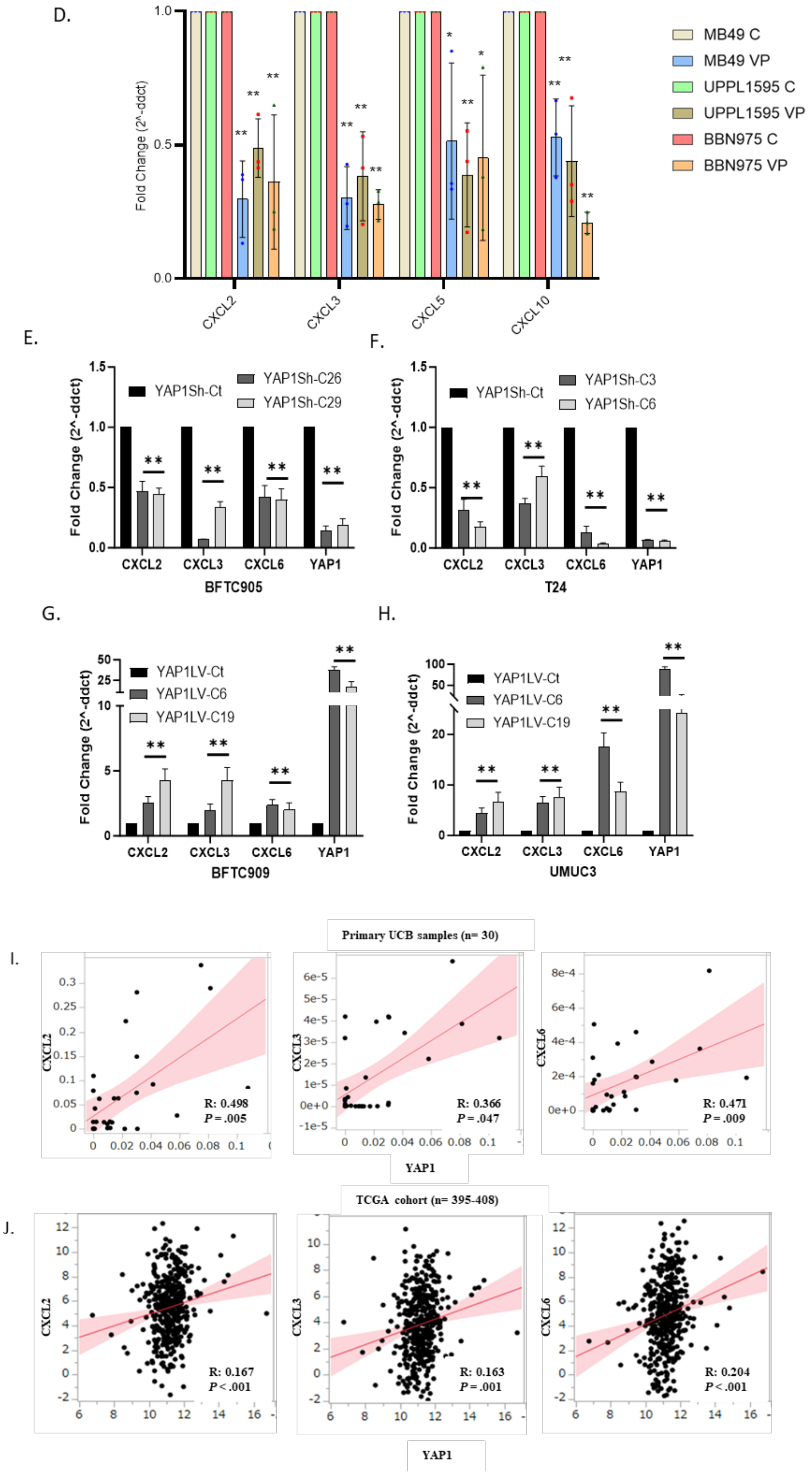
D.
Fold Change (2^-ddct)
1.0
0.5
0.0
CXCL2
CXCL3
CXCL5
CXCL10
MB49 C
MB49 VP
UPPL1595 C
UPPL1595 VP
BBN975 C
BBN975 VP
E.
Fold Change (2^-ddct)
YAP1Sh-Ct
YAP1Sh-C26
YAP1Sh-C29
CXCL2
CXCL3
CXCL6
YAP1
BFTC905
F.
YAP1Sh-Ct
YAP1Sh-C3
YAP1Sh-C6
CXCL2
CXCL3
CXCL6
YAP1
T24
G.
Fold Change (2^-ddct)
YAP1LV-Ct
YAP1LV-C6
YAP1LV-C19
CXCL2
CXCL3
CXCL6
YAP1
BFTC909
H.
Fold Change (2^-ddct)
YAP1LV-Ct
YAP1LV-C6
YAP1LV-C19
CXCL2
CXCL3
CXCL6
YAP1
UMUC3
Primary UCB samples (n= 30)
I.
CXCL2
R: 0.498
P = .005
CXCL3
R: 0.366
P = .047
CXCL6
R: 0.471
P = .009
YAP1
TCGA cohort (n= 395-408)
J.
CXCL2
R: 0.167
P < .001
CXCL3
R: 0.163
P = .001
CXCL6
R: 0.204
P < .001
YAP1

Figure S5

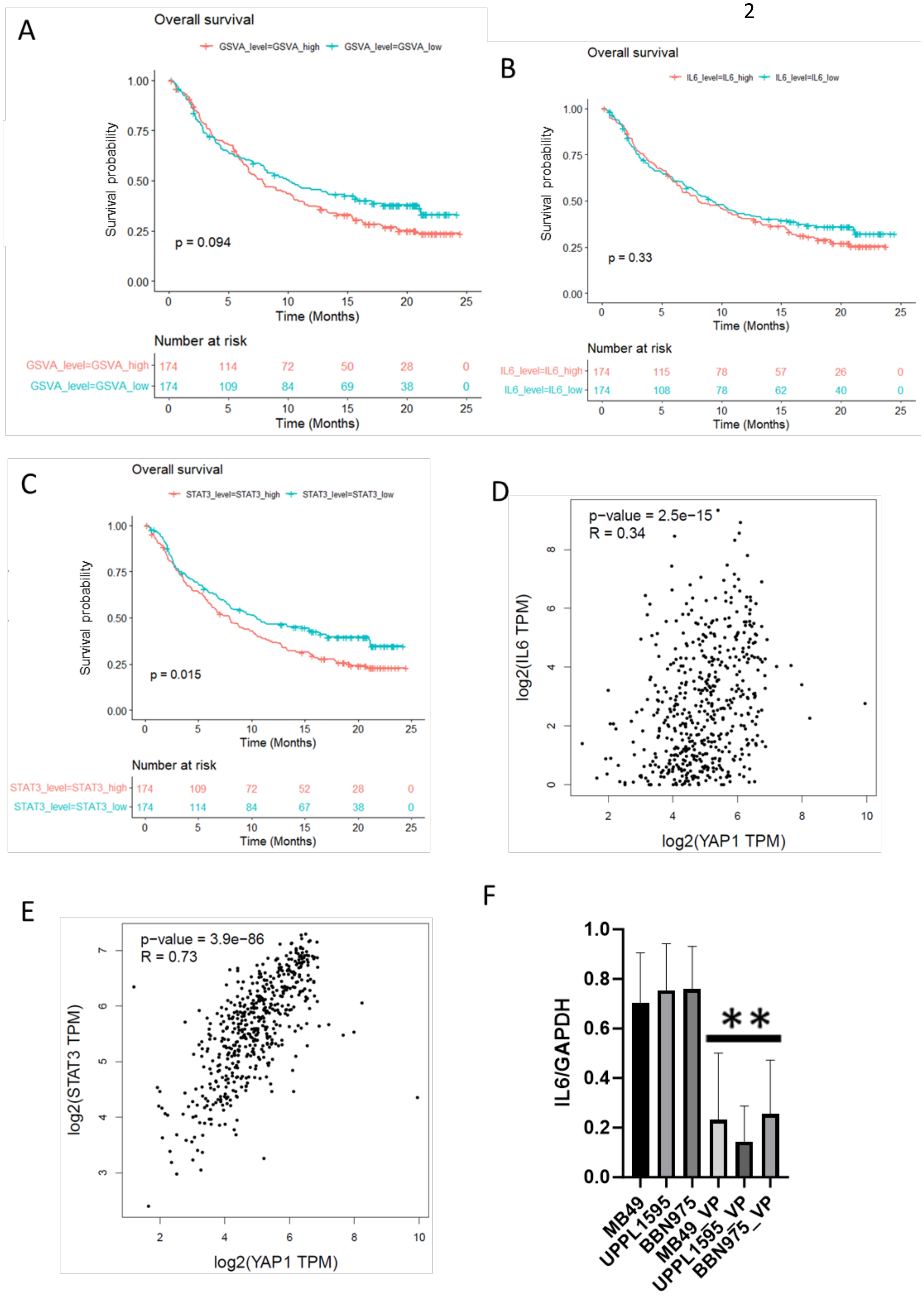

A
Overall survival
GSVA_level=GSVA_high
GSVA_level=GSVA_low
Survival probability
p = 0.094
Time (Months)
Number at risk
GSVA_level=GSVA_high 174 114 72 50 28 0
GSVA_level=GSVA_low 174 109 84 69 38 0
B
Overall survival
IL6_level=IL6_high
IL6_level=IL6_low
Survival probability
p = 0.33
Time (Months)
Number at risk
IL6_level=IL6_high 174 115 78 57 26 0
IL6_level=IL6_low 174 108 78 62 40 0
C
Overall survival
STAT3_level=STAT3_high
STAT3_level=STAT3_low
Survival probability
p = 0.015
Time (Months)
Number at risk
STAT3_level=STAT3_high 174 109 72 52 28 0
STAT3_level=STAT3_low 174 114 84 67 38 0
D
p−value = 2.5e−15
R = 0.34
log2(IL6 TPM)
log2(YAP1 TPM)
E
p−value = 3.9e−86
R = 0.73
log2(STAT3 TPM)
log2(YAP1 TPM)
F
IL6/GAPDH
**
MB49
UPPL1595
BBN975
MB49_VP
UPPL1595_VP
BBN975_VP

Figure S6

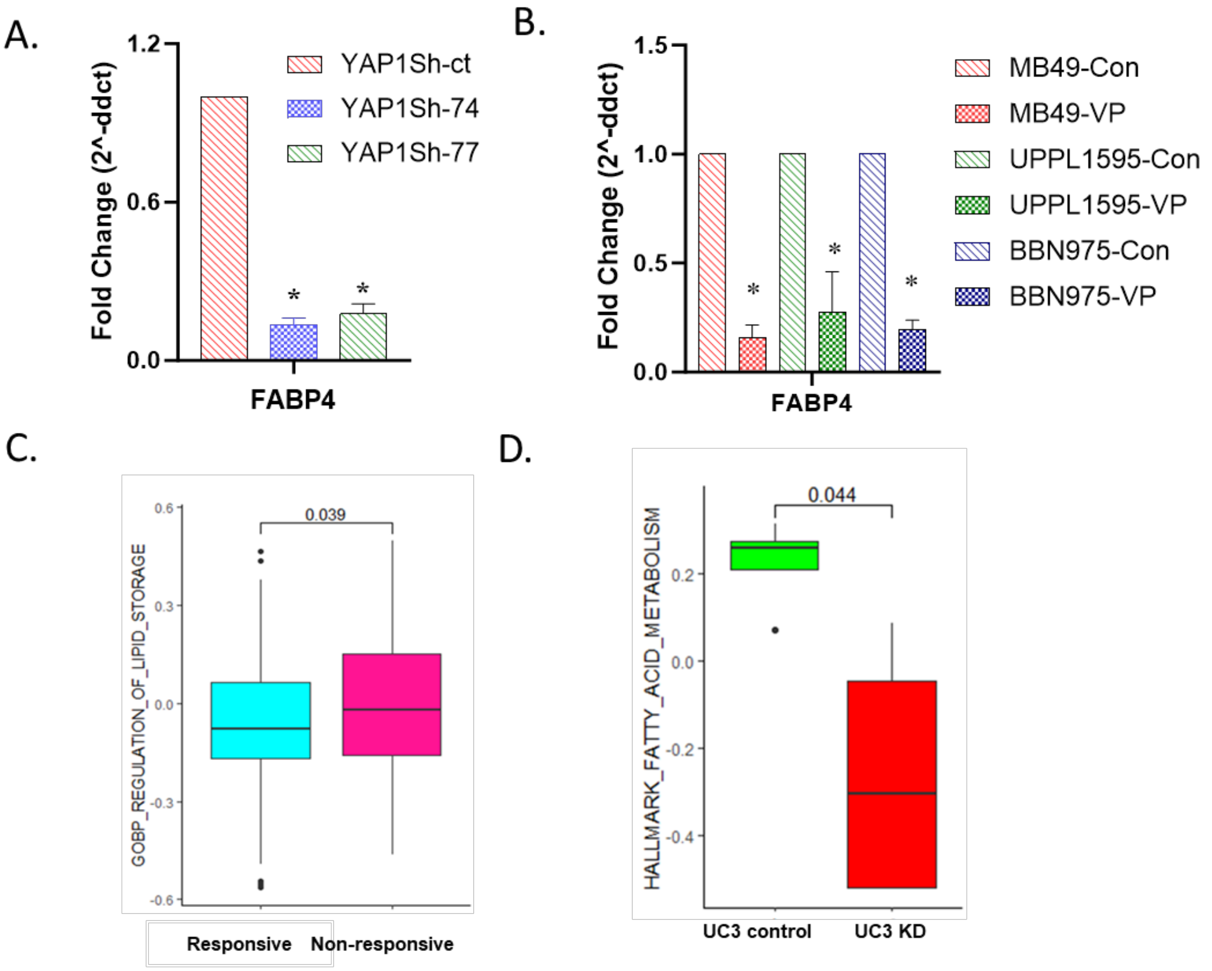
A.
1.2
0.6
0.0
Fold Change (2^-ddct)
YAP1Sh-ct
YAP1Sh-74
YAP1Sh-77
*
*
FABP4
B.
1.5
1.0
0.5
0.0
Fold Change (2^-ddct)
MB49-Con
MB49-VP
UPPL1595-Con
UPPL1595-VP
BBN975-Con
BBN975-VP
*
*
*
FABP4
C.
0.039
GOBP_REGULATION_OF_LIPID_STORAGE
0.6
0.3
0.0
-0.3
-0.6
Responsive
Non-responsive
D.
0.044
HALLMARK_FATTY_ACID_METABOLISM
0.2
0.0
-0.2
-0.4
UC3 control
UC3 KD

Figure S7

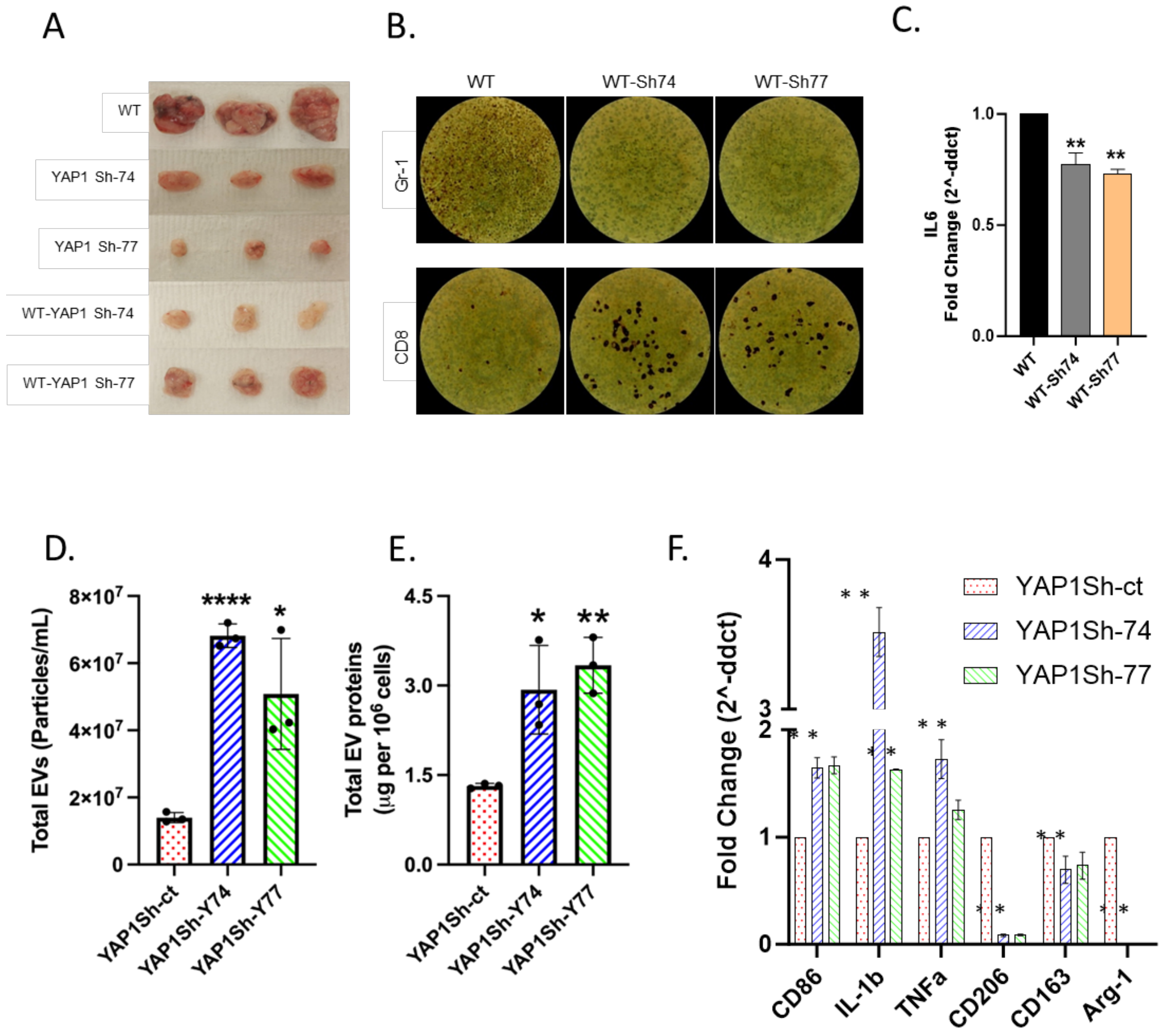

A
WT
YAP1 Sh-74
YAP1 Sh-77
WT-YAP1 Sh-74
WT-YAP1 Sh-77
B.
WT
WT-Sh74
WT-Sh77
Gr-1
CD8
C.
IL6
Fold Change (2^-ddct)
1.0
0.5
0.0
**
**
WT
WT-Sh74
WT-Sh77
D.
Total EVs (Particles/mL)
8×10^7
6×10^7
4×10^7
2×10^7
0
****
*
YAP1Sh-ct
YAP1Sh-Y74
YAP1Sh-Y77
E.
Total EV proteins (µg per 10^6 cells)
4.5
3.0
1.5
0.0
*
**
YAP1Sh-ct
YAP1Sh-Y74
YAP1Sh-Y77
F.
Fold Change (2^-ddct)
4
3
2
1
0
YAP1Sh-ct
YAP1Sh-74
YAP1Sh-77
CD86
IL-1b
TNFa
CD206
CD163
Arg-1

Figure S8

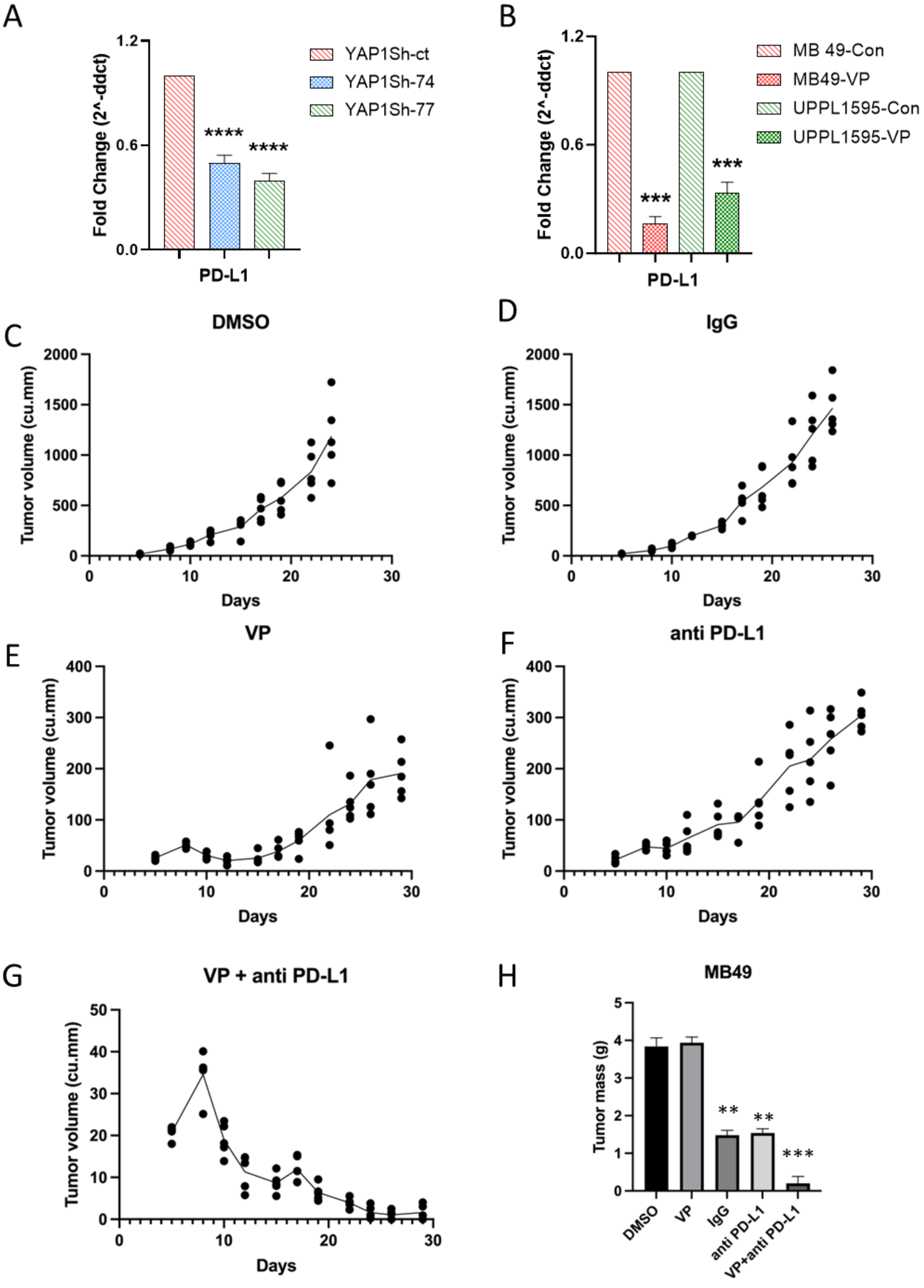
A
Fold Change (2^-ddct)
1.2
0.6
0.0
****
****
PD-L1
YAP1Sh-ct
YAP1Sh-74
YAP1Sh-77
B
Fold Change (2^-ddct)
1.2
0.6
0.0
***
***
PD-L1
MB 49-Con
MB49-VP
UPPL1595-Con
UPPL1595-VP
C
DMSO
Tumor volume (cu.mm)
2000
1500
1000
500
0
0
10
20
30
Days
D
IgG
Tumor volume (cu.mm)
2000
1500
1000
500
0
0
10
20
30
Days
E
VP
Tumor volume (cu.mm)
400
300
200
100
0
0
10
20
30
Days
F
anti PD-L1
Tumor volume (cu.mm)
400
300
200
100
0
0
10
20
30
Days
G
VP + anti PD-L1
Tumor volume (cu.mm)
50
40
30
20
10
0
0
10
20
30
Days
H
MB49
Tumor mass (g)
5
4
3
2
1
0
**
**
***
DMSO
VP
IgG
anti PD-L1
VP+anti PD-L1

Figure S9

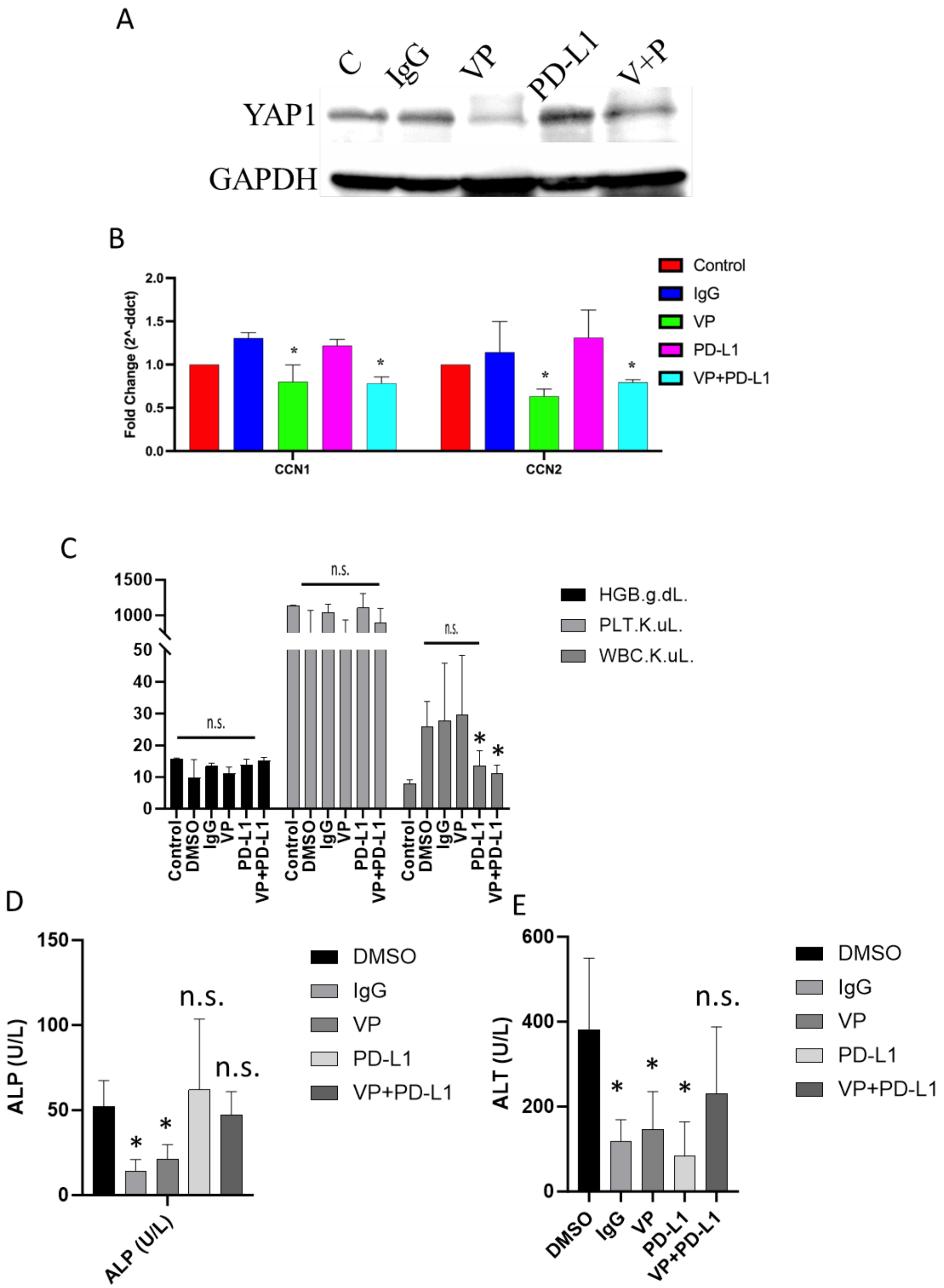
A
C
IgG
VP
PD-L1
V+P
YAP1
GAPDH
B
Fold Change (2^-ddct)
2.0
1.5
1.0
0.5
0.0
CCN1
CCN2
Control
IgG
VP
PD-L1
VP+PD-L1
C
1500
1000
50
40
30
20
10
0
n.s.
HGB.g.dL.
PLT.K.uL.
WBC.K.uL.
Control
DMSO
IgG
VP
PD-L1
VP+PD-L1
D
ALP (U/L)
150
100
50
0
n.s.
DMSO
IgG
VP
PD-L1
VP+PD-L1
E
ALT (U/L)
600
400
200
0
n.s.
DMSO
IgG
VP
PD-L1
VP+PD-L1

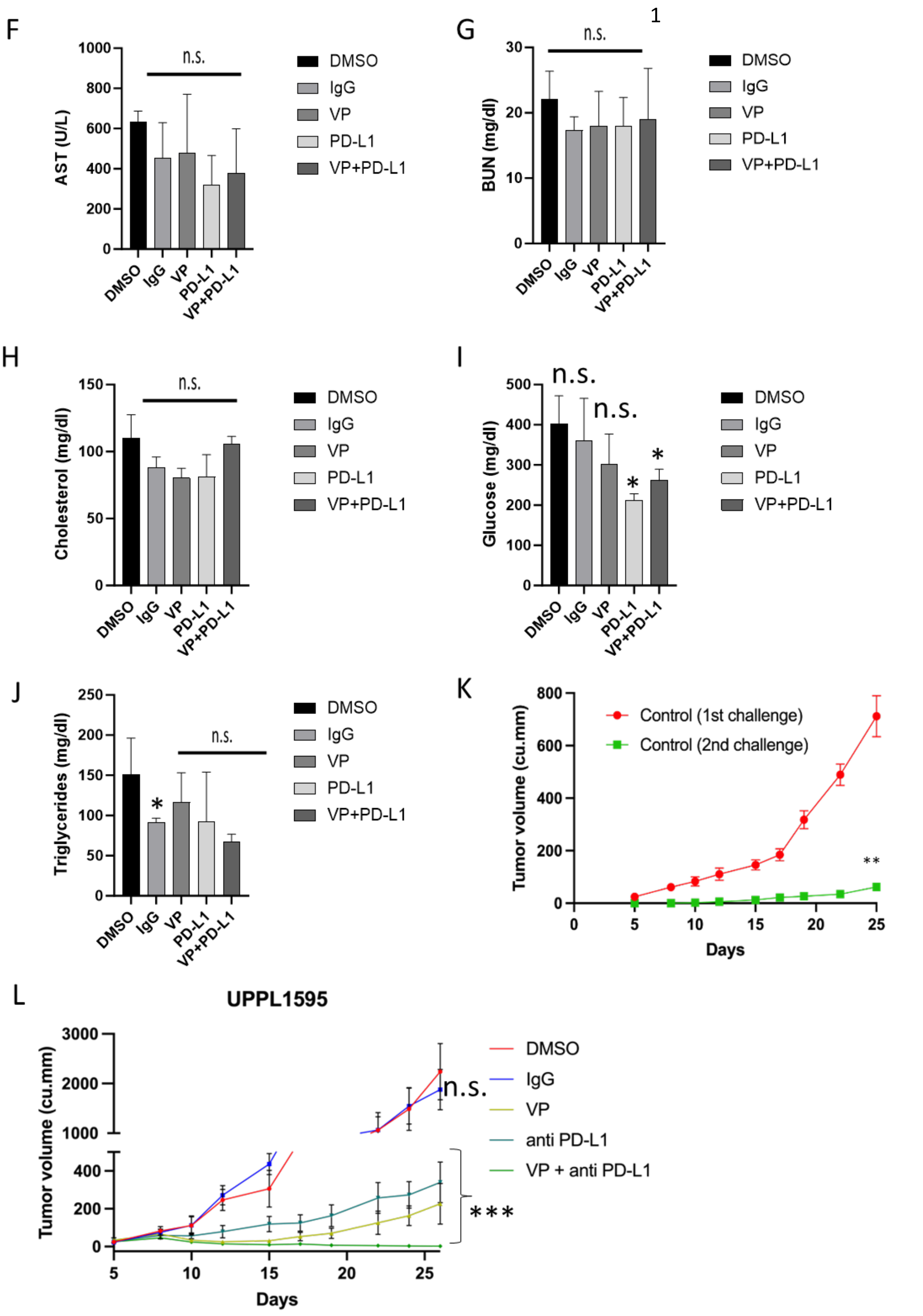
F
AST (U/L)
1000
800
600
400
200
0
n.s.
DMSO
IgG
VP
PD-L1
VP+PD-L1
G
1
BUN (mg/dl)
30
20
10
0
n.s.
DMSO
IgG
VP
PD-L1
VP+PD-L1
H
Cholesterol (mg/dl)
150
100
50
0
n.s.
DMSO
IgG
VP
PD-L1
VP+PD-L1
I
Glucose (mg/dl)
500
400
300
200
100
0
n.s.
n.s.
*
*
DMSO
IgG
VP
PD-L1
VP+PD-L1
J
Triglycerides (mg/dl)
250
200
150
100
50
0
*
n.s.
DMSO
IgG
VP
PD-L1
VP+PD-L1
K
Tumor volume (cu.mm)
800
600
400
200
0
Control (1st challenge)
Control (2nd challenge)
**
0
5
10
15
20
25
Days
L
UPPL1595
Tumor volume (cu.mm)
3000
2000
1000
400
200
0
n.s.
***
DMSO
IgG
VP
anti PD-L1
VP + anti PD-L1
5
10
15
20
25
Days

Supplementary figures legends

Figure S1. Analysis of the TCGA-BLCA database and UCB cells showing the oncogenic relevance of YAP1

A. TCGA-BLCA database analysis showing the overall survival of patients associated with YAP1 expression. Patients were divided into two groups considering the median value as a cut off. N=201 in each group. **B**. Disease free survival of TCGA-BLCA patients considering median expression value of YAP1 as a cutoff. **C.** Pearson correlation coefficient of top YAP1 associated genes across in TCGA-BLCA database. **D.** Expression of the top YAP1 associated genes across different subtypes of UCB in TCGA-BLCA database. **E.** Immunoblots showing YAP1 expression level in different parental (P1, P2), YAP1-sh control clones (CT1, CT2) and YAP1 knockdown (KD) clones (Y85, Y86, Y84, Y77, Y74, Y75) in MB49 cells. **F.** Densitometric analysis of immunoblots showing the YAP1 expression in different mice parental bladder cancer cell lines and after treatment with verteporfin (VP), a potent and specific YAP1 inhibitor (Figure 1F). **G-H**. RT-qPCR analysis of several YAP1 downstream targets in YAP1 KD MB49 cells and VP treated mouse UCB cell lines. The data presented as means ± standard deviation of at least three independent experiments. *, $p < 0.05$; **, $p < 0.01$; ***, $p < 0.001$ by unpaired t test.

Figure S2. Immunoregulatory potential of YAP1 in UCB

A. Fast Gene set enrichment analysis (FGSEA) analysis showed the top 30 downregulated pathways in MB49 YAP1 Sh-Y74 (YAP1 KD) cells compared to MB49 YAP1 Sh-Ct (YAP1 expressing) cells. B. FGSEA analysis showed the top 30 upregulated pathways in MB49 YAP1 Sh-Y74 (YAP1 KD) cells compared to MB49 YAP1 Sh-Ct (YAP1 expressing) cells (padj= p adjusted value). **C-D**. RNA seq data from mouse MB49 YAP1 KD cells showing the downregulation of WNT-βcatenin and EMT signaling pathway. **E-F**. RNA seq data from human UC3 YAP1 KD (public data, GSE186043) cells showing the downregulation of WNT-βcatenin and EMT signaling pathway. **G.** Representative IHC showing the MDSCs (Gr-1) and CD8 T cells in MB49 YAP1 KD and YAP1-Sh-ct xenografts (n=5). **H.** Representative IHC showing notably decreased number of MDSCs (Gr-1) and increase number of CD8 T cells due to pharmacologic inhibition of YAP1 treated with VP. **I.** CDX model developed with MB49 WT cells and treated with anti-Ly6G antibody. The left panel represents the tumor growth rate and the right panel represents the weight of tumor mass. The data presented as means ± standard deviation of at least three independent experiments. *, $p < 0.05$; **, $p < 0.01$; ***, $p < 0.001$ by unpaired t test.

Figure S3. Correlation between M2 macrophages and non-responsiveness to immunotherapy

A. Heatmap showing the expression level of different M2 macrophage markers in the Imvigor210 database samples. CR=complete response, PR=partial response, SD=stable disease, PD=progressive disease. **B.** GSVA (Gene set variation analysis) enrichment score of M2 macrophage

markers in the Imvigor210 database among the Responsive (CR and PR) and Nonresponsive (SD and PD) patient's groups. p value was calculated with the t-test.

Figure S4. Correlation between CXCR2 associated ligands and YAP1 in experimental models and in our primary UCB cohort and TCGA cohort

A. TCGA-BLCA database analysis showing the top overexpressed genes in UCB (left); scatter plot showing CXCL10 (one of the top gene in the left heatmap) expression level in tumor and normal samples of TCGA-BLCA database (right). **B.** RT-qPCR analysis of various chemokines in MB49 YAP1-Sh and sh-control clones. **C.** RT-qPCR analysis of various chemokines in MB49 xenograft developed from MB49 YAP1-Sh and sh-control clones. **D**. RT-qPCR analysis of CXCL2, CXCL3, CXCL 5 and CXCL10 in VP XXXX hrs. treated mice UCB cell lines. **E-H.** RT-qPCR analysis showing the expression level of CXCR2 associated ligands in YAP1 KD human bladder cancer cell lines (BFTC905 and T24) (YAP1-Sh) (E-F) and YAP1 overexpressed human bladder cancer cell lines (BFTC909 and UMUC3) (YAP1LV). **I**. RT-qPCR analysis of CXCL2, CXCL3 and CXCL6 in a primary UCB cohort (n=30) showed significant correlation of YAP1 expression with these ligands. **J**. Correlation of expression data of CXCL2, CXCL3 and CXCL6 with YAP1 expression in TCGA UCB cohort (n=407). The data presented as means ± standard deviation of at least three independent experiments. *, $p < 0.05$; **, $p < 0.01$ by unpaired t test.

Figure S5. YAP1 expression correlates with IL6/STAT3 mediated signaling pathway

A. Overall survival analysis of patients of the Imvigor210 database indicates poor overall survival probability of the high YAP1/IL-6 expressed group (GSVA_high) compared to the low YAP1/IL-6 expressed group (GSVA_low); the survival probability was calculated using GSVA analysis of IL-6 and YAP1 expression. **B.** Overall survival analysis of patients of the Imvigor210 database indicates poor overall survival of the high IL-6 expressed group compared to the low IL-6 expressed group. **C.** Overall survival analysis of patients of the Imvigor210 database indicates poor survival of the high STAT3 expressed group compared to the low STAT3 expressed group. **D-E.** Correlation (Spearman correlation coefficient test) of YAP1 expression with Il-6 and STAT3 in TCGA UCB cohort. **F.** Densitometric analysis of immunoblots showing IL6 expression level of VP-treated CDX xenografted tissues. The data presented as means ± standard deviation of at least three independent experiments, **, $p < 0.01$ by unpaired t test.

Figure S6. YAP1 induces Lipid droplet storage and enhances immune suppression

A. RT-qPCR analysis showing decreased expression of FABP4 in YAP1 KD MB49 clones. **B.** RT-qPCR analysis showing decreased expression of FABP4 in VP treated mouse UCB cell lines (MB49, UPPL595, BBN975). **C.** Analysis of lipid storage in IMVIGOR210 database indicate high lipid storage in immunotherapy non-responsive group. **D.** RNA seq data from human UC3 YAP1 KD (public data, GSE186043) cells showing the downregulation of key genes from Fatty acid metabolism pathway. The data presented in A and B as means ± standard deviation of at least three independent experiments. *, $p < 0.05$; **, $p < 0.01$; ***, $p < 0.001$ by unpaired t test.

Figure S7. YAP1 regulates EV secretion from the MB49 UCB cells

A. Representative tumor images from the co-injection study. **B.** IHC showing the expression of MDSCs (Gr-1) and CD8 T cells in xenografts developed from MB49 WT tumors and co-injected with the MB49 YAP1-Sh clones and controls in the different flank of the same mouse. **C.** IL-6 expression in xenografts developed from MB49 WT tumors and co-injected with the MB49 YAP1-Sh clones and controls. **D**. MB49 YAP1 KD cells were cultured in exosome-depleted condition, and thereafter, extracellular vesicles were isolated and subsequently quantified in a NanoSight 300. **E.** Total EV proteins were isolated from 1 million cells of indicated clones, and total protein was quantified using the BCA method (Pierce™ BCA Protein Assay Kit). **F.** Isolated EVs from the indicated MB49 cell clones were exposed to macrophage cell line RAW264.7 for XX hrs. and macrophage polarization markers were quantified using RT-qPCR. The data presented as means ± standard deviation of at least three independent experiments. *, $p < 0.05$; **, $p < 0.01$; ***, $p < 0.001$, by unpaired t test.

Figure S8. YAP1 attenuation increase the efficacy of anti PD-L1

A-B. RT-qPCR analysis of PD-L1 expression in MB49 YAP1 KD clones (YAP1-sh) and VP treated mice UCB cell lines (MB49 and UPPL1595 cells). Sh-ct=control, VP=VP treated. **C-G.** Tumor growth curve of MB49 WT cells derived xenografts treated with indicated agent/s. **H.** Tumor mass of MB49 WT cell derived xenograft treated with indicted agent/s. The data presented as means ± standard deviation of at least three independent experiments. *, $p < 0.05$; **, $p < 0.01$; ***, $p < 0.001$ by unpaired t test.

Figure S9. VP attenuates YAP1 without any toxic effect in animal

A. Immunoblots showing YAP1 expression level in CDX of C57Bl/6 animals treated with VP, anti-PD-L1 and combination of VP and anti-PDL1 **B.** RT-qPCR analysis of two YAP1 downstream targets in CDX from C57Bl/6 animals, treated with VP and anti-PDL1. **C.** Hemoglobin, Platelet, and White Blood Cell (WBC) count in experimental animals treated with VP and anti-PDL1 (Figure 10B). **D-J**. Different systemic toxicity parameters such as Alkaline Phosphatase (ALP), Alanine Transaminase (ALT), Aspartate Aminotransferase (AST), Blood urea nitrogen (BUN), and different serum biomarkers such as Cholesterol, Glucose, and Triglycerides were quantified for experimental animals treated with VP and anti-PD-L1 (Figure 10B). **K.** Tumor growth curve of MB49 WT cells in previously non-treated C57BL/6 (control) and mice previously treated (completely cured) with VP+anti-PD-L1. **L.** WT UPPL1595 cells were subcutaneously injected into C57BL/6 mice and treated with VP, anti-PD-L1 and combination of VP and anti-PDL1. Tumor growth was monitored at the indicated times. The data presented as means ± standard deviation of at least three independent experiments. *, $p < 0.05$; **, $p < 0.01$; ***, $p < 0.001$ by one-way ANOVA.

Supplementary Materials and Methods

Cell lines, Constructs and Mice

Our study examined male and female animals, and similar findings are reported for both sexes. Several mice (MB49, UPPL1595 and BBN975) and human cell lines (BFTC905, BFTC909, T24 and UMUC3) were used in this study. Mouse MB49 cell line was established from C57BL/6 mice by exposure of primary bladder epithelial cell explants to dimethylbenz (a) anthracene (DMBA) [1]. MB49 cells were maintained in DMEM medium (Mediatech, Manassas, VA, USA) with 10% fetal bovine serum (Hyclone, Logan, UT, USA). BBN975 cell line was derived from BBN induced mice tumors [2]. The BBN975 cells were maintained in RPMI1640 medium (Mediatech, Manassas, VA, USA) with 10% fetal bovine serum. The UPPL1595 cell line was established from mice bladder [3] and maintained in MEM medium (Mediatech, Manassas, VA, USA) with 10% fetal bovine serum, vitamin solution, sodium pyruvate, non-essential amino acids and HEPES. The BFTC 905 and BFTC 909 cell lines were obtained from the German Collection of Microorganisms and Cell Cultures (Braunschweig). T24, and UM-UC3 cell lines were obtained from the ATCC. These cell lines were maintained in DMEM medium (Mediatech, Manassas, VA, USA) with 10% fetal bovine serum (Hyclone, Logan, UT, USA). All the cells were cultured under a 5% CO_2 atmosphere at 95% relative humidity.

YAP1 shRNA pGFP-C-shLenti Vector (YAP1-sh) was used for the knockdown of the gene expression (Origene). Non-effective 29-mer scrambled shRNA pGFP-C-shLenti Vector (Origene) was used as a control (YAP1-Ctrl). For the si-RNA mediated knockdown of YAP1, YAP1 Silencer Select siRNA (Thermo Fisher Scientific) was used.

C57BL/6 mice were obtained from Charles River Laboratories (Frederick, USA). NSG mice were obtained from the internal animal facility, Johns Hopkins University. Mice were maintained under pathogen-free conditions within the Johns Hopkins Medical Institutes animal care facility in accordance with the American Association of Laboratory Animal Care Guidelines.

Cell viability assay

Cell proliferation and viability were evaluated using alamarBlue™ Cell Viability Reagent (ThermoFischer Scientific). Briefly, cells ($5x10^3$/well) were seeded into 96-well plates with culture media containing 10% FBS and the optical density of each well was measured following the manufacturers protocol. The absorbance was measured at desired time interval by a Spectra Max 250 Plate Reader (Molecular Devices). Cell viability were calculated as percentage over control.

Sphere formation assay

Sphere formation was induced by culturing cells (2×10^4/well) in DMEM/Ham's F12 50/50 Mix (Mediatech) supplemented with B-27 (Life Technologies), 20 ng/mL FGF-basic (Peprotech), 20 ng/mL EGF (Peprotech). Cell culture was performed in ultra-low attachment 6 well plates (Corning, Lowell, USA) for 10 days. The medium was replaced every other day. Sphere formation was evaluated using the inverted phase-contrast microscope.

1

Transcriptomic and gene expression signature analysis

Total RNA from the cultured cells were extracted using Monarch® Total RNA Miniprep Kit (NEB# T2010S). RNA quality was evaluated using Agilent Tape Station using High Sensitivity D1000 Screen Tape. RNA-seq libraries were prepared using the NE Next® Ultra™ II RNA Library Prep Kit for Illumina® (NEB # E7775) from total RNA, following the manufacturer's protocol. Libraries were then sequenced on a NovaSeq 6000 using 2x50 -bp paired-end reads to an average depth of ~52 million reads per sample. Transcriptomic data collected by RNA sequencing (RNA-seq) was analyzed to determine the genes that are present in each sample and condition, their expression levels, and the differences between expression levels among experiment conditions, as follows. Sequencing reads were mapped to the mouse genome version GRCm39 with the alignment tool STAR v.2.7.6a [4], which allows for large 'gaps' in the alignment, representing introns. The aligned reads were assembled withPsiCLASSv.1.0.2 [5] to create gene and transcript models. Transcripts were then assigned to known genes from the GENCODE vM.28 reference gene set. Lastly, DESeq2 [6] was used to quantify the expression levels and determine differentially expressed genes. Additional visualizations, including volcano plots and plots of principal coordinate analysis (PCA) components, were generated with custom R scripts. Differentially expressed genes (DEGs) between YAP1 Sh-KD versus YAP1 Sh-ct cells were defined as $\log2(FC) > 1$ (up) or $\log2(FC) < -1$ (down) and $FDR < 0.05$. Data have been deposited in NCBI GEO (accession number GEO23174).

Bioinformatics Analysis

Overall survival and disease free survival analysis in bladder cancer patients with respect to YAP1 expression was done using the webbased software GEPIA [7]. IMVigor210 RNAseq data and clinical outcomes were downloaded from R package "IMvigor210CoreBiologies" [7]. To evaluate the association between immunotherapy responses and expressions of YAP1 and IL6, GSVA analysis was performed. Samples were equally separated into "GSVA high" and "GSVA_low" groups based on their GSVA enrichment score. To generate heatmap and boxplot based on responses, samples of "NE" (not estimable) in "Best Confirmed Overall Response" were excluded. Samples with "CR" and "PR" were considered as "Response" group, while samples with "SD" and "PD" as "Nonresponse" group. GSE186043 cohort data were downloaded from GEO (Gene Expression Omnibus) to evaluate the effect of YAP1 knockdown in UC3 cell line.

Bioinformatic analysis and visualization were performed with R packages including: "DESeq2" [8] to normalize sequencing data and calculate padj value, "ggplot2" [9] to generate box plots and barplots, "ComplexHeatmap" [10] and "circlize" [11] to generate heatmaps, "GSVA" [12] to calculate GSVA enrichment scores, "survminer" [13] and "survival" [14] to generate survival curves, "fgsea" to calculate Normalized Enrichment Scores (NESs) and padj values for gene sets to generate bar plots [8]. p values shown in boxplots were calculated with t test (two groups) or Anova (four groups).

Gene sets used for analysis included Interleukin_pathway_genes, M2_macrophage, and gene sets for pathways of interests were downloaded from GSEA website (www.gsea-msigdb.org), such as HALLMARK_IL6_JAK_STAT3_SIGNALING (v2022.1.Mm), HALLMARK_GLYCOLYSIS (v2022.1.Hs; v2022.1.Mm), HALLMARK_ WNT_BETA_CATENIN_SIGNALING (v2022.1.Hs; v2022.1.Mm), HALLMARK_EPITHELIAL_MESENCHYMAL_TR- ANSITION (v2022.1.Hs; v2022.1.Mm), HALLMARK_FATTY_ACID_METABOLISM (v2022.1.Hs),

GOBP_REGULATION_OF_LIPID_STORAGE (v2022.1.Hs), and mouse MSigDB collection m5.all.v2023.2.Mm.symbols.

The cytotoxic T-lymphocyte assay (CTL assay)

CD8+ T cells were isolated from the spleen of WT C57BL/6 mice using the Mouse CD8 T Cell Isolation Kit (Biolegend, 480008) and activated using Recombinant Mouse IL-2 Protein (30 U/mL). MB49 YAP1 KD clones (YAP1_Sh-Ct, Sh-74, Sh-77) were plated in a 96-well plate. After overnight incubation, activated CD8+ T cells were added to each well at a ratio of 1:1, 1:5 and 1:10 (MB49: CD8 + T cells). Following incubation for 16h, the plates were centrifuged at 400g for 5 min. The supernatants in each group were used for Lactate dehydrogenase (LDH) release assay (CytoSelect™ LDH Cytotoxicity Assay Kit) following manufacturer's instructions. The absorbance was detected at 490nm using a Spectra Max 250 Plate Reader (Molecular Devices).

Macrophage isolation and migration assay

Eight-week-old C57BL/6 mice were intraperitoneally injected with 3% Brewer thioglycollate medium and intraperitoneal macrophages were harvested 3 days after treatment. Cell migration assay was performed using a transwell co-culture system in 24-well plates (Corning) as described previously [9]. For the cell migration assay, primary macrophages were seeded at a density of 1.0 x 10^6 cells/well to top wells with an 8.0-mm pore size, co-cultured with conditioned media (CM) from different cultured MB49 clones (YAP1Sh-Ct, YAP1Sh-74, YAP1Sh-77) in bottom wells for 24 hours. Migrated cells were stained and counted.

MDSC isolation and migration assay

MDSCs were isolated from the tumor mass developed from MB49 YAP1-Sh and Sh-control clones using a Mouse MDSC Isolation Kit (Miltenyi Biotec, Cat# 130-094-538) and plated in RPMI1640 supplemented with 10% FBS and antibiotics. MDSCs (1 x 10^5 cells/well) were seeded in the top chamber of the transwell (Corning) plate. Conditioned media (CM) from cultured MB49 clones (YAP1Sh-Ct, Sh-74, Sh-77) were collected and added to the bottom layer of the transwell. After 4 hrs incubation, completely migrated cells to the bottom chamber were counted.

Enzyme-Linked Immunosorbent Assay (ELISA)

Cells (2 x 10^6/100 mm dish) were cultured for 24 hrs. Media were removed and replaced with 10 ml serum-free DMEM. Supernatants were collected after 24 hrs. The floating cells removed by 0.45mm filtration. ELISA was performed for IL-10 and TNFα according to the manufacturer's instructions (R&D Systems, USA). For IL-10 and TNFα ELISA was performed using conditioned medium. For IL-6 and STAT3 cell lysates were prepared, and ELISA was performed according to the manufacturer's instructions (RAB0309 for IL6; ab126459 for STAT3).

Immunohistochemistry (IHC)

Tumor tissues from mouse models were collected and fixed in 10% formalin overnight. The formalin fixed tissue was transferred to the internal core facility and standard protocol was followed for IHC using antibodies for Gr-1 (MDSC marker) and CD8+T cells. The slides were micro graphed under 40X magnification using EVOS FL auto microscope of life technologies.

In vivo xenograft assay and treatment

For in vivo xenograft, cells were suspended in 100 µL of a 1:1 mixture of serum-free DMEM and Cultrex Stem Cell Qualified Reduced Growth Factor Basement Membrane Extract (Trevigen, Gaithersburg, USA) and then injected subcutaneously into the flank of C57BL/6 mice for MB49/ UPPL1595/ BBN975 cells.

For treatment, animals were randomized to different treatment groups (n=5) after a week of cell implantation. Verteporfin (Sigma-Aldrich; #1711461) (50 mg/kg) was administered via *i.p.* injections every alternative day and S3I-201 (MedKoo; #202541) was administered via *i.p.* injections everyday (5 mg/kg). Mice were also treated with equal volume of DMSO as control. Anti-PD-L1 monoclonal antibody (B7-H1), anti-Ly6G (anti-MDSCs) monoclonal antibody (1A8) (Bio X Cell, West Lebanon, USA) and the corresponding isotype antibody (200µg/mouse) were administered via i.p. injection every 3 days. Tumor tissue was collected when the mice were euthanize following the protocol.

All experiments using mice were approved by the Johns Hopkins University Animal Care and Use Committee, and the mice were maintained in accordance with the American Association of Laboratory Animal Care Guidelines.

Isolation of immunocytes from mouse organs and tumor tissue

Tumors were minced and digested with collagenase type IV (Sigma-Aldrich), hyaluronidase (Sigma-Aldrich), and DNase type IV (Sigma-Aldrich) into HBSS (Hank's Balanced Salt Solution, Thermo Fisher), followed by depletion of red blood cells (RBCs) using ACK (Ammonium-Chloride-Potassium) lysing buffer (Quality Biological, Gaithersburg, USA). Discontinuous (44% and 67%) Percoll PLUS (GE Healthcare) separation method was used to enrich immunocytes. Bone marrow were collected from tibia and femur. Whole blood was collected from right ventricle. Excised spleen was smashed on 70 µm cell strainer. The RBCs in blood, bone marrow, and spleen were lysed with ACK lysing buffer. CD11b+Ly6G high MDSCs and CD8+ T-cells were isolated using Myeloid-Derived Suppressor Cell Isolation Kit (Miltenyi Biotec) and CD8a+ T Cell Isolation Kit (Miltenyi Biotec), respectively, according to the manufacturer's instructions.

Flow cytometric analysis

Tumors developed from the MB-49 YAP1 sh clones were weighed and mechanically minced into smaller pieces. Then tumor pieces were incubated in DNase I (50ug/mL, Millipore Sigma) and collagenase P (2 mg/mL, Millipore Sigma) for 20 minutes at 37°C. The dissociated cells were passed through a 70 µm cell strainer (BD). The cells were centrifuged, and the supernatant was discarded. The pellet was resuspended in 40% Percoll solution and overlaid on 80% Percoll solution (GE Healthcare) in a 15 ml conical centrifuge. The solution was centrifuged for 30 minutes at 3000 rpm at room temperature. The cells were harvested from the 80%/40% Percoll interface and washed twice with FACS buffer and resuspend in 1X PBS. Then, the cells were stained with zombie aqua viability dye (Biolegend, San Diego, CA, CAT# 423102) according to the manufacturer's instructions. Upon removal of the Zombie dye buffer, the Fc receptors were blocked with TruStain FcX (Biolegend, San Diego, CA, CAT# 101320). Cells were then incubated for 30 minutes at 4°C for surface marker targets listed in supplementary **table 1**. Following this, according to the manufacturer's protocol, intracellular staining was performed for proteins of

interest (Supp. Table 1) following the BD Cytofix/Cytoperm (Catalog BD554714, BD Biosciences, San Jose, CA). Gating schemes followed were : epithelial cells (ZNIR$^-$CD45$^-$), MDSCs (ZNIR$^-$CD45$^+$CD11b$^+$), CD8$^+$ T cells (ZNIR$^-$ CD3$^+$ CD45$^+$ CD8$^+$), CD4$^+$ T cells (ZNIR$^-$ CD3$^+$ CD45$^+$ CD4$^+$), FOXP3$^+$ cells (ZNIR$^-$ CD3$^+$ CD45$^+$ CD4$^+$ CD25$^+$ FOXP3$^+$), IFNγ$^+$ T cells (ZNIR$^-$ CD3$^+$ CD45$^+$ CD8$^+$IFNγ$^+$), CD107$^+$ T cells (ZNIR$^-$ CD3$^+$ CD45$^+$ CD8$^+$ CD107$^+$), CXCR2$^+$ cells (ZNIR$^-$ CD45$^+$ CD11b$^+$ CXCR2$^+$) . Data were acquired on a BD FACSCalibur flow cytometer (BD Biosciences) using BD CellQuest Pro software (BD Biosciences) and analyzed with FlowJo software v10.1 (BD Biosciences, San Jose, CA).

Table 1. Flow cytometric panel design for tumor samples developed from MB49 YAP1 clones

Flow cytometric panel design for macrophages			
Antibody	Clone	Conjugate	Company
CD3	REA641	PE	Miltenyi Biotec
CD45	30F11	APC/ FITC	Miltenyi Biotec
CD11b	M1/70.15.11.5	FITC	Miltenyi Biotec
Ly6G	REA526	Vio770	Miltenyi Biotec
Ly6C	REA796	PE	Miltenyi Biotec
FOXP3	REA788	Vio700	Miltenyi Biotec
CD8a	536.7	Vio770	Miltenyi Biotec
CD4	GK1.5	FITC	Miltenyi Biotec
CD107a	1D4B	FITC	Miltenyi Biotec
IFNg	XMG1.2	APC	Biolegend
CXCR2	SA044G4	APC	Biolegend

RAW 264.7 macrophages were cultured with conditioned media obtained from confluent MB49 YAP clones (constructs sh-ct, sh-74 or sh-77) for 24 hours in 24-well format. The macrophages were treated with 0.05% Trypsin-EDTA (Thermo Fisher Scientific, Waltham, MA, CAT# 253000054) and resuspended in DMEM (with 10% FBS) in a U-bottom 96-well plate for subsequent flow cytometric staining. The macrophages were first stained with zombie aqua viability dye (Biolegend, San Diego, CA, CAT# 423102) according to manufacturer's instructions. Cells were washed to remove viability dye, and the Fc receptors were blocked with TruStain FcX (Biolegend, San Diego, CA, CAT# 101320). Cells were then stained for 30 minutes at 4°C for extracellular targets of interest according to panel shown in suppl. table 2. Intracellular staining was then performed for cytokines of interest using BD Cytofix/Cytoperm (Catalog BD554714, BD

Biosciences, San Jose, CA) according to the manufacturer's protocol. Macrophage cells were sorted as Aqua-F480+CD11b+. Among the live macrophage population, frequencies of CD163+, IL6+, iNOS+, TGFβ+, PDL1+, and MHCII+ cells were quantified. Samples were individually run on a BD LSRII flow cytometer (Becton Dickinson, Franklin Lakes, NJ) utilizing FACSDiva software v6 and analyzed using FlowJo v10 (BD Biosciences, San Jose, CA).

Table 2. Flow cytometric panel design for macrophages treated with MB49-conditioned media

Flow cytometric panel design for macrophages			
Antibody	Clone	Conjugate	Company
Viability	-	Aqua	Biolegend
F4/80	BM8	BV785	Biolegend
CD11b	M1/70	AF700	Biolegend
CD163	TNKUPJ	SB436	eBioscience
I-A/I-E (murine MHC II)	M5/114.15.2	APC/Fire750	Biolegend
PD-L1 (CD274)	10F.9G2	PE/Dazzle™ 594	Biolegend
iNOS	CXNFT	PE-Cy7	eBioscience
TGF-β1 (LAP)	TW7-16B4	FITC	Biolegend
IL-6	MP5-20F3	PE	Biolegend

Isolation and Quantification of Extracellular vesicles (EVs):

MB49 clones (MB49 YAP1 sh-ct, YAP1sh-74 and YAP1sh-77) were seeded in 150 mm culture plate and incubated in DMEM supplemented with 10% Exosome-Depleted FBS (Thermo Fisher) for 24 hrs at 37° C to allow cell attachment. The cells were then washed with PBS twice, and culture medium was switched to 35 mL of DMEM without serum. After incubation for 48 hrs, conditioned medium was collected and centrifuged at 2,000 g for 10 min at 4° C to thoroughly remove cell debris. The resulting supernatant was then filtered through a 0.22 μm PVDF filter (Millipore, #SLGV033RB) to remove cell debris and microvesicles. The flow-through was transferred into ultracentrifuge tubes (Beckman Coulter, #344058) and then ultracentrifuged in a Beckman SW32Ti rotor at 30,000 rpm for 90 min at 4° C. The resulting pellets were washed with 35 mL of ice-cold PBS and then ultracentrifuged again at 30,000 rpm for 90 min at 4° C. The resulting EV pellets were re-suspended in ice cold PBS for experimental use. Protein concentrations of EVs were determined using Micro BCA Protein Assay Kit (Thermo, #23235). Nanoparticle tracking analysis was performed using NanoSight NS300 system (Malvern Instruments, Ranch Cucamonga, CA, USA).

RNA extraction and quantitative reverse transcriptase polymerase chain reaction (RT-qPCR)

Total RNA from the mouse and human UCB cellswere isolated using the RNeasy Plus Mini Kit (Qiagen, Germantown, USA), according to the manufacturer's protocol. This total RNA was converted to cDNA using the SuperScript III First-Strand Synthesis System (Life technologies, Carlsbad, USA), which was then used as a template for RT-qPCR. RT-qPCR was performed using the Fast SYBR Green Master Mix (Thermo Fisher Scientific, Waltham, USA) on a Quant studio 6 Fast Real-Time PCR System (Life Technologies) in triplicate. SDS software (Applied Biosystems) was used to determine cycle threshold (Ct) values. The expression levels were quantified relative to β-actin using the $2^{-\Delta\Delta Ct}$ method. The primer sequences are given in suppl. **table 3.**

Table 3. Primer sequences for the primers used in this study

Gene name	Forward Primer	Reverse Primer
SOX2	AACGGCAGCTACAGCATGATGC	CGAGCTGGTCATGGAGTTGTAC
ALDH2	GCTGTTGTACCGATTGGCGGAT	GCGGAGACATTTCAGGACCATG
FOXA2	CGAGCACCATTACGCCTTCAAC	AGTGCATGACCTGTTCGTAGGC
NOTCH1	GCTGCCTCTTTGATGGCTTCGA	CACATTCGGCACTGTTACAGCC
GJB1	GTGGACCTATGTCATCAGTGTGG	GGAAGGCTTCACACTTGACCAG
CCN1	GTGAAGTGCGTCCTTGTGGACA	CTTGACACTGGAGCATCCTGCA
CCN2	TGCGAAGCTGACCTGGAGGAAA	CCGCAGAACTTAGCCCTGTATG
DECR1	CCAGTGTGTGATAGCCAGCAGA	TCAGGATCTCGAACATCACACCG
CD47	GGTGGGAAACTACACTTGCGAAG	CTCCTCGTAAGAACAGGCTGATC
PTGS1	GAATGCCACCTTCATCCGAGAAG	GCTCACATTGGAGAAGGACTCC
WNT4	GAGAACTGGAGAAGTGTGGCTG	CTGTGAGAAGGCTACGCCATAG
NLRP1	GCTGAATGACCTGGGTGATGGT	CTTGGTCACTGAGAGATGCCTG
CCL20	GTGGGTTTCACAAGACAGATGGC	CCAGTTCTGCTTTGGATCAGCG
COX2	GCGACATACTCAAGCAGGAGCA	AGTGGTAACCGCTCAGGTGTTG
KRT80	CTGGATGCTGAGTGTCTCCGAA	CGTCCTTCACTTGGGCTGTAAG
GLYR1	GAGTTCCTCAGGAGAGCCAAAG	GCTTGCGTTTCTCATCACCTGAG
PXPM2	CCAAGATGAGGAGTGGCTTCTG	GGTAGGCATACCAGAACAGAGC
KRT13	GACTGGCATCTGAAACAGAGCC	TTGTCCGTGGTGGCTTCCAGAA
KAT14	CCATCAACTCCATGTGCCAGGA	CAGGAACCATGAAGCCAAAGGC
H-2K	GGCAATGAGCAGAGTTTCCGAG	CCACTTCACAGCCAGAGATCAC
CD80	CCTCAAGTTTCCATGTCCAAGGC	GAGGAGAGTTGTAACGGCAAGG
H2-Ab	GTGTGCAGACACAACTACGAGG	CTGTCACTGAGCAGACCAGAGT
INOS	GAGACAGGGAAGTCTGAAGCAC	CCAGCAGTAGTTGCTCCTCTTC
Mer-TK	ATCATCCTCGGCTGCTTCTGTG	ACGACCAGTTGGGAATCCTCCT
IL-10	CGGGAAGACAATAACTGCACCC	CGGTTAGCAGTATGTTGTCCAGC
STAT3	AGGAGTCTAACAACGGCAGCCT	GTGGTACACCTCAGTCTCGAAG
CD163	GGCTAGACGAAGTCATCTGCAC	CTTCGTTGGTCAGCCTCAGAGA

CD206	GTTCACCTGGAGTGATGGTTCTC	AGGACATGCCAGGGTCACCTTT
Arg-1	CATTGGCTTGCGAGACGTAGAC	GCTGAAGGTCTCTTCCATCACC
CD86	ACGTATTGGAAGGAGATTACAGCT	TCTGTCAGCGTTACTATCCCGC
TNF-α	GGTGCCTATGTCTCAGCCTCTT	GCCATAGAACTGATGAGAGGGAG
IL-1β	TGGACCTTCCAGGATGAGGACA	GTTCATCTCGGAGCCTGTAGTG
CXCL2	CATCCAGAGCTTGAGTGTGACG	GGCTTCAGGGTCAAGGCAAACT
CXCL3	TGAGACCATCCAGAGCTTGACG	CCTTGGGGGTTGAGGCAAACTT
CXCL10	ATCATCCCTGCGAGCCTATCCT	GACCTTTTTTGGCTAAACGCTTTC
CXCL5	CCGCTGGCATTTCTGTTGCTGT	CAGGGATCACCTCCAAATTAGCG
YAP1	CCAGACGACTTCCTCAACAGTG	GCATCTCCTTCCAGTGTGCCAA
Fabp4	TGAAATCACCGCAGACGACAGG	GCTTGTCACCATCTCGTTTTCTC

Estimation of intracellular lipid droplets and glycolytic activity

Intracellular lipid droplet accumulation was measured in cultured cells following the manufacturer's protocol (Cayman chemical, Item # 500001). Oleic acid was used as a positive control. The cells were micro graphed using EVOS FL Auto microscope by life technologies under FITC filter and fluorescence intensity was detected with Spectramax by Molecular devices. Glycolytic activity was measure in the cell culture media of cultured cells following the manufacturer's protocol (Cayman chemical, Item # 600450). The absorbance was measured at 490 nm with Spectramax by Molecular devices.

References:

1. Denis, M., et al., In Vivo Syngeneic Tumor Models with Acquired Resistance to Anti-PD-1/PD-L1 Therapies. Cancer Immunol Res, 2022. **10**(8): p. 1013-1027.
2. Beckford Vera, D.R., et al., Immuno-PET imaging of tumor-infiltrating lymphocytes using zirconium-89 radiolabeled anti-CD3 antibody in immune-competent mice bearing syngeneic tumors. PLoS One, 2018. **13**(3): p. e0193832.
3. Saito, R., et al., Molecular Subtype-Specific Immunocompetent Models of High-Grade Urothelial Carcinoma Reveal Differential Neoantigen Expression and Response to Immunotherapy. Cancer Res, 2018. **78**(14): p. 3954-3968.
4. Dobin, A., et al., STAR: ultrafast universal RNA-seq aligner. Bioinformatics, 2013. **29**(1): p. 15-21.
5. Song, L., et al., A multi-sample approach increases the accuracy of transcript assembly. Nature communications, 2019. **10**(1): p. 5000.
6. Anders, S. and W. Huber, Differential expression analysis for sequence count data. Nature Precedings, 2010: p. 1-1.
7. Tang, Z., et al., GEPIA: a web server for cancer and normal gene expression profiling and interactive analyses. Nucleic Acids Research, 2017. **45**(W1): p. W98-W102.
8. Korotkevich, G., et al., Fast gene set enrichment analysis. BioRxiv, 2016: p. 060012.
9. Mariathasan, S., et al., TGFβ attenuates tumour response to PD-L1 blockade by contributing to exclusion of T cells. Nature, 2018. **554**(7693): p. 544-548.
