# Supplementary figures and images for: YAP1 induces bladder cancer progression and promotes immune evasion through IL-6/STAT3 pathway and CXCL deregulation
Source: J Clin Invest. 2024 Nov 21;135(2):e171164. doi: 10.1172/JCI171164 (PMC11735109; doi:10.1172/JCI171164)

Figure 1F

YAP1

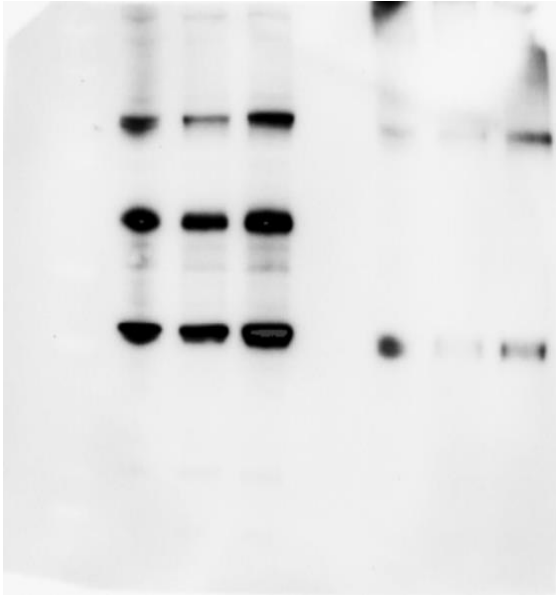

ACTB

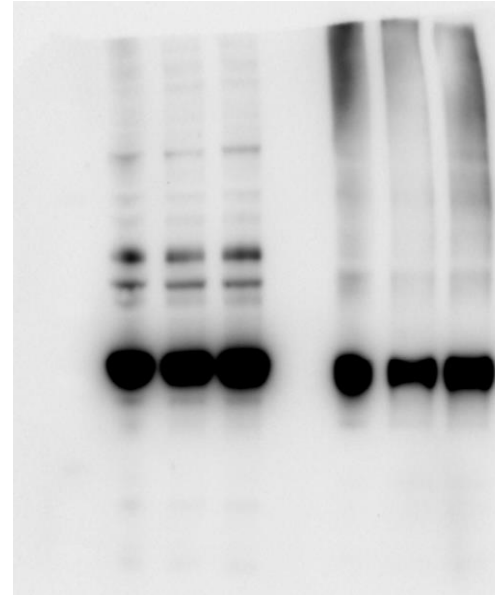

Figure 2F

YAP1

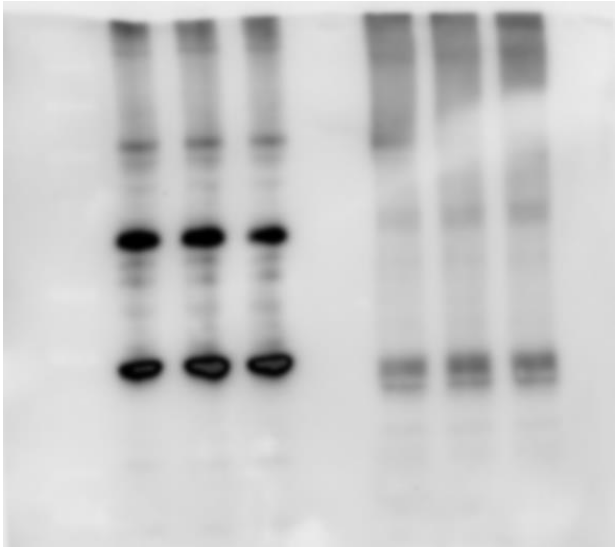

ACTB

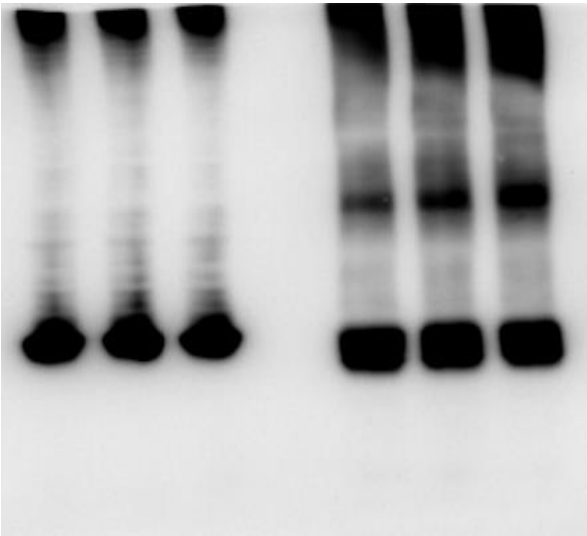

Figure 6H

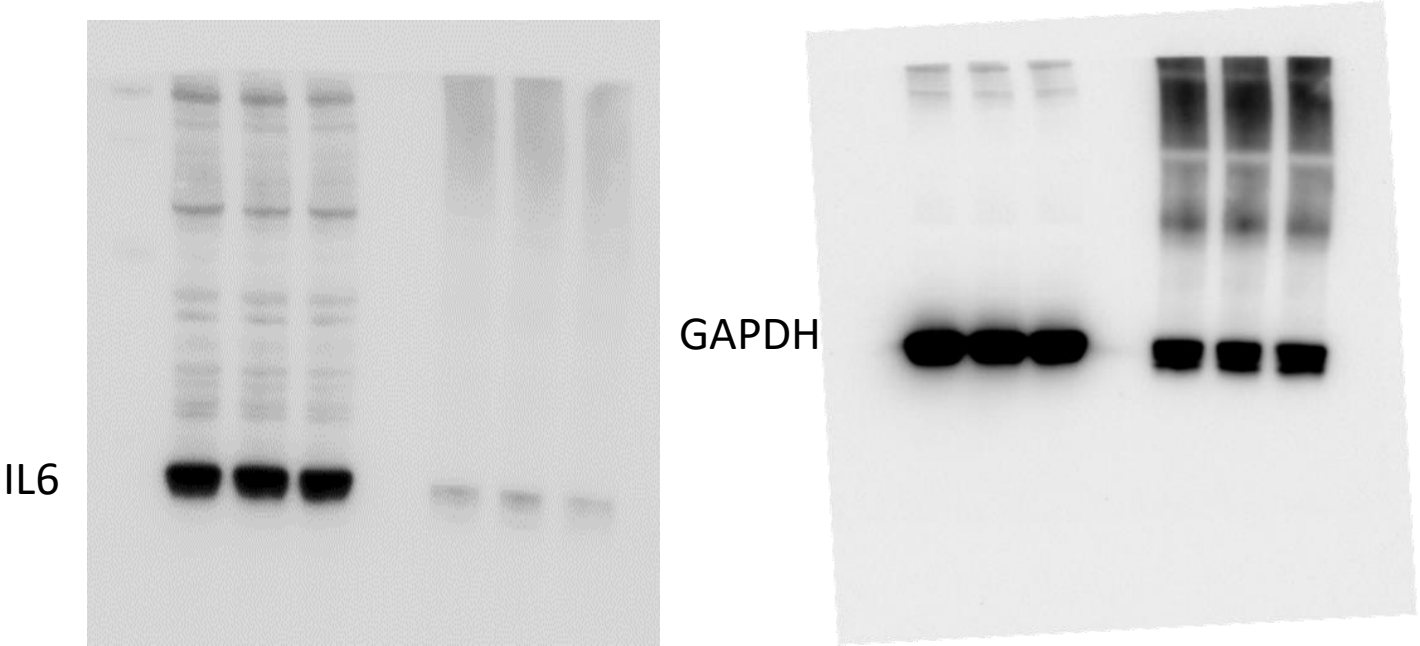

Figure S1E

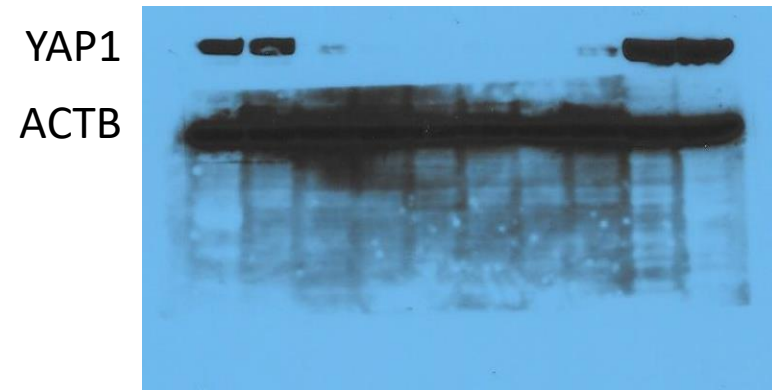

Figure S9A

YAP1

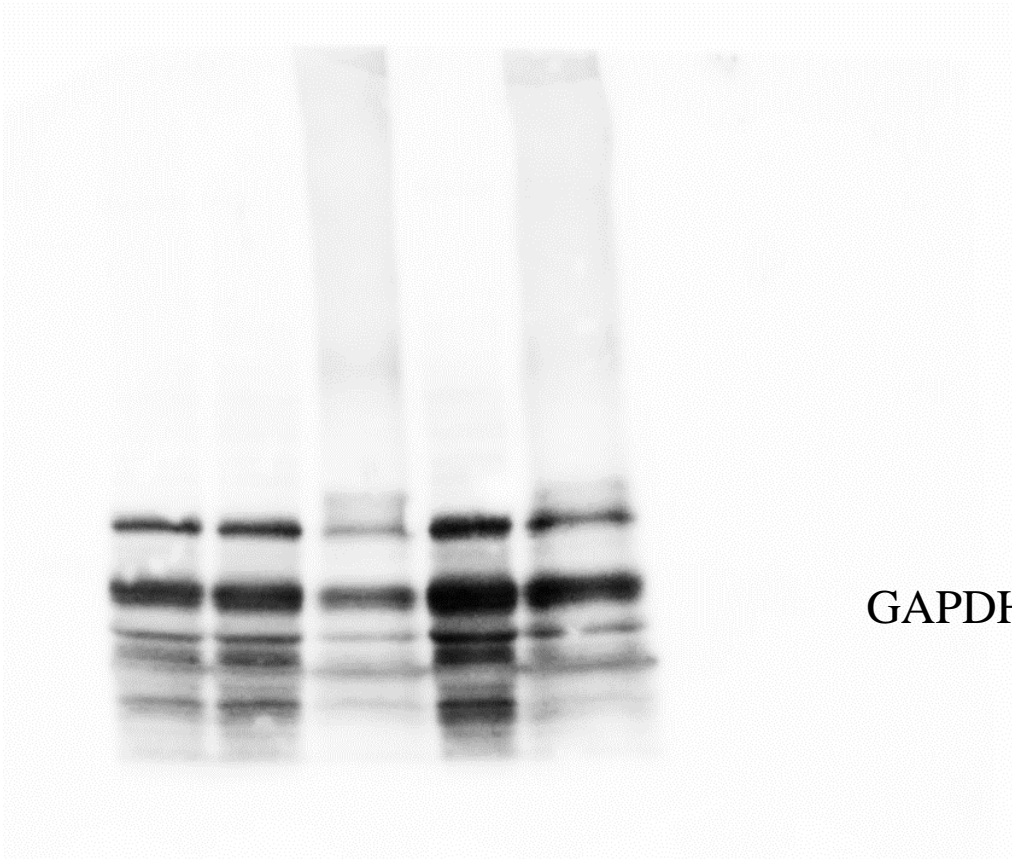

GAPDH

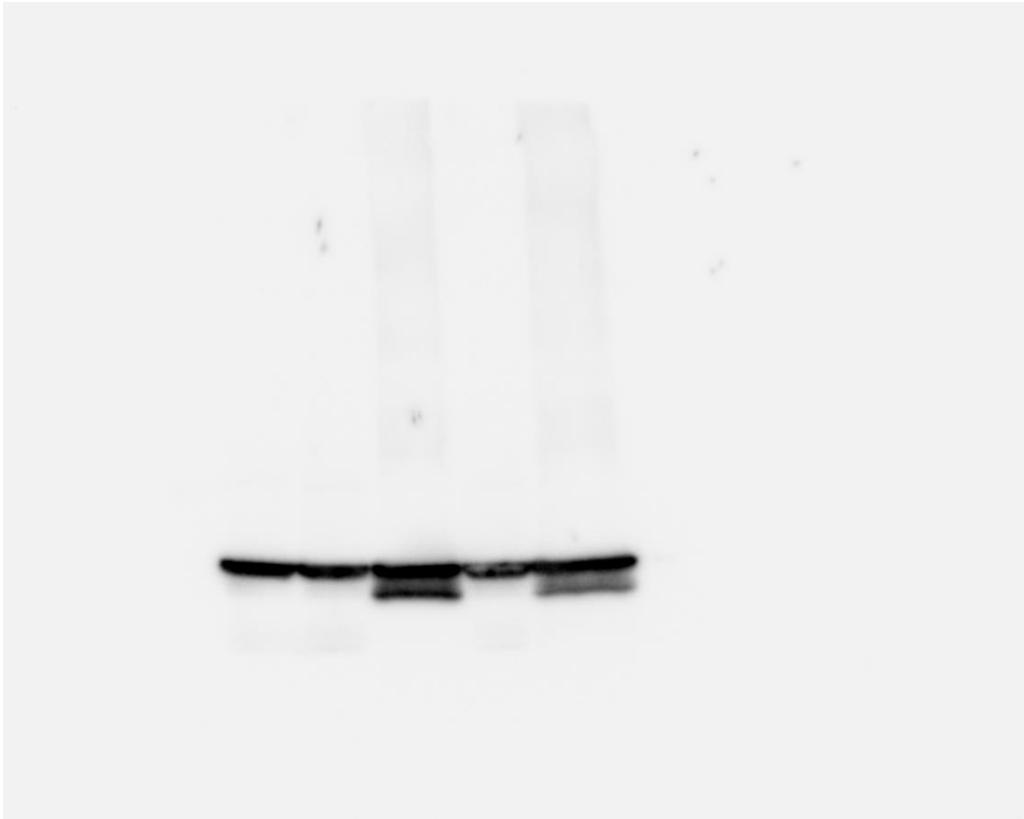

Supplement: Unedited blot and gel images [file jci-135-171164-s013.pdf]
